# Total synthesis of malagashanine: a chloroquine potentiating indole alkaloid with unusual stereochemistry†
†Electronic supplementary information (ESI) available: Experimental details and spectral data are provided. CCDC 1489617. For ESI and crystallographic data in CIF or other electronic format see DOI: 10.1039/c6sc03578g


**DOI:** 10.1039/c6sc03578g

**Published:** 2016-09-19

**Authors:** A. Kong, D. E. Mancheno, N. Boudet, R. Delgado, E. S. Andreansky, S. B. Blakey

**Affiliations:** a Department of Chemistry , Emory University , 1515 Dickey Drive , Atlanta , GA 30322 , USA . Email: sblakey@emory.edu

## Abstract

The first total synthesis of malagashanine, a chloroquine potentiating indole alkaloid, is presented. A highly stereoselective cascade annulation reaction was developed to generate the tetracyclic core of the Malagasy alkaloids. This chemistry is likely to be broadly applicable to the synthesis of other members of this stereochemically unique family of natural products.

## Abstract 2

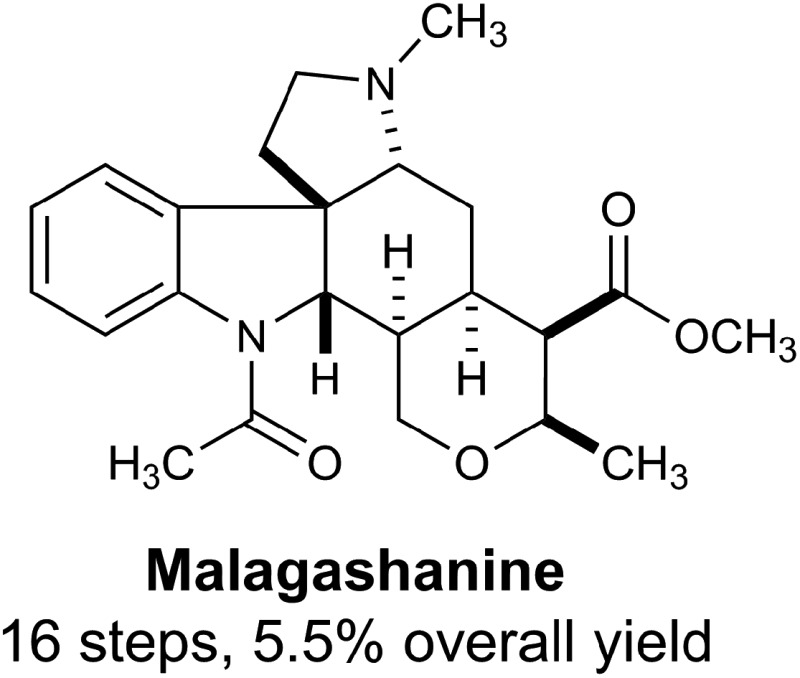
A stereoselective cascade annulation reaction generates the tetracyclic core of the Malagasy alkaloids, facilitating the total synthesis of malagashanine.

## Introduction

**Fig. 1:**
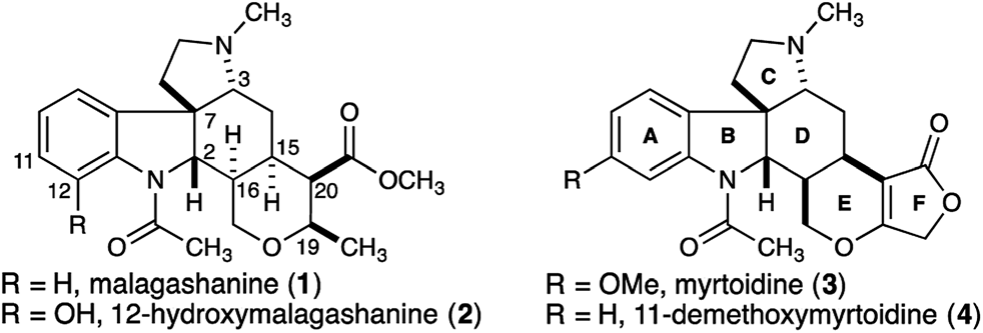
The Malagasy alkaloids.

Malagashanine (**1**, Fig. 1) is a structurally unusual alkaloid that was isolated from the stem bark of the Madagascan shrub *Strychnos myrtoides* in the early 1990s.^1^ It was isolated during an ethnobotanical study investigating local approaches to malaria treatment, and was found to potentiate chloroquine against otherwise resistant *Plasmodium falciparum*.^2^ To date, the mechanism of action for this observed activity has not been established, although it was noted that malagashanine impacts chloroquine accumulation in the food vacuole of the malaria parasite.^2*c*^ Although initially incorrectly assigned, the structure of malagashanine was unambiguously determined by X-ray crystallography.^3^ The pentacyclic alkaloid contains seven consecutive stereocenters, and most strikingly, a *trans*-ring fusion between the C and D rings. To the best of our knowledge, this represents the first report of a *trans*-ring fusion in a *Strychnos* alkaloid and as a result, this core structure had not been the focus of any synthetic studies. Subsequent to the discovery of malagashanine, several other alkaloids (**2–4**) sharing this unusual stereochemical arrangement have been identified from *Strychnos* species in both Africa and South America.^4^ These unprecedented structural features, combined with the potential utility of these natural products to further our understanding of malarial drug resistance highlight the Malagasy alkaloids as important targets to be addressed by total synthesis.

Prior to our studies, attempts to synthesize malagashanine by manipulation of strychnobrasiline had failed, as the *trans*-CD ring fusion could not be established.^4^ More recently, a synthesis of 16-*epi*-11-demethoxymyrtoidine was reported.^5^ These studies highlight the challenge presented by the seven consecutive stereocenters found in the natural product.

## Results and discussion

**Fig. 2:**
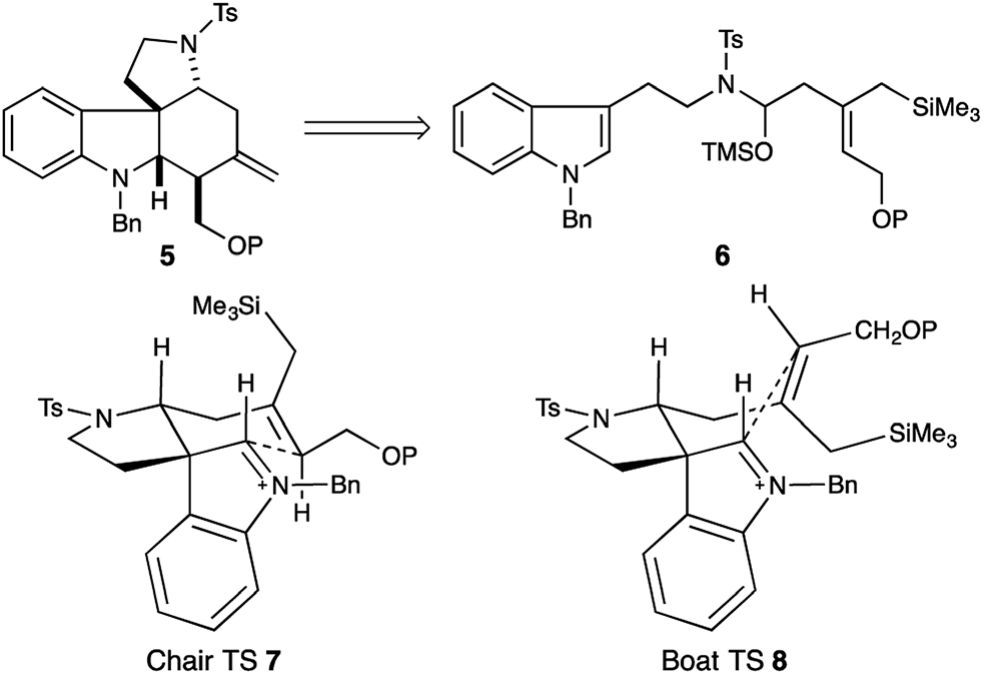
Synthetic strategy for malagashanine.

Strategically we chose to construct the tetracyclic core of the Malagasy alkaloids (**5**, Fig. 2) in a cascade annulation reaction, initiated by Lewis-acid induced decomposition of a simple linear aminol substrate (**6**) with subsequent indole attack on the resulting iminium ion, and finally, an appropriately positioned allylsilane would complete the sequence and close the D-ring of the natural product. This approach was inspired by Corey's elegant synthesis of aspidophytine.^6^ However, implementation in the context of the Malagasy alkaloid framework posed a number of significant challenges not addressed in the cascade established for aspidophytine, and thus offered an opportunity to further explore and define the generality of the strategy. Specifically, we would need to establish iminium ion geometry in an acyclic context, and translate this geometry into the correct C3–C7 relative stereochemistry required for malagashanine. Attack of the indole onto the iminium ion would need to outpace migration of the β,γ-unsaturation into conjugation, and a trisubstituted allylsilane would need to close stereoselectively onto the resulting indolium ion, without the help of a Thorpe–Ingold preorganization, prior to alkyl migration leading to competing Pictet–Spengler product.

**Scheme 1:**
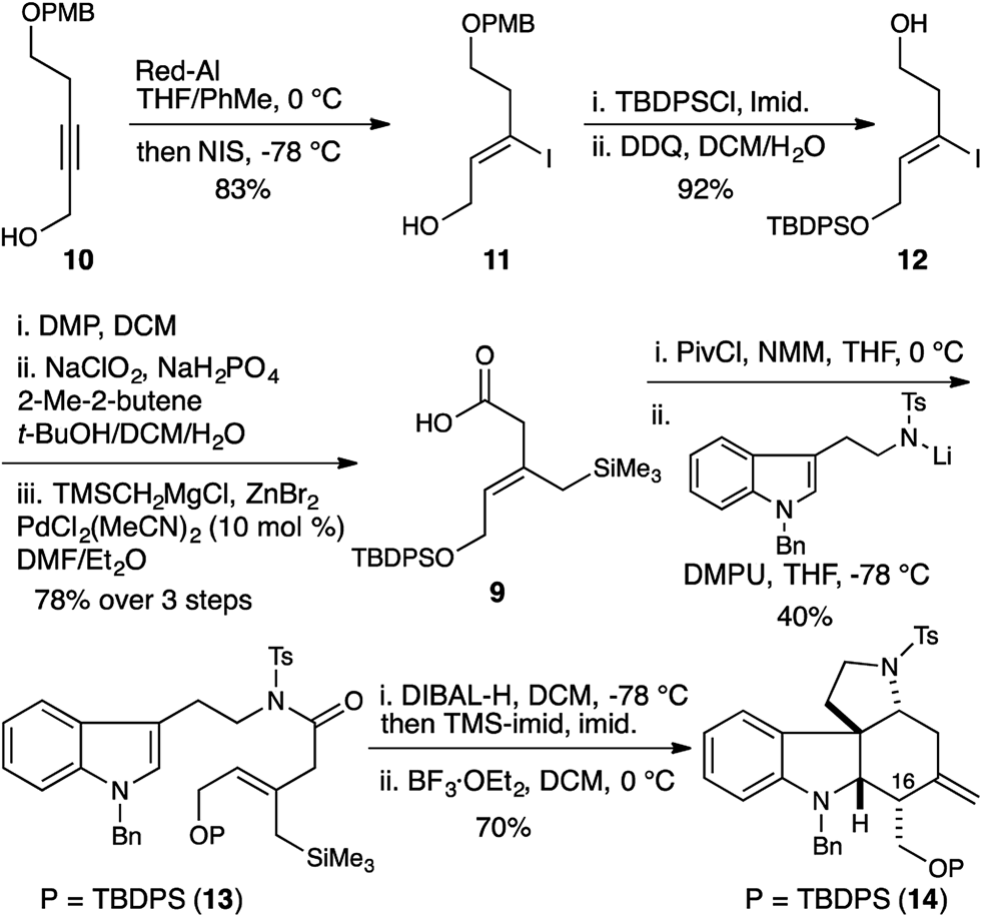
Synthesis and cyclization of *Z*-allylsilane precursor **13** gives the 16-*epi*-malagashanine core.

In a preliminary study we had established the importance of utilizing an *N*-tosyl iminium ion to selectively form the tetracyclic cascade product in preference to the Pictet–Spengler product, and that iminium-ion geometry control produced the required *trans*-ring fusion.^7^ However the delicate balance of the reaction suggested that extension to trisubstituted allylsilanes might not be straightforward. We initiated our study with the assumption that closure of the allylsilane onto the indolium ion would proceed through a chair transition state (**7**), and thus embarked on a synthesis of the *Z*-allylsilane **9** (Scheme 1).

The olefin geometry was established by Red-Al reduction of propargyl alcohol **10**.^8^ Quenching of the organoaluminate intermediate with NIS delivered the *Z*-vinyl iodide **11** in 83% yield. Alcohol protection and PMB removal provided alcohol **12** (92% yield). Subsequent oxidation to the carboxylic acid,^9^ and Negishi cross coupling delivered the required allylsilane **9** in 78% yield.^10^

**Scheme 2:**
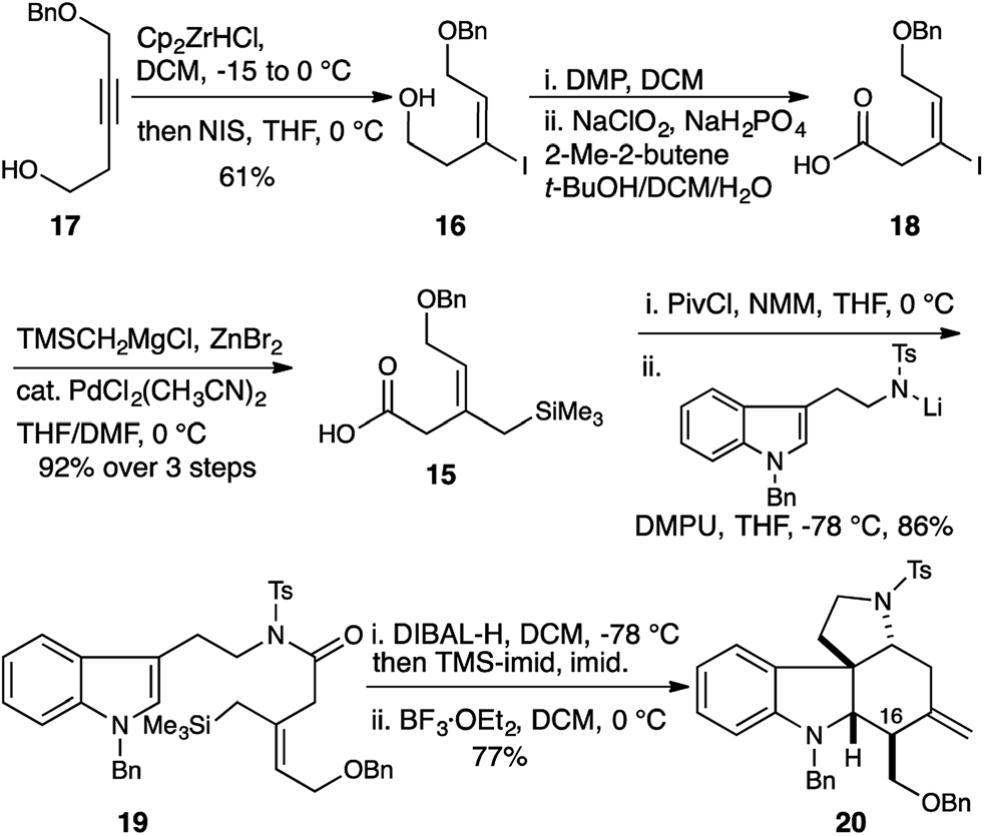
Allylsilane geometry controls C16 stereochemistry. Selective synthesis of the malagashanine tetracyclic core **20**.

*N*-Tosyl amidation was accomplished *via* the intermediacy of a mixed pivaloyl anhydride of acid **9**. In a modification of Kim's procedure,^11^ amide **13** was converted to the *N*-tosyl-*O*-TMS-aminol by DIBAL-H reduction and TMS-imidazole quench. We note that the addition of 20 mol% imidazole to the reaction accelerates the transmetallation from the aluminate to the silylether, and led to reproducibly high yields in the reaction. Finally, treatment of the aminol with BF_3_·OEt_2_ resulted in cascade cyclization to produce tetracycle **14** as a single diastereomer (70% yield). However extensive COSY and nOe NMR experiments revealed that the C16 stereocenter had the opposite relative configuration to that which is required for the synthesis of malagashanine, suggesting that the final cyclization had in fact proceeded through a boat transition state (**8**, Fig. 2). This analysis suggested that the *E*-olefin **15** would be required to complete the synthesis (Scheme 2).

This was accomplished in a straightforward manner, with Ready's directed hydrozirconation methodology delivering vinyl iodide **16** as a single geometric isomer from homopropargyl alcohol **17** in 61% yield.^12^ An additional 12% of iodide regioisomer was also isolated. Elaboration of alcohol **16** to carboxylic acid **18** and subsequent conversion to amide **19** proceeded smoothly using the reaction conditions established for the *Z*-isomer. Reduction to give the TMS-aminol and cyclization with BF_3_·OEt_2_ gave tetracycle **20** containing four of the seven stereocenters as a single diastereomer in excellent yield (77% over 2 steps). This high fidelity in translating allylsilane geometry into the C16 stereochemistry is consistent with a highly ordered boat transition state. The preference for boat transition state **8** over the chair **7** appears to be dictated by the spirocyclic motif linking the indolium ion and allylsilane. This motif splays the reactive terminals apart in a chair conformation, but allows good overlap between the allysilane π-system and the indolium ion π* orbital in the boat conformation.

**Scheme 3:**
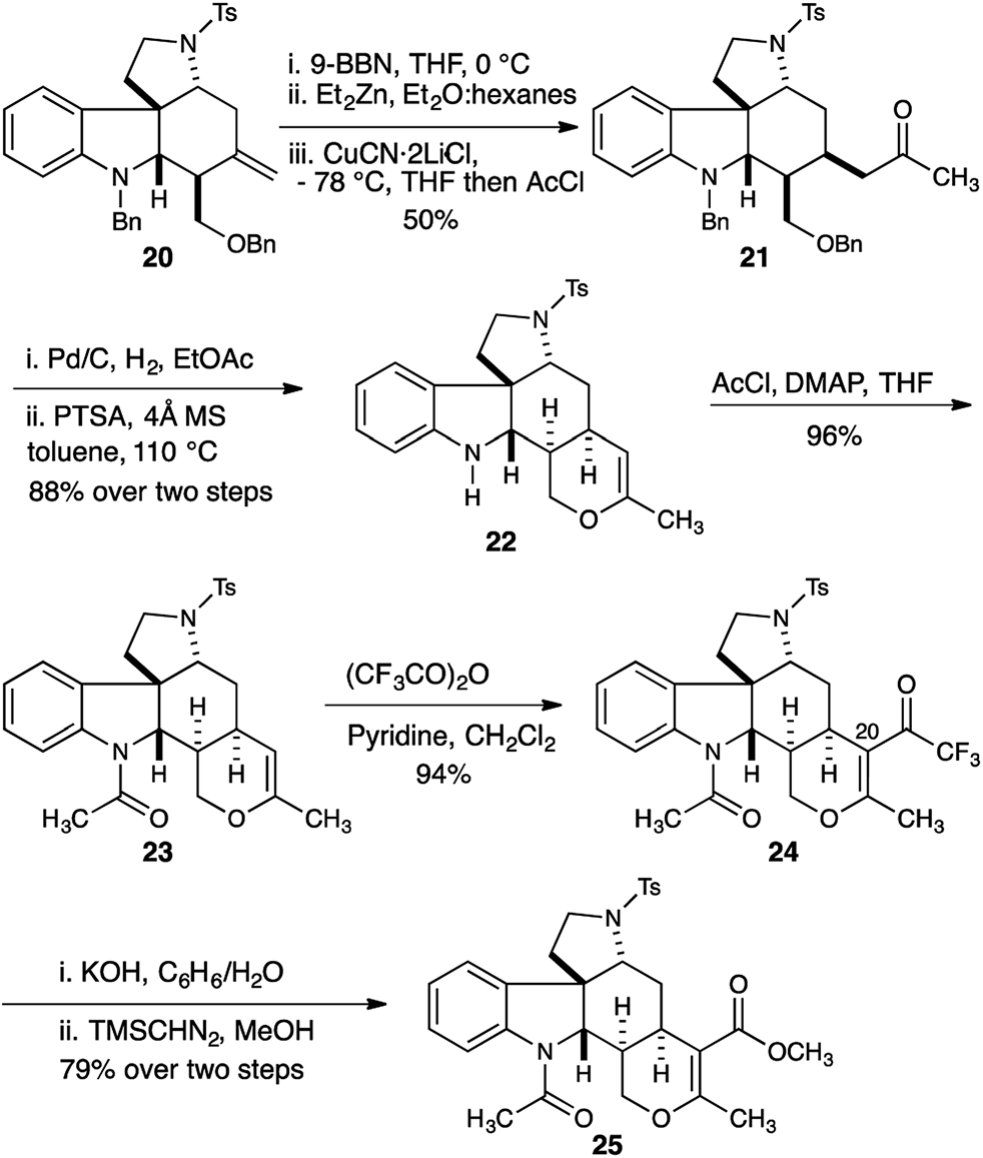
Core homologation and installation of the malagashanine E-ring.

Having established a robust synthesis of the core structure of the Malagasy alkaloids, our attention turned to installation of the tetrahydropyran E-ring. A hydroboration-Knochel transmetallation protocol enabled a formal hydroacylation of the exocyclic olefin of **20** (Scheme 3).^13^

This protocol was moderately selective providing a 4 : 1 mixture of separable diastereomers with the major diastereomer **21** isolated in 50% yield. Hydrogenolysis efficiently removed the benzyl protecting groups from both the indoline nitrogen and the 1° alcohol, and the crude reaction mixture was immediately subjected to acid catalyzed dehydration to deliver dihydropyran **22** in 88% yield. Installation of the acetamide found in the natural product proceeded smoothly (**23**, 96% yield). Introduction of the C20 ester functionality proved difficult, and acylation of the enol ether with trichloroacetic anhydride under a range of conditions delivered disappointing yields of the trichloroketone. Ultimately we found that acylation with the more reactive trifluoroacetic anhydride in the presence of pyridine was more effective, and gave reproducibly excellent yields (94%) of trifluoroketone **24**.^14^ Hydrolysis of the trifluoroketone was accomplished with potassium hydroxide in a refluxing water/benzene mixture, and treatment of the resulting carboxylic acid with TMS-diazomethane gave the methyl ester **25** in 79% yield (over 2 steps).

**Scheme 4:**
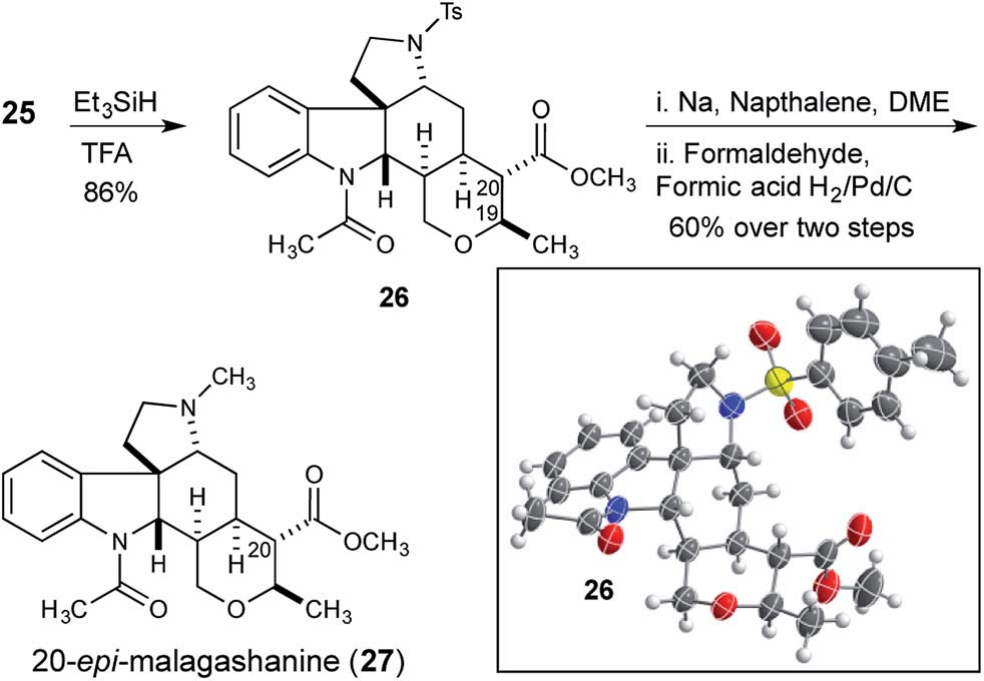
Ionic reduction of dihydropyran **25** leads to 20-*epi*-malagashanine.

At this stage we attempted to hydrogenate the tetrasubstituted olefin from the convex face, to complete the synthesis of the tetrahydropyran and set the remaining two stereocenters in the natural product. Although reductions of tetrasubstituted olefins are relatively rare, good results have been reported with a Rh/Josiphos combination,^15^ and with Pfaltz's iridium complexes.^16^ Unfortunately application of these conditions to olefin **25** did not result in any hydrogenation reaction. Additionally, a selection of common heterogeneous catalysts were also investigated under a variety of forcing conditions, but none were effective.^17^ Ionic reduction with triethylsilane in trifluoroacetic acid gave the reduced compound **26** (Scheme 4).^18^ However, the *trans*-reduction product, in which both the C20 methyl ester and the C19 methyl substituent of the tetrahydropyran occupy equatorial positions, was obtained.

The stereochemical outcome of the reaction was unambiguously confirmed by X-ray crystallography, and although the product was epimeric to the natural product at C20, this structure served to confirm that the previously established stereocenters were correctly configured. Reduction product **26** was converted to 20-*epi*-malagashanine (**27**) utilizing standard conditions (2 steps, 60% yield).

**Scheme 5:**
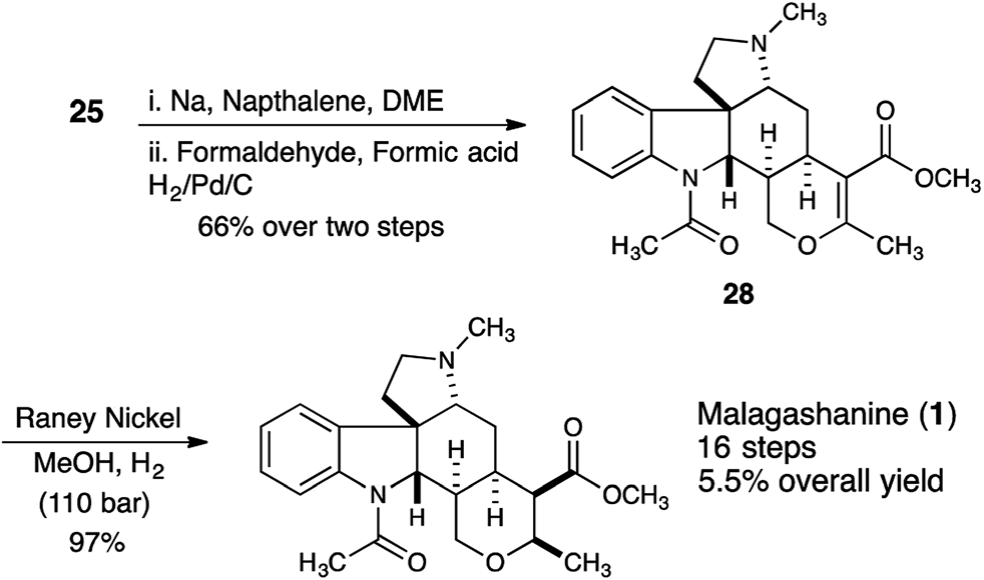
Completion of the total synthesis of malagashanine.

In order to synthesize the correctly configured natural product, we discovered that removal of the tosyl protecting group of **25** under dissolving metal conditions and reductive amination of the resulting amine provided substrate **28** that could be successfully hydrogenated using Raney nickel as the catalyst (110 bar, 5 days) providing malagashanine (**1**) in 97% yield (Scheme 5).

## Conclusions

In summary, we report a stereoselective synthesis of the chloroquine potentiating natural product malagashanine. A novel cascade annulation protocol efficiently constructs the C and D rings and installs four of the five consecutive D-ring stereocenters, including the critical *trans*-CD ring fusion. This represents the first total synthesis of a member of the Malagasy alkaloid family of natural products and provides a foundation for an exploration of the interesting biological activity presented by these compounds.
