# Supplementary material for: Total synthesis of malagashanine: a chloroquine potentiating indole alkaloid with unusual stereochemistry
Source: Chem Sci. 2016 Sep 19;8(1):697–700. doi: 10.1039/c6sc03578g (PMC5297967; doi:10.1039/c6sc03578g)

Electronic Supplementary Material (ESI) for Chemical Science.
This journal is © The Royal Society of Chemistry 2016

Supporting Information

Total Synthesis of Malagashanine: A Chloroquine Potentiating Indole Alkaloid with Unusual Stereochemistry.

Aidi Kong, Danny E. Mancheno, Nadège Boudet, Ricardo Delgado, Eric Andreansky and Simon B. Blakey*

Department of Chemistry, Emory University, Atlanta, GA 30322, U.S.A.

Contents

I.	General Information	S1
II.	Procedures and Characterization	S2
III.	Structural assignments for **14** and **26**	S25
IV.	Attempted reductions of **28**	S28
V.	NMR Spectra	S30

I. General Information

^{1}H and ^{13}C NMR spectra were recorded on a Varian Inova 600 spectrometer (600 MHz ^{1}H, 150 MHz ^{13}C), a Varian Unity plus 600 spectrometer (600 MHz ^{1}H, 150 MHz ^{13}C), and a Varian Inova 400 spectrometer (400 MHz ^{1}H, 100 MHz ^{13}C) at room temperature in $CDCl_3$ (neutralized and dried using anhydrous K_2CO_3) with internal $CHCl_3$ as the reference (7.26 ppm for ^{1}H and 77.23 ppm for ^{13}C), unless otherwise stated. Chemical shifts (δ values) were reported in parts per million (ppm) and coupling constants (*J* values) in Hz. Multiplicity was indicated using the following abbreviations: s = singlet, d = doublet, t = triplet, q = quartet, qn = quintet, m = multiplet, b = broad. Infrared (IR) spectra were recorded using Thermo Electron Corporation Nicolet 380 FT-IR spectrometer. High-resolution mass spectra were obtained using a Thermo Electron Corporation Finigan LTQFTMS (at the Mass Spectrometry Facility, Emory University). Melting points were taken using a Fisher Johns melting point apparatus and are uncorrected. Analytical thin layer chromatography (TLC) was performed on precoated glass backed EMD 0.25 mm silica gel 60 plates. Visualization was accomplished with UV light, ethanolic anisaldehyde, or $KMnO_4$. Flash column chromatography was carried out using Silicycle SilaFlash® F60 silica gel (40-63 µm). All reactions were conducted using anhydrous solvents in oven dried and nitrogen charged glassware. Anhydrous solvents were obtained by passage through activated alumina using a *Glass Contours* solvent purification system unless otherwise noted. Solvents used in workup, extraction and column chromatography were used as received from commercial suppliers without further purification. All reagents were purchased from Sigma Aldrich or Strem and used as received unless otherwise noted. 4Å powdered molecular sieves were activated by heating to 100 °C under reduced pressure (0.2 torr) for at least 12 hours. We acknowledge the use of shared instrumentation provided by grants from the NIH and the NSF.

II. Procedures and Characterization

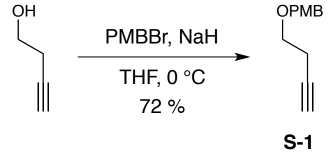

PMB Ether S-1:

4-methoxybenzyl alcohol (10.3 mL, 83.0 mmol, 1.0 equiv.) was added drop-wise to 48 % HBr (50.0 ml), and the mixture was stirred for one hour. Et_2O (100.0 mL) was then added, and the two layers were separated. The organic layer was washed with saturated aqueous $NaHCO_3$ (2 x 100.0 mL), dried over anhydrous $CaCl_2$ for thirty minutes, filtered, and concentrated under reduced pressure to provide crude 4-methoxybenzyl bromide, which was used immediately without further purification.

A slurry of sodium hydride (60 wt % dispersion in mineral oil, 5.27 g, 132 mmol, 1.6 equiv.) in THF (50.0 mL) was cooled to 0 °C. 3-Butyn-1-ol (7.6 mL, 100.0 mmol, 1.2 equiv.) was added drop-wise over 15 minutes, and the reaction was stirred for one hour. Crude 4-methoxybenzyl bromide was then added, and the reaction mixture was warmed up to room temperature and stirred for 14 hours. The reaction was quenched with saturated aqueous NH_4Cl (200.0 mL). The layers were separated, and the aqueous phase was extracted with Et_2O (3 x 100.0 mL). The combined organic layer was washed with brine (100.0 mL), dried over anhydrous $MgSO_4$, filtered, and concentrated under reduced pressure. Purification by flash column chromatography on silica gel (19:1 pentane/Et_2O) provided the PMB ether **S-1** (11.4 g, 72 %) as a colorless oil.

R_f 0.63 (7:3 hexanes/EtOAc); **^{1}H NMR** (500 MHz, $CDCl_3$) δ 7.29 (d, J = 8.3 Hz, 2H), 6.90 (d, J = 8.7 Hz, 2H), 4.51 (s, 2H), 3.83 (s, 3H) 3.59 (t, J = 7.0 Hz, 2H), 2.51 (td, J = 7.0, 2.7 Hz, 2H), 2.01 (t, J = 2.7 Hz, 1H) ppm; **^{13}C NMR** (126 MHz, $CDCl_3$) δ 159.4, 130.2, 129.5, 113.9, 81.5, 72.8, 69.4, 67.9, 55.4, 20.0 ppm; **IR** (thin film, cm^{-1}) 3289, 2860, 1612, 1586, 1511, 1463, 1361, 1301, 1243, 1173, 1092, 1032, 819, 756, 636, 581; **HRMS** (+APCI) calculated for $C_{12}H_{14}O_2$ $[M]^+$ 190.0994, found 190.0991.

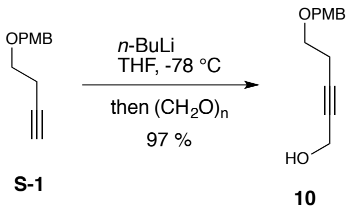

Propargyl Alcohol 10:

A solution of PMB ether **S-1** (11.4 g, 59.9 mmol, 1.0 equiv.) in THF (80.0 mL) was cooled to -78 °C. *n*-BuLi (2.5 M in hexanes, 26.4 mL, 65.9 mmol, 1.1 equiv.) was added drop-wise over five minutes, and the resultant mixture was stirred for one hour. Paraformaldehyde (5.40 g, 179.4 mmol of monomer, 3.0 equiv.) was added in one portion, and the reaction was warmed to room temperature and stirred for 12 hours. The mixture was quenched with saturated aqueous NH_4Cl (50.0 mL) and water (50.0 mL).

Et_2O (100.0 mL) was added, and the layers were separated. The aqueous phase was extracted with Et_2O (3 x 100.0 mL). The combined organic layer was washed with brine (100.0 mL), dried over anhydrous $MgSO_4$, filtered, and concentrated under reduced pressure. Purification by flash column chromatography on silica gel (2:1 hexanes/EtOAc) provided propargyl alcohol **10** (12.8 g, 97 %) as a colorless oil.
R_f 0.20 (7:3 hexanes/EtOAc); **^{1}H NMR** (500 MHz, $CDCl_3$) δ 7.29 (d, J = 8.7 Hz, 2H), 6.90 (d, J = 8.7 Hz, 2H), 4.50 (s, 2H), 4.25 (s, 2H), 3.82 (s, 3H), 3.57 (t, J = 6.9 Hz, 2H), 2.53 (tt, J = 6.9, 2.2 Hz, 2H) ppm; **^{13}C NMR** (126 MHz, $CDCl_3$) δ 159.4, 130.1, 129.5, 113.9, 83.2, 79.6, 72.7, 68.0, 55.4, 51.4, 20.3 ppm; **IR** (thin film, cm^{-1}) 3405, 2910, 2863, 1612, 1586, 1512, 1462, 1361, 1302, 1244, 1174, 1135, 1089, 1026, 819, 755, 710, 637, 579; **HRMS** (+NSI) calculated for $C_{13}H_{20}O_3N$ $[M+NH_4]^+$ 238.1438, found 238.1439.

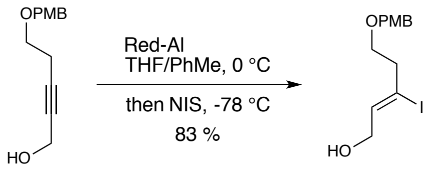

10 11

Vinyl Iodide 11:

Propargyl alcohol **10** (10.5 g, 47.7 mmol, 1.0 equiv.) was dissolved in THF (50.0 mL) and cooled to 0 °C. Red-Al® (60 % wt/v solution in toluene, 27.3 mL, 81.0 mmol, 1.7 equiv.) was added drop-wise over ten minutes. The resulting slurry was warmed to room temperature and stirred for three hours. The reaction mixture was cooled to -78 °C, and a solution of *N*-iodosuccinimide (19.3 g, 85.9 mmol, 1.8 equiv.) in THF (50.0 mL) was added via cannula. The reaction mixture was stirred for 30 minutes. Upon warming to room temperature, the reaction mixture was quickly poured into a 1:1 mixture of saturated aqueous Na_2SO_3/saturated Rochelle's salt solution (300.0 mL), and the biphasic mixture was stirred vigorously until both layers were clear and colorless. The layers were separated, and the aqueous phase was extracted with Et_2O (3 x 100.0 mL). The combined organic layer was washed with brine (100.0 mL), dried over anhydrous $MgSO_4$, filtered, and concentrated under reduced pressure. Purification by flash column chromatography on silica gel (3:1 to 2:1 hexanes/EtOAc) provided vinyl iodide **11** (13.7 g, 83 %) as a pale yellow oil.
R_f 0.35 (7:3 hexanes/EtOAc); **^{1}H NMR** (500 MHz, $CDCl_3$) δ 7.27 (d, J = 8.7 Hz, 2H), 6.89 (d, J = 8.7 Hz, 2H), 5.91 (tt, J = 5.7, 1.1 Hz, 1H), 4.46 (s, 2H), 4.14 (d, J = 5.6 Hz, 2H), 3.80 (s, 3H), 3.60 (t, J = 6.4 Hz, 2H), 2.77 (td, J = 6.4, 1.0 Hz, 2H) ppm; **^{13}C NMR** (126 MHz, $CDCl_3$) δ 159.2, 136.1, 130.0, 129.4, 113.8, 104.5, 72.7, 68.3, 67.2, 55.3, 45.1 ppm; **IR** (thin film, cm^{-1}) 3370, 2859, 1611, 1511, 1460, 1360, 1301, 1244, 1173, 1081, 1030, 814, 755, 566; **HRMS** (+NSI): calculated for $C_{13}H_{21}O_3NI$ $[M+NH_4]^+$ 366.0561, found 366.0563.

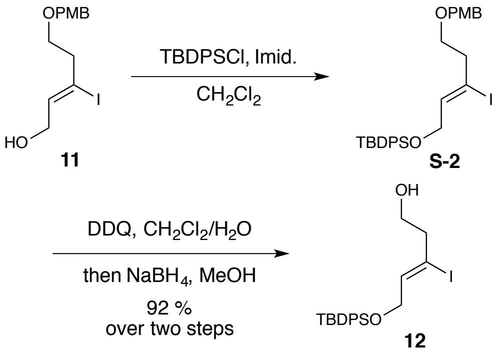

Alcohol 12:

Vinyl iodide **11** (8.57 g, 24.7 mmol, 1.0 equiv.) and imidazole (3.36 g, 49.3 mmol, 2.0 equiv.) were dissolved in CH_2Cl_2 (40.0 mL) and cooled to 0 °C. *Tert*-butyl(chloro)diphenylsilane (7.05 mL, 27.1 mmol, 1.1 equiv.) was added drop-wise, and the reaction mixture was stirred at room temperature for three hours. The reaction was quenched with saturated aqueous NH_4Cl (60.0 mL), and the layers were separated. The aqueous phase was extracted with CH_2Cl_2 (3 x 50.0 mL). The combined organic layer was washed with water (100.0 mL) and brine (100.0 mL). The organic layer was dried over anhydrous Na_2SO_4, filtered, and concentrated under reduced pressure to provide the crude TBDPS ether **S-2**, which was used directly in the next step without further purification.

Crude TBDPS ether **S-2** was dissolved in CH_2Cl_2 (40.0 mL) and cooled to 0 °C. DDQ (6.15 g, 27.1 mmol, 1.1 equiv) and water (2.0 mL) were added, and the resultant black slurry was stirred for one hour. The reaction mixture was filtered over celite, and the filtrate quenched with of a 1:1 mixture of saturated aqueous Na_2SO_3/saturated aqueous $NaHCO_3$ (150.0 mL). The layers were separated, and the aqueous phase was extracted with DCM (3 x 100.0 mL). The combined organic layer was washed with brine (100.0 mL), dried over anhydrous Na_2SO_4, filtered, and concentrated under reduced pressure. The crude mixture was brought up in dry MeOH (40.0 mL) and cooled to 0 °C. $NaBH_4$ (2.80 g, 74.1 mmol, 3.0 equiv.) was added in portions, and the mixture was stirred for four hours. The solution was concentrated under reduced pressure, and the residue was brought up in Et_2O (100.0 mL) and H_2O (100.0 ml). The layers were separated, and the aqueous phase was extracted with Et_2O (3 x 100.0 ml). The combined organic layer was washed with brine (100.0 mL), dried over anhydrous $MgSO_4$, filtered, and concentrated under reduced pressure. Purification by flash chromatography on silica gel (4:1 to 7:3 hexanes/EtOAc) provided alcohol **12** (10.52 g, 92 % over three steps) as a colorless oil.

R_f 0.62 (7:3 hexanes/EtOAc) **^{1}H NMR** (500 MHz, $CDCl_3$) δ 7.72 – 7.64 (m, 4H), 7.48 – 7.37 (m, 6H), 6.01 (tt, J = 5.1, 1.1 Hz, 1H), 4.28 (dt, J = 5.0, 1.0 Hz, 2H), 3.71 (t, J = 5.8 Hz, 2H), 2.68 (tq, J = 6.0, 1.0 Hz, 2H), 1.07 (s, 9H) ppm; **^{13}C NMR** (126 MHz, $CDCl_3$) δ 138.0, 135.7, 133.5, 129.9, 127.9, 102.2, 69.1, 61.0, 47.9, 26.9, 19.3 ppm; **IR** (thin film, cm^{-1}) 3392, 3071, 2931, 2858, 1472, 1427, 1264, 1110, 1051, 907, 823, 731, 701, 612; **HRMS** (+NSI): calculated for $C_{21}H_{27}O_2INaSi$ $[M+Na]^+$ 489.0717, found 489.0718.

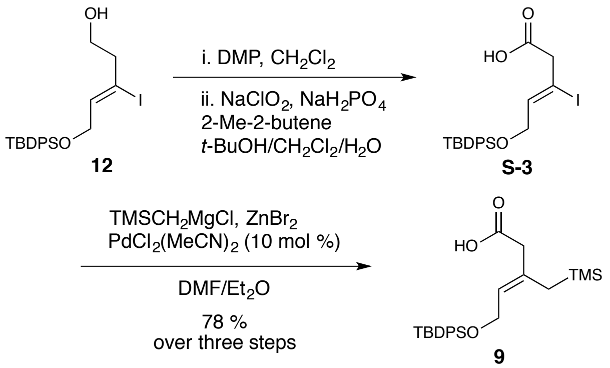

Allylsilane 9:

Alcohol **12** (10.52 g, 22.5 mmol, 1.0 equiv.) was brought up in CH_2Cl_2 (50.0 mL). Dess-Martin Periodinane (11.48 g, 27.1 mmol, 1.2 equiv.) was then added, and the resultant slurry was stirred at room temperature for three hours. The reaction was diluted with EtOAc (100.0 mL) and quenched with a 1:1 mixture of saturated aqueous Na_2SO_3/saturated aqueous $NaHCO_3$ (100.0 mL). The layers were separated, and the aqueous phase was extracted with EtOAc (3 x 50.0 mL). The combined organic layer was washed with brine (100.0 mL), dried over anhydrous Na_2SO_4, filtered, and concentrated under reduced pressure. The crude residue was brought up in *t*-BuOH (100.0 mL) and CH_2Cl_2 (100.0 mL). 2-methyl-2-butene (50.0 mL) was then added, and the reaction was cooled to 0 °C. $NaClO_2$ (20.3 g, 225.0 mmol, 10.0 equiv.) and NaH_2PO_4 (21.6 g, 180 mmol, 8.0 equiv.) were dissolved in H_2O (100.0 mL), and the resultant solution was added in one portion to the reaction mixture. The biphasic mixture was stirred for one hour and then diluted with water (150.0 mL). CH_2Cl_2 (150.0 mL) was added, and the layers were separated. The organic layer was washed with water (100.0 mL) and brine (100.0 mL), dried over anhydrous Na_2SO_4, filtered, and concentrated under reduced pressure. Residual *t*-BuOH was removed by azeotroping the residue with toluene (3 x 50.0 mL) to provide the crude carboxylic acid **S-3** as a yellow oil, which was used without further purification.

(Chloromethyl)trimethylsilane (9.4 mL, 67.5 mmol, 3.0 equiv.) was dissolved in dry Et_2O (68.0 mL). Mg turnings (1.68 g, 67.5 mmol, 3.0 equiv) and a crystal of iodine were added. An initial exotherm was observed, and the mixture was stirred for five hours at reflux until all magnesium was consumed. The freshly formed Grignard solution was cooled to room temperature and added via cannula to anhydrous $ZnBr_2$ (15.80 g, 69.8 mmol, 3.1 equiv), and the resultant slurry was stirred for 12 hours. DMF (68.0 mL) was then added. A solution of crude carboxylic acid **S-3** in DMF (15.0 mL) was added, and the reaction mixture was cooled to 0 °C. A solution of $Pd(CH_3CN)_2Cl_2$ (583.7 mg, 2.25 mmol, 0.1 equiv.) in DMF (10.0 mL) was then added, and the reaction was warmed to room temperature and stirred for three hours. The reaction was quenched with saturated aqueous NH_4Cl (50.0 mL) and Et_2O (100.0 mL), and the layers were separated. The aqueous phase was extracted with Et_2O (3 x 100.0 mL). The combined organic layer was washed with H_2O (2 X 150.0 mL), 5 % aqueous LiCl (2 x 150.0 ml), and brine (100.0 mL). The organic layer was dried over anhydrous $MgSO_4$, filtered through celite, and concentrated under reduced pressure. Purification by flash column chromatography on

silica gel (9:1 hexanes/EtOAc) provided allyl silane **9** (7.72 g, 78 % over three steps) as a yellow oil.

$\mathbf{R_f}$ 0.60 (7:3 hexanes/EtOAc); **^{1}H NMR** (500 MHz, $CDCl_3$) δ 7.73 – 7.67 (m, 4H), 7.45 – 7.36 (m, 6H), 5.49 (t, J = 6.1 Hz, 1H), 4.17 (d, J = 6.1 Hz, 2H), 2.98 (s, 2H), 1.47 (s, 2H), 1.06 (s, 9H), -0.04 (s, 9H) ppm; **^{13}C NMR** (126 MHz, $CDCl_3$) δ 178.0, 135.7, 134.0, 131.5, 129.7, 126.6, 61.3, 44.6, 27.0, 22.1, 19.3, -0.9 ppm; **IR** (thin film, cm^{-1}) 3071, 2954, 2856, 1708, 1427, 1249, 1111, 1079, 1046, 850, 824, 736, 701, 613; **HRMS** (+NSI) calculated for $C_{25}H_{37}O_3Si_2$ $[M+H]^+$ 441.2276, found 441.2273.

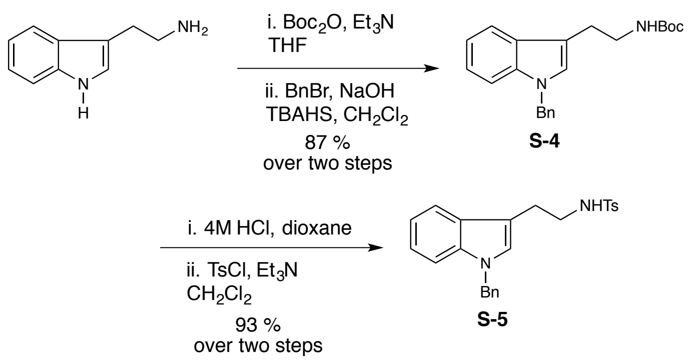

Sulfonamide S-5:

Carbamate **S-4** was prepared according to modified version of a previously reported procedure. Et_3N (6.3 mL, 45.0 mmol, 1.5 equiv.) was added to a solution of tryptamine (4.8 g, 30.0 mmol, 1.0 equiv.) in THF (150.0 mL) at 0 °C. A solution of Boc_2O (7.2 g, 33.0 mmol, 1.1 equiv.) in THF (50.0 mL) was added over 15 minutes via cannula. The mixture was gradually warmed to room temperature and stirred overnight. The reaction mixture was concentrated under reduced pressure, and the residue was filtered through a pad of silica gel (eluted with 1:1 hexanes/EtOAc). The filtrate was concentrated, and the resultant residue was used in the next step without further purification. A mixture of the crude residue, powdered NaOH (3.0 g, 75.0 mmol, 2.5 equiv.) and Bu_4NHSO_4 (1.0 g, 3.0 mmol, 0.1 equiv.) in CH_2Cl_2 (200.0 mL) was cooled to 0 °C. BnBr (5.6 g, 30.0 mmol, 1.0 equiv.) was slowly added. The mixture was gradually warmed to room temperature and stirred overnight. The reaction was quenched with water (40.0 mL), and the layers were separated. The aqueous phase was extracted with CH_2Cl_2 (2 x 50.0 mL). The combined organic extracts were washed with brine (2 x 30.0 mL), dried over anhydrous Na_2SO_4, filtered, and concentrated under reduced pressure. Purification by flash column chromatography on silica gel (10:1 to 4:1 hexanes/EtOAc) provided carbamate **S-4** (9.5 g, 87 % over two steps) as a colorless oil. Spectral data matched that previously reported in the literature. $\mathbf{R_f}$ 0.80(hexanes/EtOAc, 2:1).

A solution of carbamate **S-4** (1.8 g, 5.0 mmol, 1.0 equiv.) in CH_2Cl_2 (26.0 mL) was cooled to 0 °C. A solution of HCl in dioxane (4.0 M, 26.0 mL) was slowly added. The reaction mixture was warmed up to room temperature and stirred for 1.5 hours. The reaction was concentrated under reduced pressure, and the residue was brought up in CH_2Cl_2 (29.0 mL) and cooled to 0 °C. Et_3N (2.9 mL, 4.0 equiv.) was added, followed by a solution of TsCl (1.0 g, 1.05 equiv.) in CH_2Cl_2 (15.0 mL). The mixture was warmed to room temperature and stirred overnight. The reaction was quenched with water (20.0 mL), and the layers were separated. The aqueous phase was extracted with CH_2Cl_2 (2 x

30.0 mL). The combined organic layer was washed with brine (2 x 30.0 mL), dried over anhydrous Na_2SO_4, filtered, and concentrated under reduced pressure. Purification by flash column chromatography on silica gel (10:1 to 3:1 hexanes/EtOAc) afforded sulfonamide **S-5** (1.9 g, 93 % over two steps) as a colorless oil.
R_f 0.40 (hexanes/EtOAc, 2:1). **^{1}H NMR** ($CDCl_3$, 400 MHz) δ 7.63 (d, *J* = 8.4 Hz, 2H), 7.43 (d, *J* = 7.6 Hz, 1H), 7.34 – 7.26 (m, 4H), 7.21 (d, *J* = 8.0 Hz, 2H), 7.17 (dd, *J* = 8.0, 0.8 Hz, 1H), 7.13 – 7.11 (m, 2H), 7.06 (td, *J* = 8.0, 0.8 Hz, 1H), 6.87 (s, 1H), 5.26 (s, 2H), 4.40 (br s, 1H), 3.29 (t, *J* = 6.8 Hz, 2H), 2.94 (t, *J* = 6.8 Hz, 2H), 2.40 (s, 3H); **^{13}C NMR** ($CDCl_3$, 150 MHz) δ 148.5, 137.5, 137.0, 129.8, 129.0, 127.9, 127.8, 127.2, 127.0, 126.8, 122.3, 119.5, 119.0, 110.9, 110.1, 50.2, 43.3, 25.7, 21.7; **IR** (thin film, cm^{-1}) 3281, 1495, 1466, 1322, 1153, 1092, 813, 735, 660, 549; **HRMS** (+NSI) calculated for $C_{24}H_{25}N_2O_2S$ $[M+H]^+$ 405.1631, found 405.1623.

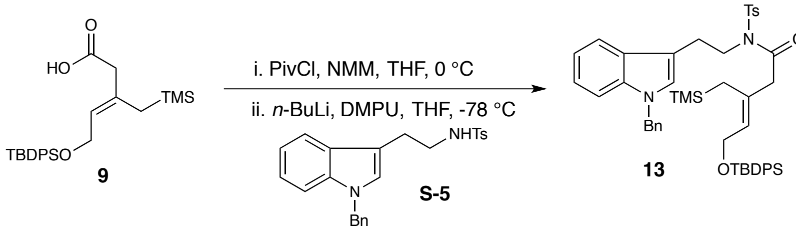

40 %

N-Tosylamide 13:

To a solution of carboxylic acid **9** (1.05 g, 2.38 mmol, 1.3 equiv.) in THF (23.8 mL) at 0 °C was added *N*-methylmorpholine (0.29 mL, 2.57 mmol, 1.4 equiv.). Pivaloyl chloride (0.29 mL, 2.38 mmol, 1.3 equiv.) was then added, and the resultant mixture was stirred for one hour. Stirring was discontinued, and the suspension was allowed to settle for one hour. The resulting supernatant was transferred into a separate flask. THF (23.8 ml) was added to the remaining white precipitate. The resulting white suspension was stirred for two minutes and then allowed to settle for one hour. The supernatant was combined with the rest of the mixed anhydride solution. In a separate flask, a solution of sulfonamide **S-5** (738.4 mg, 1.83 mmol, 1.0 equiv.) in THF (18.3 mL) was cooled to -78 °C, and DMPU (1.83 mL) was added. *n*-BuLi (4.4 M in hexanes, 0.50 mL, 2.20 mmol, 1.2 equiv.) was added drop-wise over five minutes, and the reaction was stirred for one hour. The mixed anhydride solution was added to the sulfonamide solution drop-wise over fifteen minutes, and the reaction was stirred at -78 °C for two hours. The reaction was quenched with saturated aqueous NH_4Cl (100.0 mL) and Et_2O (100.0 mL), and was warmed to room temperature. The layers were separated, and the aqueous phase was extracted with Et_2O (3 x 50.0 mL). The combined organic layer was washed with brine (100.0 mL), dried over anhydrous $MgSO_4$, filtered, and concentrated under reduced pressure. Purification by flash column chromatography on silica gel (19:1 to 9:1 hexanes/EtOAc) provided *N*-tosylamide **13** (610.0 mg, 40 %) as a sticky foam.
R_f 0.76 (7:3 hexanes/EtOAc); **^{1}H NMR** (500 MHz, $CDCl_3$) *δ* 7.84 (d, *J* = 8.3 Hz, 2H), 7.77 (d, *J* = 7.5 Hz, 1H), 7.68 – 7.64 (m, 4H), 7.45 – 7.34 (m, 6H), 7.32 – 7.23 (m, 6H), 7.21-7.09 (m, 4H), 7.00 (s, 1H), 5.26 (s, 2H), 5.13 (t, *J* = 5.8 Hz, 1H), 4.11 – 4.03 (m, 4H), 3.25 – 3.21 (m, 2H), 3.01 (s, 2H), 2.40 (s, 3H), 1.26 (s, 2H), 1.04 (s, 9H), -0.19 (s,

9H) ppm; **^{13}C NMR** (126 MHz, $CDCl_3$) δ 171.1, 144.8, 137.6, 137.0, 136.7, 135.6, 133.9, 132.1, 129.8, 129.7, 128.9, 128.0, 127.0, 125.9, 122.1, 119.5, 119.2, 111.3, 110.0, 61.2, 50.1, 48.1, 46.3, 27.0, 26.0, 22.4, 21.7, 19.3, -1.0 ppm; **IR** (thin film, cm^{-1}) 3049, 2930, 2857, 1693, 1467, 1352, 1161, 1110, 1087, 907, 849, 728, 701, 648, 611, 581, 543; **HRMS** (+NSI): calculated for $C_{49}H_{58}O_4N_2SSi_2$ $[M]^+$ 826.3650, found 826.3661.

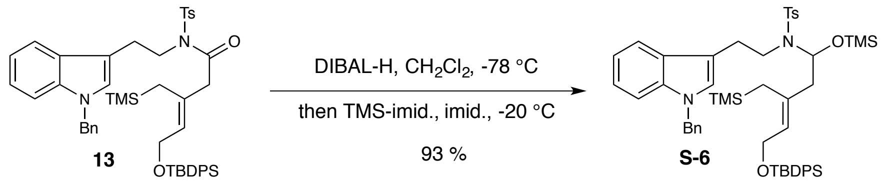

Hemiaminal S-6:

A solution of *N*-tosylamide **13** (510 mg, 0.62 mmol, 1.0 equiv.) in CH_2Cl_2 (2.1 mL) was cooled to 0 °C. DIBAL-H (1.0 M in DCM, 1.3 mL, 1.30 mmol, 2.10 equiv.) was added drop-wise over five minutes, and the resultant mixture was stirred at -78 °C for one hour. Trimethylsilylimidazole (.28 mL, 1.85 mmol, 3.0 equiv.) and imidazole (42 mg, 0.62 mmol, 1.0 equiv.) were added, and the reaction was stirred at -20 °C for 18 hours. The reaction was poured into a saturated Rochelle's salt solution (20.0 mL), and CH_2Cl_2 was added (20.0 mL). The biphasic mixture was vigorously stirred until both layers were clear. The layers were separated, and the aqueous phase was extracted with CH_2Cl_2 (3 x 20.0 mL). The combined organic layer was washed with brine (50.0 mL), dried over anhydrous Na_2SO_4, filtered, and concentrated under reduced pressure. Purification by flash column chromatography on silica gel (9:1 hexanes/EtOAc) to provided hemiaminal **S-6** (517.0 mg, 93 %) as a sticky foam.

R_f 0.79 (7:3 hexanes/EtOAc); **^{1}H NMR** (500 MHz, $CDCl_3$) δ 7.76 – 7.60 (m, 7H), 7.45 – 7.35 (m, 6H), 7.33 – 7.09 (m, 10H), 6.97 (s, 1H), 5.50 (dd, *J* = 8.5, 2.5 Hz, 1H), 5.33 – 5.26 (m, 3H), 4.08 (d, *J* = 6.1 Hz, 2H), 3.55 (td, *J* = 15.2, 5.2 Hz, 1H), 3.41 (td, *J* = 15.8, 4.8 Hz, 1H), 3.25 (td, *J* = 12.7, 4.7 Hz, 1H), 3.14 (td, *J* = 12.6, 4.9 Hz, 1H), 2.37 (s, 3H), 2.30 (dd, *J* = 12.9, 8.7 Hz, 1H), 1.96-1.89 (m, 1H), 1.49 (d, J = 13.5 Hz, 1H), 1.28 (d, J = 8.9 Hz, 1H), 1.05 (s, 9H), 0.15 (s, 9H), -0.11 (s, 9H) ppm; **^{13}C NMR** (126 MHz, $CDCl_3$) δ 143.1, 138.6, 137.7, 136.8, 135.7, 134.4, 134.0, 129.7, 128.9, 128.2, 127.8, 127.2, 127.0, 126.2, 125.3, 122.0, 119.4, 119.3, 112.9, 109.8, 82.0, 61.3, 50.0, 46.5, 44.0, 27.8, 27.0, 22.5, 21.6, 19.3, 0.3, -0.9 ppm; **IR** (thin film, cm^{-1}) 3069, 2954, 2857, 1598, 1467, 1333, 1250, 1160, 1106, 1083, 1054, 932, 844, 736, 701, 660, 614, 589, 550; **HRMS** (+NSI): calculated for $C_{52}H_{68}O_4N_2SSi_3$ $[M]^+$ 900.4202, found 900.4244.

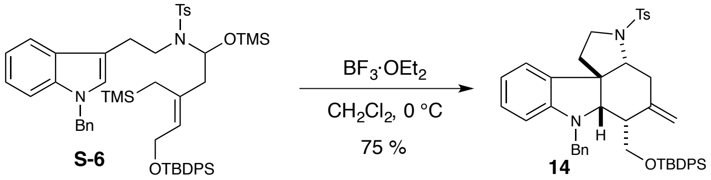

Tetracycle 14:

A solution of hemiaminal **S-6** (517.0 mg, 0.574 mmol, 1.0 equiv.) in CH_2Cl_2 (11.4 mL) was cooled to 0 °C. Freshly distilled BF_3·OEt_2 (0.22 mL, 1.72 mmol, 3.0 equiv.) was added drop-wise, and the solution was stirred at 0 °C for one hour. The reaction was quenched by the slow addition of saturated aqueous $NaHCO_3$ (20.0 mL) and was stirred for one hour. The layers were separated, and the aqueous phase was extracted with CH_2Cl_2 (3 x 20.0 mL). The combined organic layer was dried over anhydrous Na_2SO_4, filtered, and concentrated under reduced pressure. Purification by flash column chromatography on silica gel (4:1 hexanes/EtOAc) provided tetracycle **14** (318.3 mg, 75 %) as a sticky foam.

R_f 0.74 (7:3 hexanes/EtOAc); **^{1}H NMR** (500 MHz, $CDCl_3$) δ 7.75 (d, J = 8.2 Hz, 2H), 7.60 (t, J = 7.7 Hz, 4H), 7.48 - 7.30 (m, 9H), 7.21 – 7.17 (m, 3H), 7.14 – 7.10 (m, 2H), 7.02 (t, J = 7.7 Hz, 1H), 6.62 (t, J = 7.4 Hz, 1H), 6.43 (d, J = 7.9 Hz, 1H), 4.66 (d, J = 15.9 Hz, 1H), 4.54 (s, 1H), 4.42 (d, J = 15.9 Hz, 1H), 4.29 (s, 1H), 4.02 (t, J = 9.9 Hz, 1H), 3.87 (d, J = 4.9 Hz, 1H), 3.81 (dd, J = 10.2, 4.5 Hz, 1H), 3.62 (td, J = 10.4, 7.7 Hz, 1H), 3.43 - 3.32 (m, 2H), 2.94 (dd, J = 16.4, 5.8 Hz, 1H), 2.53- 2.34 (m, 5H), 1.44 (dd, J = 12.0, 7.4 Hz, 1H), 1.32 (q, J = 10.4 Hz, 1H), 1.07 (s, 9H) ppm; **^{13}C NMR** (126 MHz, $CDCl_3$) δ 151.9, 143.8, 141.5, 138.9, 135.7, 133.8, 133.6, 133.3, 132.1, 129.9, 128.5, 128.4, 128.0, 127.9, 127.7, 127.1, 125.1, 117.9, 110.9, 108.7, 66.6, 62.3, 59.3, 56.4, 52.8, 47.8, 45.2, 37.3, 34.8, 27.2, 21.8, 19.5 ppm; **IR** (thin film, cm^{-1}) 3030, 2928, 2856, 1599, 1484, 1391, 1350, 1162, 1106, 1057, 907, 818, 728, 700, 664, 649, 613, 582, 550; **HRMS** (+NSI): calculated for $C_{46}H_{50}O_3N_2SSi$ $[M]^+$ 738.3306, found 738.3309.

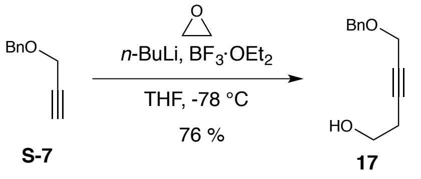

Homopropargylic Alcohol 17:

A solution of benzyl propargyl ether **S-7** (3.52 g, 24.1 mmol, 1.0 equiv.) in THF (150.0 mL) was cooled to -78 °C. *n*-BuLi (2.5 M in hexanes, 11.6 mL, 28.9 mmol, 1.2 equiv.) was slowly added over 20 minutes, and the resulting solution was stirred at -78 °C for one hour. Freshly distilled BF_3·OEt_2 (3.86 mL, 31.3 mmol, 1.3 equiv.) was added over ten minutes, and the solution was stirred for 15 minutes. During this time, oxirane (1.55 mL, 31.3 mmol, 1.3 equiv.) was condensed with a cold finger (-78 °C) and dissolved in THF (3.0 mL) at -78 °C. The oxirane solution was transferred into the reaction mixture via cannula, and the reaction mixture was stirred at -78 °C for two hours. The reaction

was quenched with saturated aqueous NH_4Cl (45.0 mL), and the layers were separated. The aqueous phase was extracted with Et_2O (3 x 100.0 mL). The combined organic layer was washed with brine (200.0 mL), dried over anhydrous Na_2SO_4, filtered, and concentrated under reduced pressure. Purification by flash column chromatography on silica gel (2:1 hexanes/EtOAc) afforded homopropargylic alcohol **17** (3.48 g, 76 %) as a colorless oil.

R_f 0.20 (hexanes/EtOAc, 7:3); **^{1}H NMR** ($CDCl_3$, 400 MHz) δ 7.38-7.29 (m, 5H), 4.60 (s, 2H), 4.18 (t, 2H, J = 2.0 Hz), 3.75 (q, 2H, J = 6.3 Hz), 2.54 (tt, 2H, J = 6.2, 2.2 Hz), 1.75 (br t, 1H, J = 5.6 Hz) ppm; **^{13}C NMR** ($CDCl_3$, 100 MHz) δ 137.3, 128.4, 128.1, 127.9, 84.0, 77.43, 71.6, 60.8, 57.7, 23.0 ppm; **IR** (thin film, cm^{-1}) 3390, 3030, 2857, 1496, 1454, 1354, 1262, 1206, 1132, 1047, 1027, 737, 697; **HRMS** (+APCI) calculated for $C_{12}H_{15}O_2$ $[M+H]^+$ 191.1072, found 191.1063.

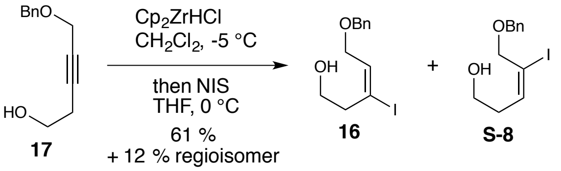

Vinyl Iodide 16:

A solution of homopropargylic alcohol **17** (4.79 g, 25.2 mmol, 1.0 equiv.) in CH_2Cl_2 (126.0 mL) at -78 °C was added via cannula into a suspension of Cp_2ZrHCl (19.5 g, 75.5 mmol, 3.0 equiv.) in CH_2Cl_2 (126.0 mL) at -5 °C. The resulting mixture was stirred for three hours. A solution of NIS (11.3 g, 50.4 mole, 2.0 equiv.) in THF (126.0 mL) at 0 °C was added to the reaction mixture via cannula. The resulting suspension was stirred at 0 °C for 30 minutes. A 1:1 mixture of saturated aqueous $NaHCO_3$/20% aqueous Na_2SO_3 (200.0 mL) was added, and the biphasic mixture was stirred for 15 minutes. The mixture was filtered through celite, and the filter cake was washed with Et_2O (3 x 150.0 mL). The organic layer was separated, and the aqueous phase was extracted with Et_2O (3 x 75.0 mL). The combined organic layer was washed with brine (300.0 mL), dried over anhydrous $MgSO_4$, filtered, and concentrated under reduced pressure. Purification by flash column chromatography on silica gel (4:1 hexanes/EtOAc) afforded vinyl iodide **16** (4.89 g, 61 %) as a black oil.

R_f 0.31 (7:3 hexanes/EtOAc); **^{1}H NMR** ($CDCl_3$, 600 MHz) δ 7.38-7.30 (m, 5H), 6.62 (t, 1H, J = 6.9 Hz), 4.53 (s, 2H), 3.98 (d, 2H, J = 6.9 Hz), 3.74 (t, 2H, J = 5.1 Hz), 2.72 (t, 2H, J = 5.7 Hz), 2.01 (s, 1H) ppm; **^{13}C NMR** ($CDCl_3$, 150 MHz) δ 140.1, 137.5, 128.6, 128.0, 128.0, 104.3, 72.6, 66.9, 60.6, 42.7 ppm; **IR** (thin film, cm^{-1}) 3377, 3033, 2859, 1629, 1495, 1453, 1358, 1043, 1027, 735, 696; **HRMS** (+APCI) calculated for $C_{12}H_{16}IO_2$ 319.0195 $[M+H]^+$, found 319.0190.

Regioisomeric vinyl iodide **S-8** (0.962 g, 12 %) was also isolated as a red oil.

R_f 0.27 (7:3 hexanes/EtOAc); **^{1}H NMR** ($CDCl_3$, 600 MHz) δ 7.41-7.30 (m, 5H), 6.49 (t, 1H, J = 7.8 Hz), 4.52 (s, 2H), 4.19 (s, 2H), 3.56 (dt, 2H, J = 6.0, 2.1 Hz), 2.74 (br s, 1H), 2.30 (q, 2H, J = 6.8 Hz) ppm; **^{13}C NMR** ($CDCl_3$, 150 MHz) δ 141.9, 137.5, 128.5, 128.1, 127.9, 100.2, 71.6, 71.4, 60.9, 34.5 ppm; **IR** (thin film, cm^{-1}) 3378, 3029, 2860, 1629, 496, 1453, 1356, 1045, 1027, 734, 696; **HRMS** (+APCI) calculated for $C_{12}H_{16}IO_2$ $[M+H]^+$ 319.0195, found 319.0190.

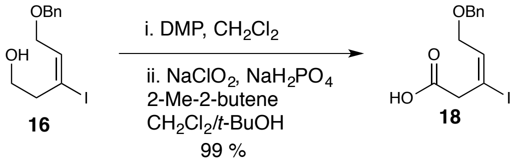

Carboxylic Acid 18:

Dess-Martin periodinane (6.46 g, 15.2 mmol) was added to a stirring solution of vinyl iodide **16** (3.23 g, 10.2 mmol, 1.5 equiv.) in CH_2Cl_2 (100.0 mL), and the resulting suspension was stirred for three hours. The reaction was quenched with a 1:1 mixture of saturated aqueous $NaHCO_3$/20% aqueous Na_2SO_3 (100.0 mL), and the resulting biphasic mixture was stirred for 15 minutes. The layers were separated, and the aqueous phase was extracted with Et_2O (3 x 100.0 mL). The combined organic layer was washed with brine (300.0 mL), dried over anhydrous Na_2SO_4, filtered, and concentrated under reduced pressure. The crude residue was dissolved in CH_2Cl_2 (20.3 mL) and *t*-BuOH (84.6 mL). 2-Methyl-2-butene (46.0 mL) was added, and the resulting mixture was stirred for five minutes. A solution of $NaClO_2$ (9.00 g, 99.5 mmol, 9.8 equiv.) and NaH_2PO_4 (10.9 g, 79.2 mmol, 7.8 equiv.) in H_2O (90.0 mL) was added, and the resulting mixture was stirred for one hour. The reaction was quenched with brine (100.0 mL), and the layers were separated. The aqueous phase was extracted with Et_2O (3 x 100.0 mL). The combined organic layer was washed with brine (2 x 100.0 mL), dried over anhydrous Na_2SO_4, filtered, and concentrated under reduced pressure. Crude carboxylic acid **18** (3.37 g, 99 %) was obtained as a yellow oil which was used without further purification.
$\mathbf{R_f}$ 0.48 (7:3 hexanes/EtOAc); **HRMS** (+APCI) calculated for $C_{12}H_{14}IO_3$ $[M+H]^+$ 332.9988, found 332.9987.

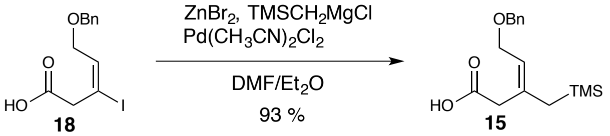

Allylsilane 15:

(Trimethylsilyl)methylmagnesium chloride solution (1.0 M in Et_2O, 40.7 mL, 40.7 mmol, 3.0 equiv.) was added to anhydrous $ZnBr_2$ (9.79 g, 43.5 mmol, 3.2 equiv.), and the resulting suspension was stirred vigorously for 14 hours. DMF (30.0 mL) was added, followed by Et_2O (10.0 mL), and the mixture was stirred for ten minutes. A solution of carboxylic acid **18** (4.51 g, 13.6 mmol, 1.0 equiv.) in DMF (25.0 mL) was added via cannula, and the resulting suspension was cooled to 0 °C. A solution of $Pd(CH_3CN)_2Cl_2$ (0.352 g, 1.36 mmol) in DMF (5.0 mL) was added over five minutes, and the resulting mixture was stirred for two hours, warmed to room temperature, and stirred for 30 minutes. The reaction mixture was cooled to 0 °C and quenched with saturated aqueous NH_4Cl (100.0 mL). EtOAc (200.0 mL) was added, and the mixture was stirred for 15 minutes. The layers were separated, and the aqueous phase was extracted with EtOAc (3 x 125.0 mL). The combined organic layer was washed with brine (300.0 mL), filtered through celite, dried over anhydrous $MgSO_4$, filtered, and concentrated under reduced pressure. Crude allylsilane **15** (3.76 g, 93 %) was obtained as a yellow oil, and was used without further purification.

R$_f$ 0.20 (7:3 hexanes/EtOAc); **^{1}H NMR** ($CDCl_3$, 400 MHz) δ 8.75 (br s, 1H), 7.39-7.28 (m, 5H), 5.51 (t, 1H, J = 6.9 Hz), 4.56 (s, 2H), 4.03 (d, 2H, J = 6.9 Hz), 3.06 (s, 2H), 1.67 (s, 2H), 0.06 (s, 9H) ppm; **^{13}C NMR** ($CDCl_3$, 150 MHz) δ 175.1, 137.8, 136.8 128.6, 128.1, 128.0, 122.6, 72.3, 66.3, 39.3, 28.1, -1.2 ppm; **IR** (thin film, cm^{-1}) 3550-2560, 2952, 1707, 1657, 1247, 840; **HRMS** (+APCI) calculated for $C_{16}H_{25}O_3Si$ $[M+H]^+$ 293.1573, found 293.1565.

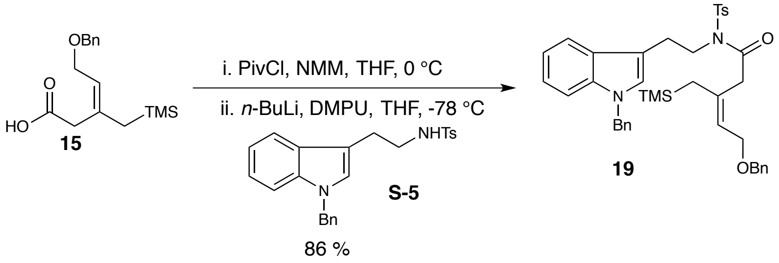

***N*-Tosylamide 19:**

A solution of sulfonamide **S-5** (339.0 mg, 0.838 mmol, 1.0 equiv.) in THF (10.8 mL) was cooled to -78 °C. *n*-BuLi (1.59 M in hexanes, 0.63 mL, 1.00 mmol, 1.2 equiv.) was added over 15 minutes, and the resulting solution was stirred at -78 °C for one hour. DMPU (1.2 mL) was added, and the solution was stirred for 30 minutes. In a separate flask, a solution of allylsilane **15** (319.0 mg, 1.09 mmol, 1.3 equiv.) in THF (11.0 mL) was cooled to 0 °C. *N*-methylmorpholine (0.13 mL, 1.20 mmol, 1.4 equiv.) was added to the carboxylic acid solution, followed by the addition of pivaloyl chloride (0.13 mL, 1.10 mmol, 1.3 equiv). The resulting mixture was stirred for 45 minutes at 0 °C. Stirring was discontinued, and the suspension was allowed to settle for one hour. The yellow supernatant was transferred to a flask pre-cooled to -78 °C. THF (11.0 mL) was added to the white precipitate, and the resulting suspension was stirred for two minutes. Stirring was discontinued, and the suspension was allowed to settle for one hour. The yellow supernatant was separated by syringe and combined with the rest of the mixed anhydride solution. The sulfonamide solution was quickly added to the mixed anhydride solution via cannula, and the solution was stirred at -78 °C for four hours. The reaction was quenched with H_2O (20.0 mL), and the layers were separated. The aqueous phase was extracted with Et_2O (3 x 20.0 mL). The combined organic layer was washed with brine (50 mL), dried over anhydrous Mg_2SO_4, filtered, and concentrated under reduced pressure. Purification by flash column chromatography on silica gel (17:3 hexanes/EtOAc) afforded *N*-tosylamide **19** (488.0 mg, 86 %) as a colorless oil.

R$_f$ 0.56 (7:3 hexanes/EtOAc); **^{1}H NMR** ($CDCl_3$, 400 MHz) δ 7.82-7.76 (m, 3H), 7.30-7.27 (m, 15H), 6.98 (s, 1H), 5.43 (t, 1H, J = 6.7 Hz), 5.26 (s, 2H), 4.35 (s, 2H), 4.06-4.02 (m, 2H), 3.78 (d, 2H, J = 6.7 Hz), 3.32 (s, 2H), 3.21-3.17 (m, 2H), 2.42 (s, 3H), 1.49 (s, 2H), -0.03 (s, 9H) ppm; **^{13}C NMR** ($CDCl_3$, 100 MHz) δ 170.5, 145.0, 138.6, 137.7, 137.1, 136.8, 135.1, 130.1, 128.9, 128.5, 128.2, 127.8, 127.8, 127.7, 127.7, 127.1, 126.8, 123.5, 122.1, 119.6, 119.5, 111.5, 109.9, 71.7, 66.5, 50.1, 48.2, 40.4, 28.5, 26.3, 21.8, -1.2 ppm; **IR** (thin film, cm^{-1}) 3030, 2952, 1698, 1495, 1467, 1453, 1352, 1247, 1159, 1088, 850, 739; **HRMS** (+ESI) calculated for $C_{40}H_{50}N_3O_4SSi$ $[M+NH_4]^+$ 696.3291, found 696.3299.

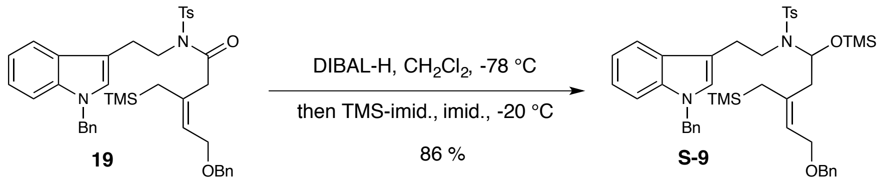

Hemiaminal S-9:

A solution of *N*-tosylamide **19** (458.0 mg, 0.68 mmol, 1.0 equiv.) in CH_2Cl_2 (9.0 mL) was cooled to -78 °C. DIBAL-H (1.0 M in CH_2Cl_2, 1.35 mL, 1.35 mmol, 2.0 equiv.) was slowly added over 15 minutes, and the reaction mixture was stirred for one hour. A solution of imidazole (55.1 mg, 0.81 mmol, 1.2 equiv.) in CH_2Cl_2 (2.2 mL) was added, followed by trimethylsilyl imidazole (0.40 mL, 2.70 mmol, 4.0 equiv.). The mixture was warmed to -25 °C and stirred overnight. The reaction was warmed up to 0 °C and stirred for three hours. The reaction was quenched by slow addition of aqueous 15 % Rochelle's salt solution (10.0 mL). Et_2O (30.0 mL) was added, and the mixture was stirred vigorously at room temperature until both layers were clear. The layers were separated, and the aqueous phase was extracted with Et_2O (2 x 30.0 mL). The combined organic layer was washed with brine (2 x 30.0 mL), dried over anhydrous Na_2SO_4, filtered, and concentrated under reduced pressure. Purification by flash column chromatography on silica gel (10:1 hexanes/EtOAc with 1 % Et_3N) afforded hemiaminal **S-9** (437.6 mg, 86 %) as a colorless oil.

R_f 0.62 (7:3 hexanes/EtOAc); **^{1}H NMR** ($CDCl_3$, 400 MHz) δ 7.78-7.76 (m, 3H), 7.36-7.20 (m, 12H), 7.18-7.14 (m, 3H), 6.98 (s, 1H), 5.33 (dd, 1H, *J* = 9.6, 2.4 Hz), 5.35 (t, 1H, *J* = 6.8 Hz), 5.31 (s, 2H), 4.48 (q, 2H, *J* = 10.3 Hz), 4.14 (dd, 1H, *J* = 12.4, 7.6 Hz), 4.01 (dd, 1H, *J* = 12.4, 6.4 Hz), 3.60 (ddd, 1H, *J* = 14.6, 11.8, 5.6 Hz), 3.45 (ddd, 1H, *J* = 14.6, 11.8, 4.8 Hz), 3.25 (dt, 1H, *J* = 12.6, 4.8 Hz), 3.16 (dt, 1H, *J* = 12.6, 5.6 Hz), 2.56 (dd, 1H, *J* = 12.8, 9.6 Hz), 2.39 (s, 3H), 1.90 (dd, 1H, *J* = 12.8, 2.4 Hz), 1.64 (d, 1H, *J* = 13.4 Hz), 1.40 (d, 1H, *J* = 13.4 Hz), 0.12 (s, 9H), 0.01 (s, 9H) ppm; **^{13}C NMR** ($CDCl_3$, 100 MHz) δ 143.2, 138.7, 138.6, 137.7, 137.0, 136.7, 129.8, 128.8, 128.4, 128.1, 127.7, 127.6, 127.5, 127.1, 126.9, 126.2, 126.1, 122.9, 121.9, 119.3, 112.6, 109.9, 81.4, 71.6, 66.7, 49.9, 44.0, 40.2, 28.0, 27.8, 21.5, 0.1, -1.3 ppm; **IR** (thin film, cm^{-1}) 3030, 2953, 1467, 1453, 1334, 1249, 1159, 843, 735; **HRMS** (+ESI) calculated for $C_{43}H_{60}N_3O_4SSi_2$ $[M+NH_4]^+$ 770.3843, found 770.4864.

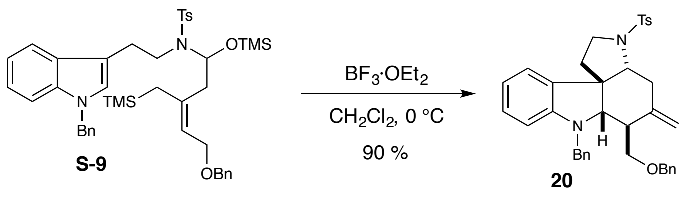

Tetracycle 20:

A solution of hemiaminal **S-9** (688.0 mg, 0.91 mmol, 1.0 equiv.) in CH_2Cl_2 (22 mL) was cooled to 0 °C. Freshly distilled $BF_3{\cdot}OEt_2$ (0.58 mL, 4.56 mmol, 5.0 equiv.) was added drop-wise over three minutes, and the mixture was stirred at 0 °C for one hour. The reaction was quenched with saturated aqueous $NaHCO_3$ (12.0 mL), and the resulting biphasic mixture was stirred vigorously for 15 minutes. The layers were separated, and the aqueous phase was extracted with CH_2Cl_2 (3 x 30.0 mL). The combined organic layer was washed with brine (100.0 mL), dried over anhydrous Na_2SO_4, filtered, and concentrated under reduced pressure. Purification by flash column chromatography on silica gel (7:2 hexanes/EtOAc) afforded tetracycle **20** (484.0 mg, 90 %) as an amorphous white solid.

R_f 0.40 (4:1 hexanes/EtOAc); **^{1}H NMR** ($CDCl_3$, 400 MHz) δ 7.69 (d, 2H, *J* = 8.4 Hz), 7.38-7.20 (m, 13H), 7.03 (t, 1H, *J* = 7.6 Hz), 6.64 (t, 1H, *J* = 7.6 Hz), 6.26 (d, 1H, *J* = 8.0 Hz), 4.74 (s, 2H), 4.47-4.39 (m, 3H), 4.26 (d, 1H, *J* = 16.0 Hz), 3.67 (td, 1H, *J* = 10.8, 7.2 Hz), 3.49 (s, 1H), 3.41 (t, 1H, *J* = 10.4 Hz), 3.36-3.31 (m, 2H), 3.26 (dd, 1H, *J* = 9.6, 7.6 Hz), 3.08 (dd, 1H, *J* = 16.0, 6.8 Hz), 2.70 (dd, 1H, *J* = 16.0, 11.6 Hz), 2.60 (t, 1H, *J* = 6.8 Hz), 2.45 (s, 3H), 1.82 (dd, 1H, *J* = 11.6, 6.4 Hz), 1.36 (q, 1H, *J* = 10.4 Hz) ppm; **^{13}C NMR** ($CDCl_3$, 150 MHz) 150.3, 143.8, 142.3, 138.5, 138.0, 132.6, 130.7, 129.8, 128.6, 128.5, 128.5, 127.8, 127.8, 127.6, 127.4, 127.2, 124.8, 117.1, 114.6, 106.0, 73.0, 73.0, 68.9, 59.3, 55.0, 48.6, 47.8, 46.1, 36.5, 33.6, 21.7 ppm; **IR** (thin film, cm^{-1}) 2858, 1599, 1481, 1347, 1160, 1090, 1026, 734, 664, 549; **HRMS** (+NSI) calculated for $C_{37}H_{38}N_2O_3S$ $[M+H]^+$ 591.2676, found 591.2690.

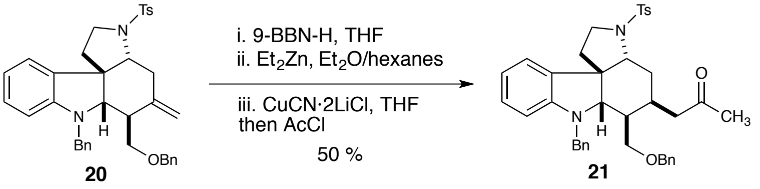

Ketone 21:

To tetracycle **20** (510.0 mg, 0.86 mmol, 1.0 equiv.) in a Schlenk flask was added 9-BBN-H (0.5 M in THF, 2.33 mL, 1.16 mmol, 1.35 equiv.), and the resulting solution was stirred at room temperature overnight. After removal of the solvent with vacuum (0.1 mmHg, 25 °C, 1 hour), Et_2O (5.0 mL) and Et_2Zn (1 M in hexanes, 12.9 mL, 12.9 mmol, 15.0 equiv.) were added, and the resulting solution was stirred for three hours. After removal of the solvent with vacuum (0.1 mmHg, 25 °C, 1 hour), the resultant grey-black residue was diluted with THF (13.0 mL), and the mixture was cooled to -78 °C. A freshly prepared solution of CuCN (1.16 g, 12.9 mmol, 15.0 equiv.) and LiCl (1.11 g, 25.9 mmol, 30.0 equiv.) in THF (6.0 mL) was added over ten minutes, and the mixture was stirred at -78 °C for 30 minutes. Acetyl chloride (2.7 mL, 38.7 mmol, 45.0 equiv.) was

added slowly over ten minutes, and the resulting solution was warmed to -20 °C over 12 hours. The reaction was quenched with saturated aqueous NH_4Cl (50.0 mL) containing 30 % aqueous NH_3OH (3.0 mL) and diluted with EtOAc (100.0 mL). The resulting biphasic mixture was stirred vigorously for 15 minutes. The layers were separated, and the aqueous phase was extracted with EtOAc (2 x 50.0 mL). The combined organic layer was washed with brine (150.0 mL), dried over anhydrous $MgSO_4$, filtered, and concentrated under reduced pressure. Purification by flash column chromatography on silica gel (4:1 to 1:1 hexanes/EtOAc) afforded ketone **21** (275.0 mg, 50 %) as an amorphous white solid.

R_f 0.50 (7:3 hexanes/EtOAc); **^{1}H NMR** ($CDCl_3$, 600 MHz) δ 7.70 (d, *J* = 8.0 Hz, 2H), 7.35-7.28 (m, 5H), 7.27-7.23 (m, 5H), 7.17 (d, *J* = 7.8 Hz, 1H), 7.04 (dt, *J* = 7.7, 1.3 Hz, 1H), 6.60 (dt, *J* = 7.4, 1.0 Hz, 1H), 6.31 (d, *J* = 7.8 Hz, 1H), 4.53 (d, *J* = 15.6 Hz, 1H), 4.38- 4.27 (m, 2H), 4.25 (d, *J* = 15.7 Hz, 1H), 3.66 (td, *J* = 10.9, 7.0 Hz, 1H), 3.45-3.41 (m, 2H), 3.39 (dd, *J* = 11.4, 8.0 Hz, 1H), 3.28 (dd, *J* = 9.6, 5.5 Hz, 1H), 3.14 (dd, *J* = 10.8, 7.4 Hz, 1H), 3.02 (dd, *J* = 9.6, 5.4 Hz, 1H), 2.53-2.45 (m, 1H), 2.43 (s, 3H), 2.26-2.18 (m, 3H), 2.06-1.99 (m, 1H), 1.94 (s, 3H), 1.85-1.79 (m, 2H), 1.65-1.60 (m, 1H) ppm; **^{13}C NMR** ($CDCl_3$, 101 MHz) δ 207.5, 150.6, 143.9, 138.5, 138.0, 133.1, 131.3, 129.9, 128.8, 128.7, 128.6, 128.0, 127.8, 127.6, 127.3, 124.4, 117.0, 105.7, 73.1, 70.8, 70.0, 59.5, 53.8, 48.2, 47.8, 47.3, 39.5, 37.4, 30.9, 30.1, 27.7, 21.8 ppm; **IR** (thin film, cm^{-1}) 2918, 1712, 1599, 1484, 1347, 1161, 1091, 732; **HRMS** (+APCI) calculated for $C_{39}H_{43}N_2O_4S$ $[M+H]^+$ 635.2938, found 635.2943.

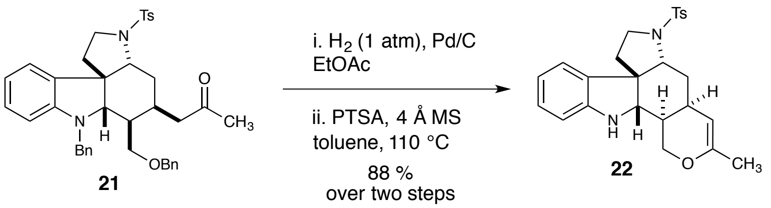

Dihydropyran 22:

To a solution of ketone **21** (133.3 mg, 0.21 mmol, 1.0 equiv.) in EtOAc (12.0 mL) was added Pd/C (5 wt %, 446 mg, 0.21 mmol, 1.0 equiv.). The mixture was purged with hydrogen gas for 5 minutes, and the placed under an atmosphere of hydrogen (balloon) for four hours. The resulting suspension was filtered through celite, and the filter cake was washed with EtOAc (4 x 25.0 mL). The combined filtrate was concentrated under reduced pressure, and the crude residue was dissolved in anhydrous toluene (5.0 mL). *p*-Toluenesulfonic acid (8.0 mg, 0.04 mmol, 0.2 equiv.) was added, followed by activated powdered 4 Å molecular sieves (50.0 mg). The flask was equipped with a reflux condenser, and the suspension was heated at 110 °C for two hours. The mixture was cooled to room temperature and then directly purified by flash column chromatography on silica gel (10:4 to 8:5 hexanes/EtOAc), affording dihydropyran **22** (80.7 mg, 88 % over two steps) as an amorphous white solid.

R_f 0.50 (1:1 hexanes/EtOAc); **^{1}H NMR** ($CDCl_3$, 600 MHz) δ 7.76 (d, 2H, *J* = 8.1 Hz), 7.47 (d, 1H, *J* = 7.4 Hz), 7.37 (d, 2H, *J* = 8.1 Hz), 7.09 (t, 1H, *J* = 7.6 Hz), 6.83 (t, 1H, *J* = 7.5 Hz), 6.71 (d, 1H, *J* = 7.8 Hz), 4.26 (s, 1H), 4.05-3.97 (m, 2H), 3.88 (dd, 1H, *J* = 11.2, 1.7 Hz), 3.54 (dt, 1H, *J* = 11.1, 6.9 Hz), 3.40 (t, 1H, *J* = 10.4 Hz), 3.33 (d, 1H, *J* =

8.3 Hz), 3.07 (dd, 1H, *J* = 12.6, 3.0 Hz), 2.67 (br s, 1H), 2.48 (s, 3H), 2.42 (dt, 1H, *J* = 12.9, 2.7 Hz), 1.99 (dt, 1H, *J* = 12.8, 4.5 Hz), 1.70 (s, 3H), 1.67 (dd, 1H, *J* = 11.8, 6.8 Hz), 1.47-1.40 (m, 2H) ppm; **^{13}C NMR** ($CDCl_3$, 150 MHz) δ 152.0, 149.6, 143.9, 132.6, 131.2, 129.7, 128.2, 128.2, 125.6, 119.9, 111.7, 100.0, 67.3, 63.5, 58.7, 55.4, 47.5, 37.5, 35.8, 31.5, 30.8, 21.8, 20.0 ppm; **IR** (thin film, cm^{-1}) 3356, 2923, 2855, 1676, 1599, 1462, 1347, 1327, 1162, 1091.3; **HRMS** (+APCI) calculated for $C_{25}H_{29}N_2O_3S$ $[M+H]^+$ 437.1899, found 437.1895.

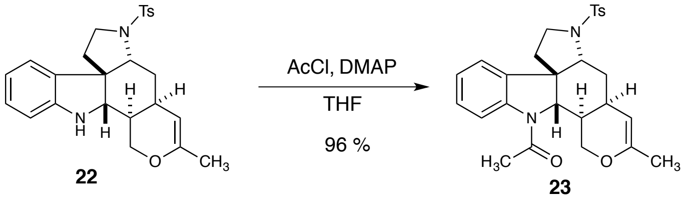

Acetamide 23:

Acetyl chloride (0.067 mL, 0.94 mmol, 5.0 equiv.) was added drop-wise to a solution of dihydropyran **22** (82.0 mg, 0.188 mmol, 1.0 equiv.) and 4-dimethylaminopyridine (0.103 g, 0.845 mmol, 4.5 equiv.) in THF (3.8 mL), and the resulting mixture was stirred for one hour. The reaction was quenched with saturated aqueous $NaHCO_3$ (10.0 mL), and the resulting biphasic mixture was stirred vigorously for 15 minutes. The layers were separated, and the aqueous phase was extracted with EtOAc (3 x 10.0 mL). The combined organic layers were washed with brine (10.0 mL), dried over anhydrous Na_2SO_4, filtered, and concentrated under reduced pressure. Purification by flash column chromatography on silica gel (2:1 to 1:1 hexanes/EtOAc) afforded acetamide **23** (83.7 mg, 96 %) as an amorphous white solid.

R_f 0.35 (1:1 hexanes/EtOAc); **^{1}H NMR** ($CDCl_3$, 600 MHz) (1:0.6 mixture of rotamers) δ 8.01 (d, 1H, *J* = 8.0 Hz), 7.78-7.73 (m, 3.2H), 7.63 (d, 0.6H, *J* = 7.4 Hz), 7.56 (d, 1H, *J* = 7.5 Hz), 7.43-7.36 (m, 3.2H), 7.29-7.26 (m, 1.6H), 7.16-7.10 (m, 2.2H), 4.71 (d, 0.6H, *J* = 8.5 Hz), 4.28 (s, 1H), 4.23-4.22 (m, 1.2H), 4.11 (dd, 1H, *J* = 11.5, 1.7 Hz), 4.03 (d, 1H, *J* = 8.6 Hz), 3.88 (d, 1H, *J* = 11.6 Hz), 3.77 (dd, 0.6H, *J* = 10.6, 1.5 Hz), 3.54-3.49 (m, 1.6H), 3.46-3.38 (m, 1.6H), 3.12 (dd, 1H, *J* = 12.7, 3.2 Hz), 3.07 (dd, 0.6H, *J* = 12.6, 2.9 Hz), 2.73 (br s, 1H), 2.66 (br s, 0.6H), 2.49 (s, 4.8H), 2.44 (dt, 1H, *J* = 13.2, 3.0 Hz), 2.41-2.35 (m, 2.4H), 2.29 (s, 3H), 1.99 (dt, 1H, *J* = 12.9, 4.5 Hz), 1.92 (dt, 0.6H, *J* = 12.9, 4.6 Hz), 1.78-1.71 (m, 1.6H), 1.67-1.59 (m, 3.2H), 1.53-1.43 (m, 3H) ppm; **^{13}C NMR** ($CDCl_3$, 150 MHz) (1:0.6 mixture of rotamers) 168.6, 168.0, 153.1, 152.6, 144.3, 144.2, 141.5, 141.2, 136.7, 134.9, 132.5, 132.0, 129.9, 129.9, 128.5, 128.3, 128.2, 128.2, 126.3, 125.1, 125.0, 124.7, 120.2, 117.7, 99.8, 99.1, 67.4, 66.7, 65.7, 64.5, 58.6, 58.5, 54.9, 54.1, 47.1, 46.9, 37.1, 37.0, 35.8, 35.5, 32.2, 31.6, 30.2, 26.7, 23.4, 23.4, 21.8, 21.8, 20.2, 20.1; **IR** (thin film, cm^{-1}) 2922.2 (w), 1655.8 (s), 1597.6 (w), 1473.4 (m), 1461.7 (m), 1393.9 (m), 1349.9 (m), 1332.8 (w), 1163.1 (s), 1091.6 (m), 730.2 (m) ppm; **HRMS** (+APCI) calculated for $C_{27}H_{31}N_2O_4S$ $[M+H]^+$ 479.2005, found 479.1999.

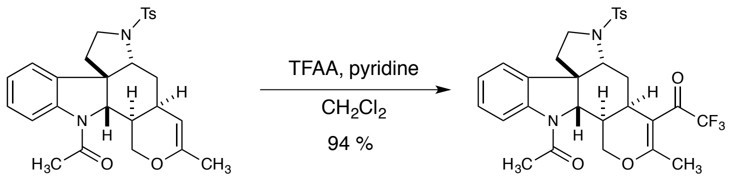

23 **24**

Trifluoromethylketone 24:

A solution of acetamide **91** (83.7 mg, 0.18 mmol, 1.0 equiv.) in CH_2Cl_2 (3.7 mL) was cooled to 0 °C. Pyridine (0.062 mL, 0.77 mmol, 4.4 equiv.) was added followed by the drop-wise addition of trifluoroacetic anhydride (0.1 mL, 0.72 mmol, 1.0 equiv.). The resulting mixture was stirred at 0 °C for one hour and then stirred at room temperature for ten hours. The reaction was quenched with aqueous phosphate buffer (pH = 7.0, 10.0 mL), and the resulting biphasic mixture was stirred vigorously for 15 minutes. The layers were separated, and the aqueous phase was extracted with EtOAc (3 x 20.0 mL). The combined organic layer was washed with brine (10.0 mL), dried over anhydrous Na_2SO_4, filtered, and concentrated under reduced pressure. Purification by flash column chromatography on silica gel (1:1 to 1:2 hexanes/EtOAc) afforded trifluoromethylketone **24** (97.2 mg, 94 %).

^{1}H NMR ($CDCl_3$, 600 MHz) (1:0.4 mixture of rotamers) δ 8.06 (d, 0.4H, *J* = 8.0 Hz), 7.69 (d, 2.8H, *J* = 7.8 Hz), 7.62 (d, 1H, *J* = 7.4 Hz), 7.52 (d, 0.4H, *J* = 7.4 Hz), 7.41 (d, 6H, *J* = 7.8 Hz), 7.33 – 7.27 (m, 1.4H), 7.13 (m, 1.4H), 4.47 (d, 2H, *J* = 3.4 Hz), 4.21 (dd, 1H, *J* = 10.7, 3.5 Hz), 4.18 – 4.13 (m, 1H), 4.03 – 3.89 (m, 1.8H), 3.62 – 3.53 (m, 1.4H), 3.43 (dd, 1.4H, *J* = 11.3, 9.6 Hz), 3.26 (dd, 0.4H, *J* = 11.0, 6.7 Hz), 3.21 (dd, 1H, *J* = 11.0, 6.7 Hz), 2.89 (m, 0.4H), 2.74 (m, 1H), 2.48 (s, 4.4 H), 2.41 (s, 3H), 2.35 – 2.17 (m, 4.8H), 2.14 – 2.07 (m, 4.8H), 2.03 – 1.91 (m, 1.4H), 1.78 - 1.68 (m, 1.4H), 1.63 – 1.44 (m, 1.8H), 1.41 (d, 0.4 H, *J* = 5.6 Hz), 1.39 (dd, 0.4H, *J* = 12.5, 5.6 Hz).) ppm; **^{13}C NMR** ($CDCl_3$, 150 MHz) 169.4, 168.3, 167.6, 144.5, 144.4, 141.5, 140.8, 135.5, 133.5, 132.7, 132.4, 130.2, 129.2, 129.0, 128.0, 125.9, 125.2, 124.78, 124.6, 119.0, 117.6, 115.8, 110.6, 110.3, 99.7, 68.2, 67.7, 65.6, 64.2, 57.5, 57.2, 53.7, 52.1, 47.1, 46.9, 39.0, 38.0, 36.1, 36.0, 30.9, 30.7, 27.7, 23.9, 23.7, 21.8, 21.1, 20.7, 17.6 ppm; **^{19}F NMR** ($CDCl_3$, 376 MHz) (1:0.4 mixture of rotamers) δ -73.8 (major), -74.1 (minor) ppm; **IR** (thin film, cm^{-1}) 2972, 1653, 1597, 1544, 1393, 1162, 906, 726; **HRMS** (+APCI) calculated for $C_{27}H_{28}F_3N_2O_4S$ $[M+H]^+$ 533.1722, found 533.1716.

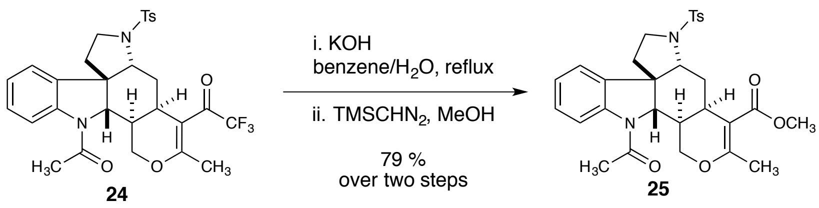

Ester 25:

To a solution of trifluoromethylketone **24** (97.7 mg, 0.17 mmol, 1.0 equiv.) in benzene (2.5 mL), was added powdered KOH (17.0 mg, 0.30 mmol, 1.8 equiv.) and H_2O (15.0 μL). The reaction mixture was heated to reflux overnight (oil bath temperature of 100 °C). The light yellow suspension was cooled to room temperature. EtOAc (20.0 mL) was added, and the pH of the mixture was adjusted to 7.0 by the slow addition of 0.5 N HCl. The layers were separated, and the aqueous phase was extracted with EtOAc (3 x 30.0 mL). The combined organic layer was washed with brine (20.0 mL), dried over anhydrous Na_2SO_4, filtered, and concentrated under reduced pressure. The crude residue was brought up in MeOH (2.0 mL) and toluene (3.0 mL). Excess $TMSCHN_2$ (2M in Et_2O, 170.0 μL) was added drop-wise, and the resulting mixture was stirred at room temperature for one hour. The reaction mixture was quenched by the slow addition of AcOH (1.0 mL). The mixture was concentrated under reduced pressure, and EtOAc (30.0 mL) and saturated $NaHCO_3$ solution (10.0 mL) were added to the residue. The layers were separated, and the aqueous phase was extracted with EtOAc (3 x 20.0 mL). The combined organic layer was washed with brine (20.0 mL), dried over anhydrous Na_2SO_4, filtered, and concentrated under reduced pressure. Purification by flash column chromatography on silica gel (1:1 hexanes/EtOAc) afforded Ester **25** (72.1 mg, 79 % over two steps).

R_f 0.40 (1:1 hexanes/EtOAc); **^{1}H NMR** ($CDCl_3$, 600 MHz) ($CDCl_3$, 600 MHz) (1:0.7 mixture of rotamers) δ 8.04 (d, 0.7H, *J* = 8.0 Hz), 7.72 – 7.65 (m, 3.4H), 7.63 (d, 1H, *J* = 8.0 Hz), 7.56 (d, 0.7H, *J* = 7.5 Hz), 7.41 – 7.38 (m, 3.4H), 7.32 – 7.27 (m, 1.7H), 7.17 – 7.10 (m, 1.7H), 4.57 (d, 1H, *J* = 6.0 Hz), 4.07 – 3.98 (m, 1.7H), 3.97 (d, 0.7H, *J* = 6.5 Hz), 3.95 – 3.89 (m, 1.7H), 3.79 (s, 1.8H), 3.75 (s, 3H), 3.53 (tt, 1.7H, *J* = 11.4, 6.3 Hz), 3.37 – 3.31, (m, 1.7H), 3.11 (dd, 0.7H, *J* = 12.5, 4.4 Hz), 3.04 (dd, 1H, *J* = 12.5, 4.4 Hz), 2.92 – 2.85 (m, 0.7H), 2.83 – 2.75 (m, 1H), 2.70 (dt, 0.7H, *J* = 14.1, 4.4 Hz), 2.61 (dt, 1H, *J* = 14.1, 4.4 Hz), 2.47 (s, 4.8H), 2.38 (s, 3H), 2.26 (s, 1.8H), 2.21 (s, 3H), 2.19 (s, 1.8H), 2.14 – 2.07 (m, 1.7H), 1.90 – 1.77 (m, 1.7H), 1.64 (dd, 0.7 H, *J* = 11.8, 6.8 Hz), 1.50 – 1.37 (m, 1.7H) ppm; **^{13}C NMR** ($CDCl_3$, 150 MHz) δ 168.5, 168.2, 167.8, 164.7, 163.3, 144.2, 141.1, 136.2, 134.3, 132.1, 130.1, 130.0, 130.0, 128.8, 128.6, 126.3, 125.0, 124.7, 119.6, 116.7, 106.2, 105.6, 67.9, 67.3, 66.1, 64.9, 58.7, 58.5, 54.6, 53.3, 51.6, 51.4, 47.0, 46.8, 39.2, 38.9, 36.0, 35.8, 31.9, 30.9, 29.1, 29.0, 23.7, 21.8, 20.1, 19.8 ppm; **IR** (thin film, cm^{-1}) 2949, 1705, 1661, 1476, 1393, 1163, 1092, 733, 666; **HRMS** (+NSI) calculated for $C_{29}H_{33}N_2O_6S$ $[M+H]^+$ 537.2054, found 537.2054.

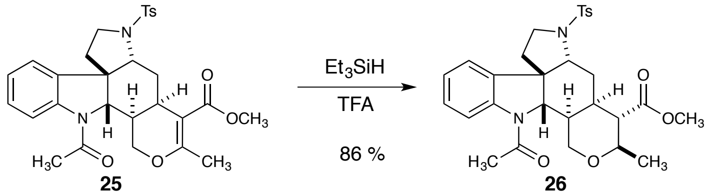

N-Tosyl-20-epi-malagashanine 26:

To a solution of ester **25** (22.0 mg, 0.041 mmol, 1.0 equiv.) in TFA (2.0 mL) was added freshly distilled Et_3SiH (100.0 μL, 0.628 mmol, 15.3 equiv.) at room temperature, and the mixture was stirred for 1 hour. *i*-PrOAc (20.0 mL) was added, followed by slow addition of saturated aqueous $NaHCO_3$ (2.0 mL). The resulting biphasic mixture was stirred vigorously for 15 minutes. The organic layer was separated, and the aqueous phase was extracted with *i*-PrOAc (3 x 20.0 mL). The combined organic layer was washed with brine (10.0 mL), dried over anhydrous Na_2SO_4, filtered, and concentrated under reduced pressure. Purification by flash column chromatography on silica gel (1:1 hexanes/EtOAc) afforded *N*-tosylepimalagashanine **26** (19.0 mg, 86 %).

R_f 0.30 (1:1 hexanes/EtOAc); **^{1}H NMR** ($CDCl_3$, 600 MHz) (1:0.7 mixture of rotamers) δ 7.99 (d, 1 H, *J* = 7.6 Hz), 7.87 – 7.81 (m, 3.4H), 7.68 (d, 0.7H, *J* = 7.6 Hz), 7.62 (d, 0.7H, *J* = 9.8 Hz), 7.44 (d, 3.4H, *J* = 7.8 Hz), 7.29 – 7.25 (m, 3.4H), 7.15 – 7.09 (m, 1.7H), 4.87 (d, 2H, *J* = 9.8 Hz), 4.18 (d, 1H, *J* = 9.8 Hz), 4.02 (d, 1H, *J* = 11.8 Hz), 3.88 – 3.83 (m, 5.1H), 3.62 – 3.54 (m, 1H), 3.53 – 3.41 (m, 5.1H), 3.34 (dd, 1H, *J* = 13.0, 3.4 Hz), 3.30 – 3.23 (m, 1.7H), 2.53 – 2.49 (m, 5.1H), 2.47 – 2.33 (m, 8.5H), 2.26 (s, 3.5H), 2.01 – 1.86 (m, 1.7 H), 1.64 – 1.55 (m, 4.7H), 1.53 – 1.45 (m, 1.7H) ppm; **^{13}C NMR** ($CDCl_3$, 150 MHz) δ 173.0, 168.1, 144.2, 140.9, 137.1, 135.13, 132.3, 131.1, 130.1, 129.0, 128.6, 128.5, 128.3, 126.3, 125.1, 125.0, 124.9, 120.3, 118.1, 68.9, 68.2, 65.2, 64.1, 59.2, 59.0, 55.8, 52.4, 52.2, 50.5, 50.3, 46.6, 46.5, 38.3, 38.0, 37.4, 37.0, 35.6, 35.3, 32.1, 29.9, 29.6, 29.2, 28.8, 28.6, 27.2, 23.6, 23.2, 22.9, 21.8, 20.5, 20.5, 14.3, 14.3 ppm; **HRMS** (+APCI) calculated for $C_{29}H_{35}N_2O_6S$ $[M+H]^+$ 539.2210, found 539.2208.

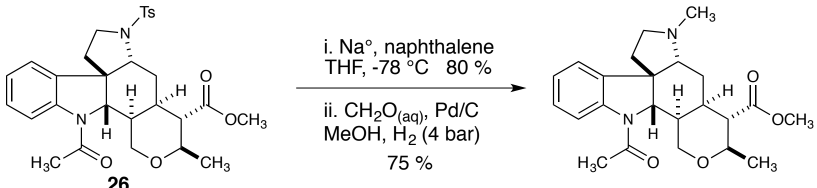

20-Epi-malagashanine 27:

THF (4.4 mL) was added to a flask charged with naphthalene (728.0 mg, 5.68 mmol) and sodium metal (106.0 mg, 4.61 mmol). The resuling dark green mixture was stirred at room temperature for one hour. In a separate flask, a solution of *N*-tosylepimalagashanine **26** (19.3 mg, 0.035 mmol) in THF (1.0 mL) was cooled to -78 °C. The sodium naphthalenide solution (100 μL, 0.056 mmol) was added slowly by syringe until the clear starting material solution turned green, and the reaction was stirred at -78 °C for one hour. The reaction was then quenched with saturated aqueous $NaHCO_3$ (2.0 mL) and warmed to room temperature. EtOAc (10.0 mL) was added, and the layers were separated. The aqueous phase was extracted with EtOAc (3x 10.0 mL). The

combined organic layer was washed with brine (10.0 mL), dried over anhydrous Na_2SO_4, filtered, and concentrated under reduced pressure. Purification by flash column chromatography on silica gel (1:1 hexanes/EtOAc to remove excess naphthalene, then 92:8 CH_2Cl_2/methanol) afforded the desired free amine (9.0 mg, 80 %). To a solution of the free amine (9.0 mg, 0.023 mmol, 1.0 equiv.) in CH_3OH (2.0 mL) was added formaldehyde solution (37 wt % in H_2O, 20.0 μL, 0.116 mmol, 5.0 equiv.) and Pd/C (10 wt %, 0.012g). The heterogeneous mixture was then submitted to hydrogen gas (4 bar) for four hours. The mixture was filtered through celite, and the filter cake was washed with MeOH (2 x 10.0 mL). The filtrate was concentrated under reduced pressure. Purification by flash column chromatography on silica gel (93:7 CH_2Cl_2/methanol) afforded epimalagashanine **27** (7.0 mg, 75 %) as a white amorphous solid.

R_f 0.35 (93:7 CH_2Cl_2/methanol); **^{1}H NMR** ($CDCl_3$, 600 MHz) (1:0.5 mixture of rotamers) δ 7.97 (d, 1.0H, *J* = 8.0 Hz), 7.77 (d, 0.5H, *J* = 7.5 Hz), 7.64 (d, 1H, *J* = 7.5 Hz), 7.20 – 7.16 (m, 1.5H), 7.09 (d, 1.4H, *J* = 8.0 Hz), 7.06 – 7.00 (m, 1.5H), 4.98 (d, 0.5H, *J* = 9.7 Hz), 4.25 (d, 1.0H, *J* = 9.7 Hz), 4.09 (d, 1.0H, *J* = 11.6 Hz), 3.99 (d, 1.5H, *J* = 12.6 Hz), 3.75 – 3.69 (m, 4.5H), 3.62 – 3.55 (m, 1.5H), 3.54 – 3.39 (m, 3H), 3.27– 3.16 (m, 1.5H), 2.64 (dd, 1.0H, *J* = 12.9, 3.0 Hz), 2.62 – 2.53 (m, 2.5H), 2.37 – 2.29 (m, 9.5H), 2.03 – 1.95 (m, 1.5H), 1.67 – 1.40 (m, 6.0H), 1.23 - 1.18 (m, 4.5H) ppm; **^{13}C NMR** ($CDCl_3$, 150 MHz) δ 173.5, 168.8, 168.3, 140.9, 140.8, 140.1, 138.1, 127.6, 127.3, 127.2, 126.0, 124.6, 124.3, 119.9, 117.7, 76.3, 75.9, 69.1, 68.5, 66.1, 64.8, 64.5, 55.9, 55.0, 52.8, 52.7, 51.8, 51.7, 50.4, 50.2, 41.2, 41.1, 39.2, 39.0, 38.0, 37.7, 37.1, 36.8, 29.9, 27.1, 26.9, 23.8, 23.4, 20.5, 20.4 ppm; **IR** (thin film, cm^{-1}) 2941, 2850, 2231, 2032, 1732, 1658, 1462, 1394, 1109, 761; **HRMS** (+APCI) calculated for $C_{23}H_{31}N_2O_4$ $[M+H]^+$ 399.2278, found 399.2273.

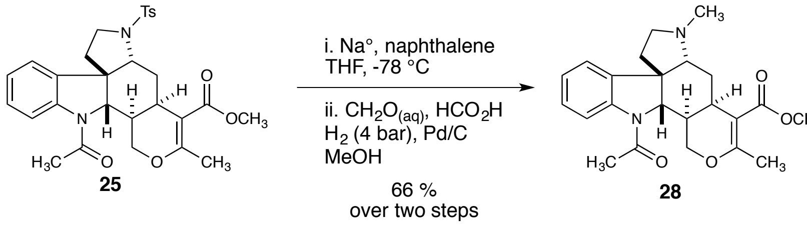

Methylamine 28:

DME (2.0 mL) was added to a flask charged with naphthalene (154.0 mg, 1.2 mmol) and sodium metal (23.0 mg, 1.0 mmol). The resulting dark green mixture was stirred at room temperature for 2 hours. In a separate flask, a solution of *N*-tosyl ester **90** (20.0 mg, 0.037 mmol, 1.0 equiv.) in DME (1.5 mL) was cooled to -78 °C. The sodium naphthalenide solution was added slowly by syringe until the clear starting material solution turned green. The reaction was furthered stirred at -60 °C for an additional hour and then quenched with saturated aqueous $NaHCO_3$ (2.0 mL). The resulting mixture was warmed to room temperature and EtOAc (20.0 mL) was added. The layers were separated, and the aqueous phase was extracted with EtOAc (3x 10.0 mL). The combined organic layer was washed with brine (10.0 mL), dried over anhydrous Na_2SO_4, filtered, and concentrated under reduced pressure to provide the secondary amine, which was used without further purification.

To a solution of secondary amine in CH_3OH (2.0 mL) was added formaldehyde solution (37 wt % in H_2O, 8.7 μL, 0.114 mmol, 3.0 equiv.), formic acid (8.7 μL, 0.228 mmol, 6.0 equiv.), and Pd/C (10 wt %, 41.0 mg, 0.037 mmol, 1.0 equiv.). The heterogeneous mixture was then submitted to hydrogen gas (15 bar) for 4 hours. The mixture was filtered through celite, and the filter cake was washed with MeOH (2 x 20.0 mL). The resulting solution was concentrated under reduced pressure. Purification by flash column chromatography on silica gel (93:7 CH_2Cl_2/methanol) afforded methylamine **28** (9.8 mg, 66 % over two steps) as a white amorphous solid.

R_f 0.25 (93:7 CH_2Cl_2/methanol); **m.p.** 62-64 °C; **^{1}H NMR** ($CDCl_3$, 400 MHz) (1:1 mixture of rotamers) δ 8.05 (d, 0.5H, J = 7.6 Hz), 7.80 (d, 0.5H, J = 7.6 Hz), 7.69 (d, 0.5H, J = 7.6 Hz), 7.22 (t, 1H, J = 7.6 Hz), 7.11 – 7.03 (m, 1.5H), 4.59 (d, 0.5H, J = 4.0 Hz), 4.16 (dd, 0.5H, J = 10.0, 2.8 Hz), 4.07 – 4.01 (m, 2H), 3.66 (s, 1.5H), 3.63 (s, 1.5H), 3.25 (m, 1H), 2.76 – 2.71 (m, 0.5H), 2.59 – 2.54 (m, 0.5H), 2.43 (s, 1.5H), 2.42 – 2.34 (m, 2H), 2.33 (s, 1.5H), 2.302 (s, 1.5H), 2.296 (s, 1.5H), 2.145 (s, 1.5H), 2.142 (s, 1.5H), 1.97 – 1.87 (m, 2H), 1.82 – 1.70 (m, 3H) ppm; **^{13}C NMR** ($CDCl_3$, 150 MHz) δ 168.9, 168.6, 168.2, 168.0, 162.9, 161.7, 141.6, 140.9, 139.2, 137.3, 127.9, 127.7, 127.2, 125.9, 124.6, 124.1, 118.6, 115.3, 107.0, 106.6, 68.2, 67.7, 67.4, 66.0, 63.8, 63.7, 53.9, 53.3, 53.2, 52.2, 51.3, 51.2, 41.0, 40.9, 40.0, 39.2, 38.0, 37.9, 30.3, 28.9, 28.2, 27.8, 24.2, 23.8, 20.2, 19.8 ppm; **IR** (thin film, cm^{-1}) 2944, 1704, 1652, 1475, 1392, 1241, 1105, 1090, 1077, 731; **HRMS** (+NSI) calculated for $C_{23}H_{29}N_2O_4$ $[M+H]^+$ 397.2122, found 397.2123.

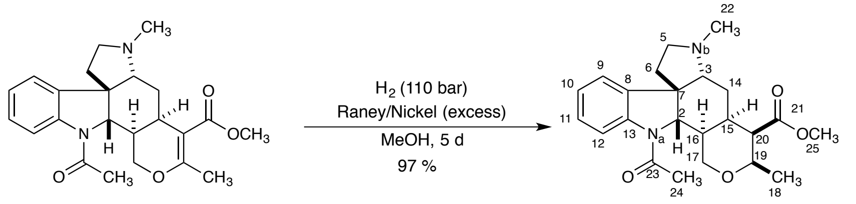

28 **(±)-malagashanine (1)**

Malagashanine 1:

MeOH (3.0 mL) was added to a vial charged with methylamine **28** (6.0 mg, 0.015 mmol) and Raney nickel (excess). The heterogeneous mixture was then submitted to hydrogen gas (110 bar) for 5 days in a Parr high-pressure vessel. The pressure was carefully released, and the mixture was filtered through celite. The filter cake was washed with MeOH (3 x 10.0 mL). The filtrate was concentrated under reduced pressure, and then EtOAc (15.0 mL) was added. The solution was washed with brine (3 x 10.0 mL), dried over anhydrous Na_2SO_4, filtered, and concentrated under reduced pressure. Purification by short flash column chromatography on silica gel (93:7 CH_2Cl_2/methanol with 0.5% $NH_4OH_{(aq)}$) afforded malagashanine as a white amorphous solid (5.8 mg, 97 %).

R_f 0.25 (9:1 $CHCl_3$/methanol); **^{1}H NMR** ($CDCl_3$, 600 MHz) δ 8.00 (d, 1H, J = 7.8 Hz), 7.69 (d, 1H, J = 7.8 Hz), 7.19 (t, 1H, J = 7.8 Hz), 7.02 (t, 1H, J = 7.8 Hz), 4.73 (d, 1H, J = 9.0 Hz), 4.11 (dd, 1H, J = 12.6, 1.8 Hz), 3.72 (s, 3H), 3.65 (qd, 1H, J = 6.6, 3.0 Hz), 3.48 (dd, 1H, J = 12.6, 3.6 Hz), 3.22 (td, 1H, J = 8.4, 1.8 Hz), 2.52 (dd, 1H, J = 5.4, 3.0 Hz), 2.41 (s, 3H), 2.33 (s, 3H), 2.32 – 2.25 (m, 3H), 1.94 – 1.86 (m, 2H), 1.72 (td, 1H, J =

13.2, 6.6 Hz), 1.62 – 1.60 (m, 1H), 1.48 – 1.47 (m, 1H), 1.23 (d, 3H, *J* = 6.6 Hz) ppm; **^{13}C NMR** ($CDCl_3$, 150 MHz) δ 174.4, 168.7, 141.0, 138.1, 127.5, 126.0, 124.3, 119.5, 75.0, 68.4, 66.4, 64.6, 55.2, 52.9, 51.8, 48.6, 41.5, 38.7, 37.0, 36.3, 28.3, 24.1, 18.5 ppm; **IR** (thin film, cm^{-1}) 2928, 2851, 1731, 1655, 1460, 1396, 1161, 1106, 1033, 757; **HRMS** (+NSI) calculated for $C_{23}H_{31}N_2O_4$ $[M+H]^+$ 399.2278, found 399.2278.

The NMR spectra of synthetic malagashanine showed a set of major peaks with a set of minor peaks (major peaks : minor peaks = 5:1). These are assigned as acetamide rotamers (VT ^{1}H NMR shows broadening, but not full coalescence at 90 °C). The major peaks were compared with the data from the isolation paper (Table 2.1 for ^{1}H NMR data comparison, and Table 2.2 for ^{13}C NMR data comparison).

Table 2.1 ^{1}H NMR data comparison for malagashanine

Position	Literature (500 MHz)	Synthetic (600 MHz)	Difference
2	4.70, d, *J* = 8.5	4.73 (d, 1H, *J* = 9.0 Hz)	0.03
3	2.24, m	2.32 – 2.25 (m, 1H)	/
5	3.22, ddd, *J* = 9.9, 8.1, 1.9	3.22 (td, 1H, *J* = 8.4, 1.8 Hz)	0
	2.28, m	2.32 – 2.25 (m, 1H)	/
6	1.84, m	1.94 – 1.86 (m, 1H)	/
	1.56, ddd, *J* = 8.6, 8.1, 2.7	1.62 – 1.60 (m, 1H)	/
7			
8			
9	7.59, br d, *J* = 8.0	7.69 (d, 1H, *J* = 7.8 Hz)	0.1
10	7.07, br dd, *J* = 8.0, 8.0	7.02 (t, 1H, *J* = 7.8 Hz)	- 0.05
11	7.15, br dd, *J* = 8.0, 8.0	7.19 (t, 1H, *J* = 7.8 Hz)	0.04
12	7.95, br d, *J* = 8.0	8.00 (d, 1H, *J* = 7.8 Hz)	0.05
13			
14	1.70, ddd, *J* = 3.4, 6.6, 6.6	1.72 (td, 1H, *J* = 13.2, 6.6 Hz)	0.02
	1.92, m	1.94 – 1.86 (m, 1H)	/
15	2.26, m	2.32 – 2.25 (m, 1H)	/
16	1.43, m	1.48 – 1.47 (m, 1H)	/
17	3.41, dd, *J* = 2.5, 3.8	3.48 (dd, 1H, *J* = 12.6, 3.6 Hz)	0.07
	4.05, dd, *J* = 2.5, 2.3	4.11 (dd, 1H, *J* = 12.6, 1.8 Hz)	0.06
18	1.19, 3H, d, *J* = 7.0	1.23 (d, 3H, *J* = 6.6 Hz)	0.04
19	3.60, dq, *J* = 7.0, 3.5	3.65 (qd, 1H, *J* = 6.6, 3.0 Hz)	0.05
20	2.49, dd, *J* = 5.7, 3.5	2.52 (dd, 1H, *J* = 5.4, 3.0 Hz)	0.03
21			
22	2.32, s, 3H	2.33 (s, 3H)	0.01
23			
24	2.36, s, 3H	2.41 (s, 3H)	0.05
25	3.67, s, 3H	3.72 (s, 3H)	0.05

Table 2.2 ^{13}C NMR data comparison for malagashanine

Position	Literature (125 MHz)	Synthetic (100 MHz)	Difference	Difference-0.2
2	66.3	66.4	0.1	- 0.1
3	64.3	64.6	0.3	0.1
5	52.5	52.9	0.4	0.2
6	36.8	37.0	0.2	0
7	55.0	55.2	0.2	0
8	137.9	138.1	0.2	0
9	125.8	126.0	0.2	0
10	124.1	124.3	0.2	0
11	127.3	127.5	0.2	0
12	119.3	119.5	0.2	0
13	140.8	141.0	0.2	0
14	28.1	28.3	0.2	0
15	36.0	36.3	0.3	0.1
16	38.6	38.7	0.1	- 0.1
17	68.2	68.4	0.2	0
18	18.3	18.5	0.2	0
19	74.8	75.0	0.2	0
20	48.4	48.6	0.2	0
21	174.2	174.4	0.2	0
22	41.2	41.5	0.3	0.1
23	168.5	168.7	0.2	0
24	23.9	24.1	0.2	0
25	51.6	51.8	0.2	0

The ^{13}C NMR data of our synthetic sample are in excellent agreement with those from the isolation paper (Table 2.2).[1]

The ^{1}H NMR data also matched well with the data reported in the isolation paper. Of particular importance, the data characterizing the *syn* relationship between C(19) and C(20) matched well with the reported data.[1] Moreover, the small coupling constant (3.0 Hz) between C(19) and C(20) protons was expected for this type of J^3 coupling because the dihedral angle was close to 90°. However, there were three discrepancies between the data of our synthetic sample and those from the isolation paper. We believe that they are most likely the result of typographical errors in the original paper. The first major inconsistency was the coupling constant for the proton on C(14). The reported data contained a coupling constant of 3.4 Hz while our data contained a coupling constant of 13.2 Hz. This proton is expected to have a J^2 coupling, which is usually larger than 12 Hz (based on dihedral angles from the crystal structure).[2] Therefore, we believe the 3.4 Hz reported from the isolation paper was a typographical error. (We speculate, it might be 13.4 Hz, with the "1" being accidently omitted). The other two inconsistencies were the coupling constants for the two protons on C(17). The reported data contained coupling constants of 2.5 Hz for both protons while our data contained coupling constants of 12.6 Hz. We believe that these discrepancies were caused by the same type of mistake as the proton on C(14) because these protons should also have a large J^2 coupling constants

(based on dihedral angles from the crystal structure). In support of the hypothesis that these are likely to be typographical errors, coupling constants are usually reported in descending order. All the other coupling constants reported in the isolation manuscript adhere to this standard formatting. However, these three coupling constants in question are outliers, and do not match this standard convention. On the basis of the analysis above, we conclude that the synthetic sample is identical to the natural product malagashanine.

1. (a) Rasoanaivo, P.; Ratsimamanga-Urverg, S.; Milijaona, R.; Rafatro, H.; Rakoto-Ratsimamanga, A.; Galeffi, C.; Nicoletti, M. *Planta Med.* **1994,** *60*, 13. (b) Rasoanaivo, P.; Galeffi, C.; Palazzino, G.; Nicoletti, M. *Gazzeta Chimica Italiana* **1996,** *126*, 517.
2. Caira, M. R.; Rasoanaivo, P. *J. Chem. Crystallogr.* **1995,** *25*, 725.

III. Structural assignments for 14 and 26

Key ^{1}H NMR and NOESY assignments for 14

^{1}H-NMR

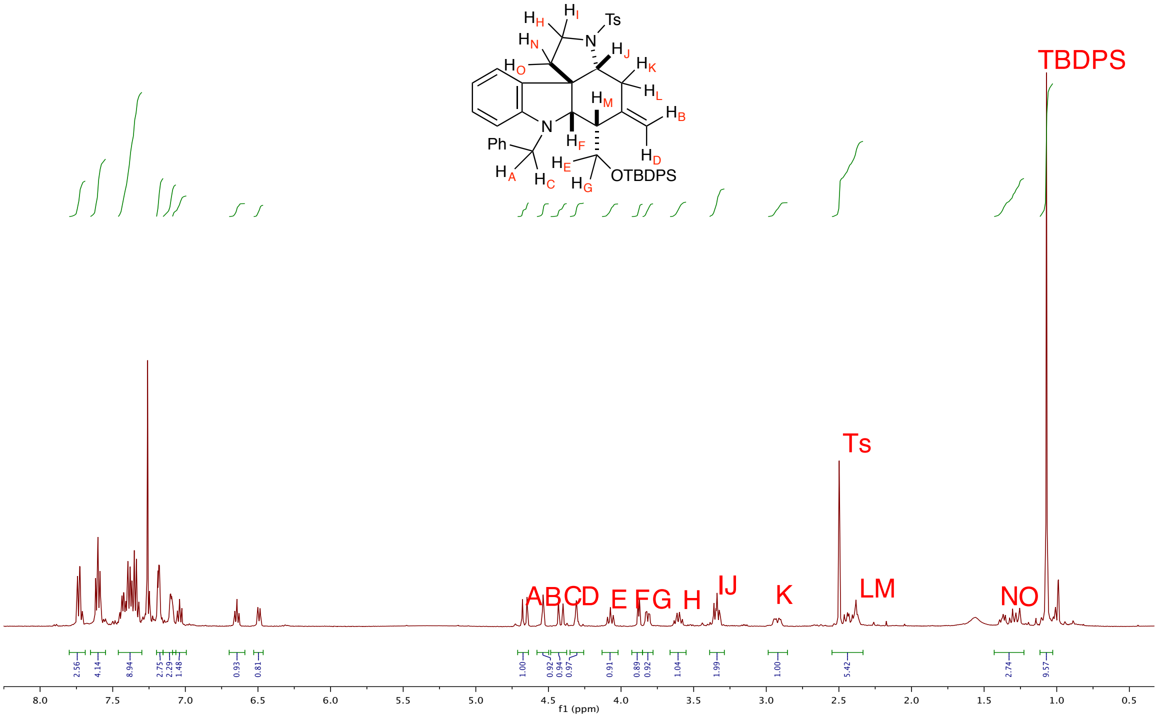

^{1}H-COSY

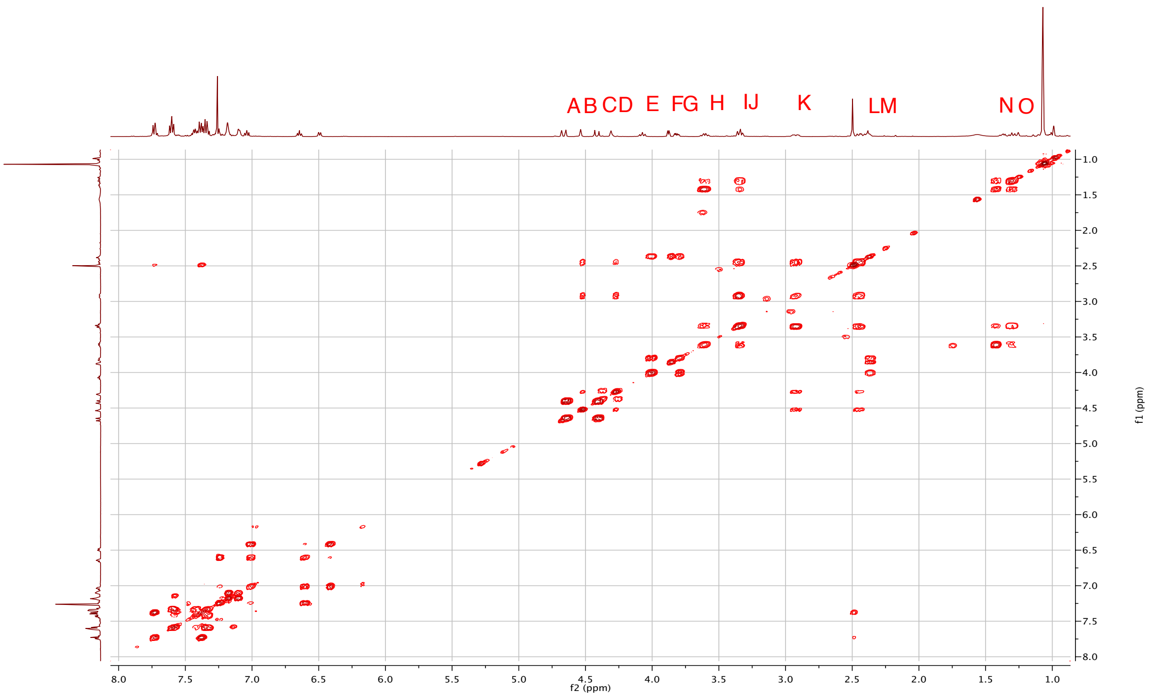

^{1}H-NOESY

Key nOe Correlations

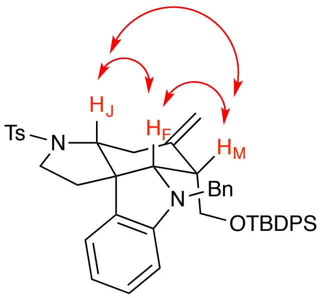

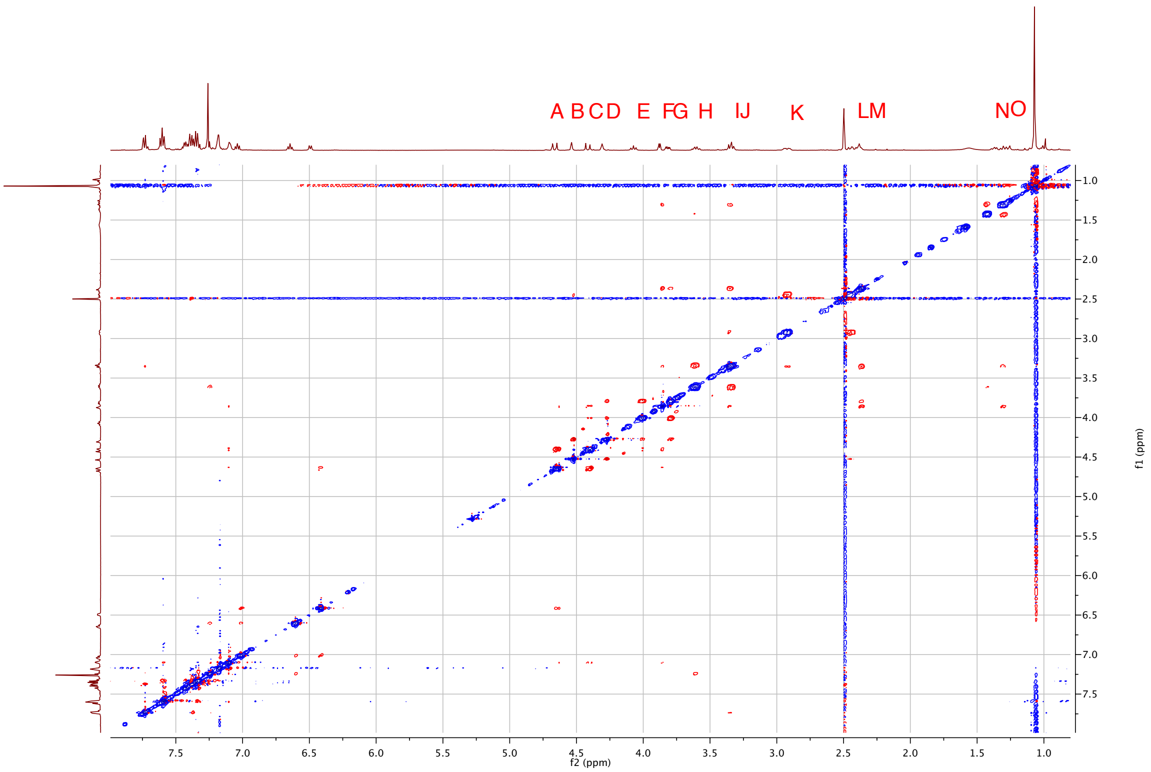

Structure of **26** assigned by x-ray crystallography (CCDC 1489617)

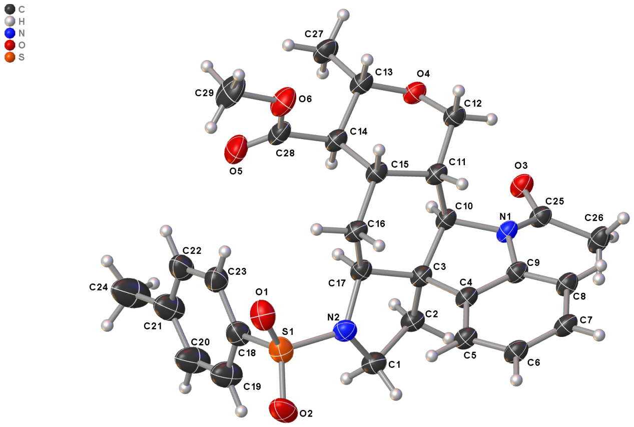

Crystal data and structure refinement for dm-ii-253_sq.

Identification code	dm-ii-253_sq
Empirical formula	C29H34N2O6S
Formula weight	538.64
Temperature/K	173(2)
Crystal system	tetragonal
Space group	I41/a
a/Å	29.2891(19)
b/Å	29.2891(19)
c/Å	18.1917(13)
α/°	90
β/°	90
γ/°	90
Volume/Å3	15606(2)
Z	16
ρcalcg/cm3	0.917
μ/mm□1	1.002
F(000)	4576.0
Crystal size/mm3	0.36 × 0.23 × 0.11
Radiation	CuKα (λ = 1.54178)
2Θ range for data collection/°	5.718 to 130.156
Index ranges	-32 ≤ h ≤ 31, -33 ≤ k ≤ 29, -21 ≤ l ≤ 21
Reflections collected	37725
Independent reflections	6404 [Rint = 0.1158, Rsigma = 0.0828]
Data/restraints/parameters	6404/249/347
Goodness-of-fit on F2	1.014
Final R indexes [I>=2σ (I)]	R1 = 0.0690, wR2 = 0.1637
Final R indexes [all data]	R1 = 0.1707, wR2 = 0.2190
Largest diff. peak/hole / e Å-3	0.17/-0.18

IV. Attempted reductions of 28

Table 4.1. Homogenous hydrogenation of N_b-methyl ester **28**

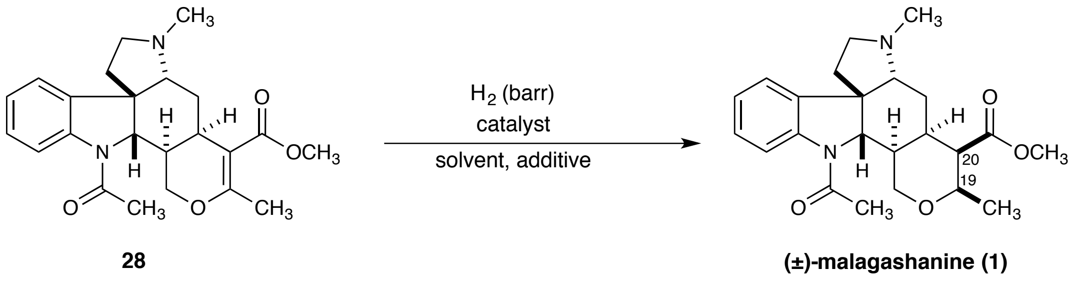

entry	catalyst	H_2 (barr)	solvent	additive	time	result[b]
1	Crabtree's (1.0 equiv.)	100	CH_2Cl_2	/	15 hr	NR
2	[Rh]/Josiphos (2.0 equiv.)	100	MeOH/EtOAc	/	2 d	NR
3	[Rh]/Josiphos (2.0 equiv.)	100	MeOH/EtOAc	$Zn(OTf)_2$	2 d	NR
4	[Ir]BAr_F (1.0 equiv.)	100	CH_2Cl_2	/	20 hr	NR
5	[Ir]BAr_F (0.1 equiv.)	100	CH_2Cl_2	/	20 hr	NR
6	[Ir]BAr_F (10.0 equiv.)	50	CH_2Cl_2	/	4 d	NR
7	[Ir]BAr_F (10.0 equiv.)	50	CH_2Cl_2	PTSA	4 d	NR

[a] reaction conditions: substrate (0.3 mg), catalyst, solvent and/or additives was added in a vial and put in a Parr autoclave under hydrogen. [b] detected by LC-MS.

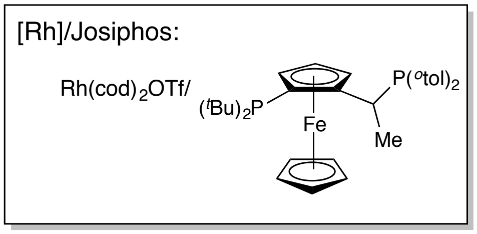

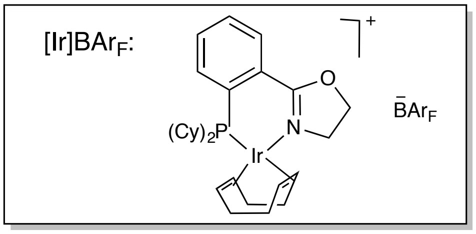

Table 4.2. Heterogeneous hydrogenation of N_b-methyl ester **28**

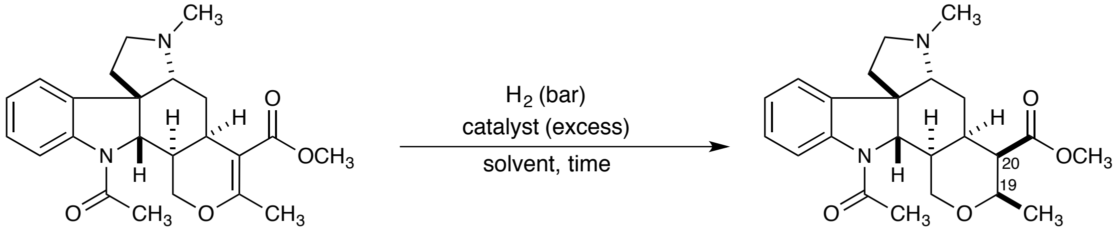

entry	catalyst	H_2 (bar)	solvent	time	result[b]
1	PtO_2	100	MeOH	2 d	NR
2	PtO_2	100	EtOAc	16 hr	trace
3	PtO_2	100	THF	3 d	trace
4	Rh/Al	100	MeOH	2 d	NR
5	Rh/Al	100	EtOAc	16 hr	trace[c]
6	Rh/Al	100	THF	3 d	some[c]
7	Rh/C	150	MeOH	3 d	trace
8	Rh/C	100	THF	3 d	benzene ring reduced[d]
9	Pd/C	100	MeOH	3 d	trace
10	Pd/C	100	THF	3 d	NR
11	Pt/C	150	MeOH	3 d	NR
12	Raney/Ni	100	MeOH	4 d	major[e]
**13**	**Raney/Ni**	**110**	**MeOH**	**5 d**	**complete conversion**

[a] reaction conditions: substrate (0.3 mg), catalyst, solvent and/or additives was added in a vial and put in a Parr autoclave under hydrogen. [b] detected by LC-MS. [c] trace benzene ring also been reduced. [d] LC-MS showed a peak with MS 6 more than MS(sub). [e] still had trace substrate.

V. NMR Spectra

PMB Ether S-1:

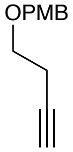

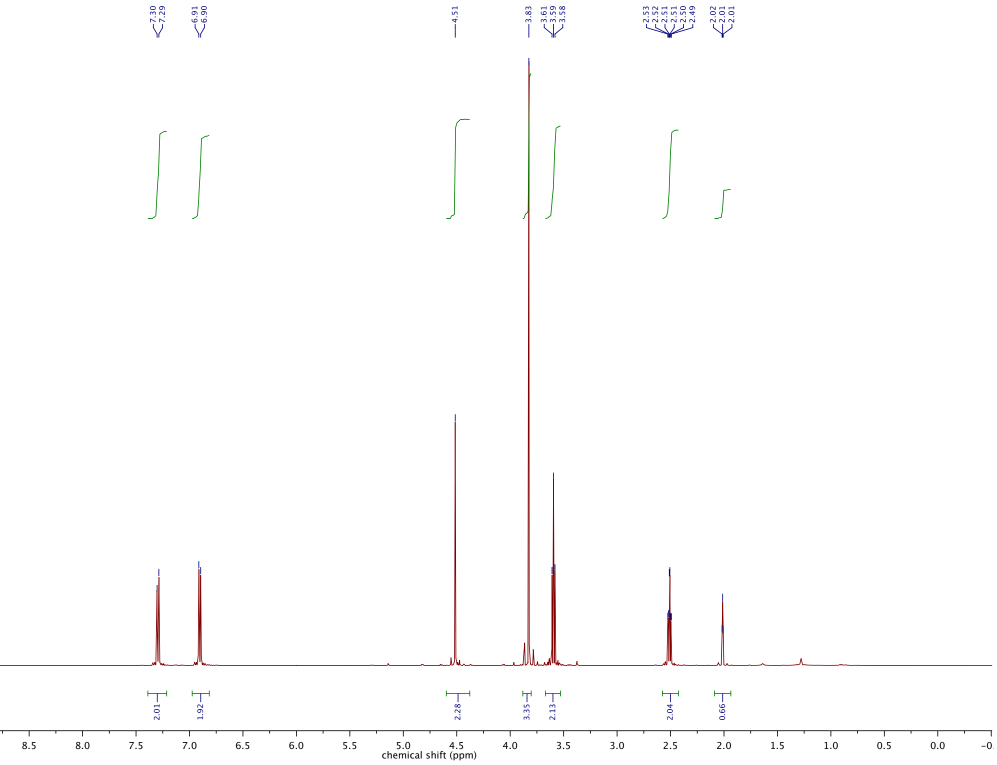

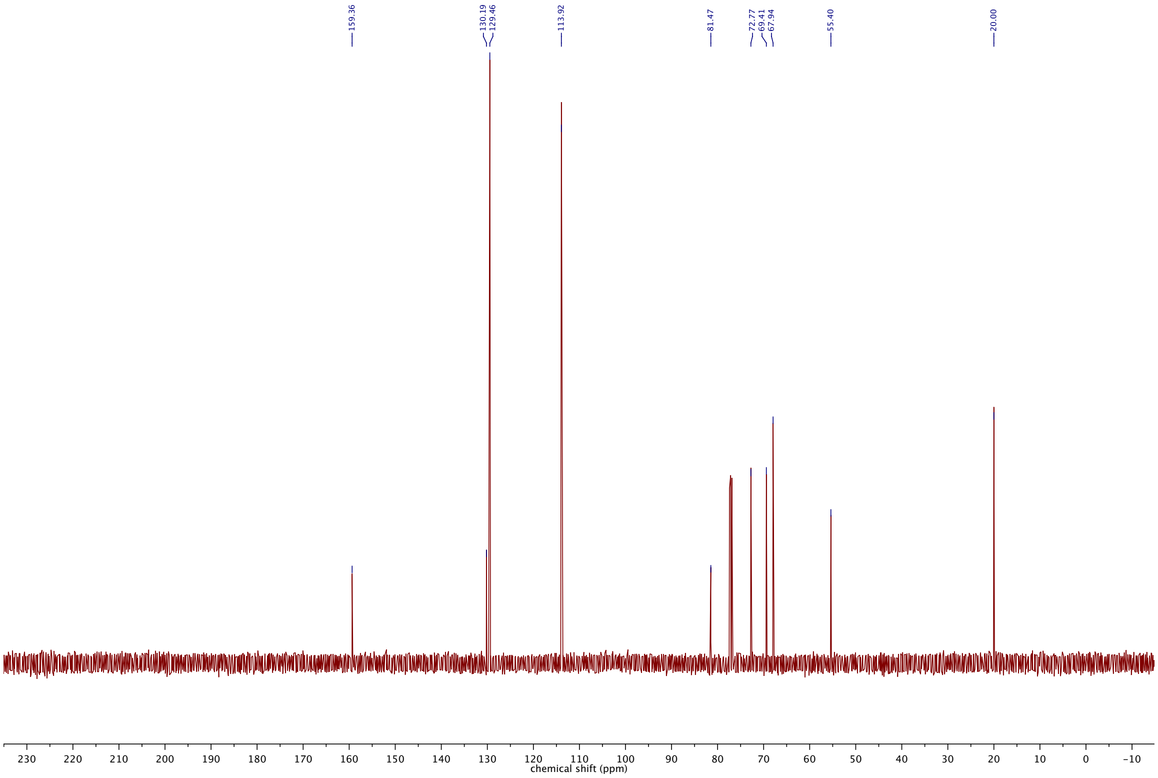

Propargyl Alcohol 10:

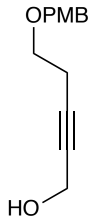

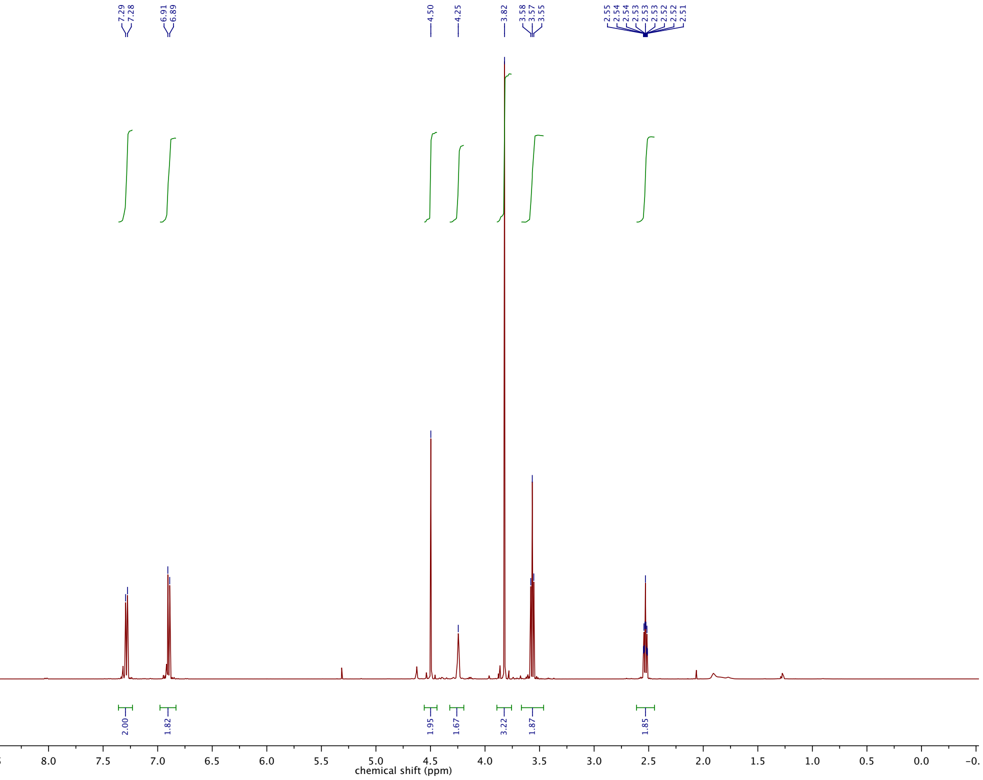

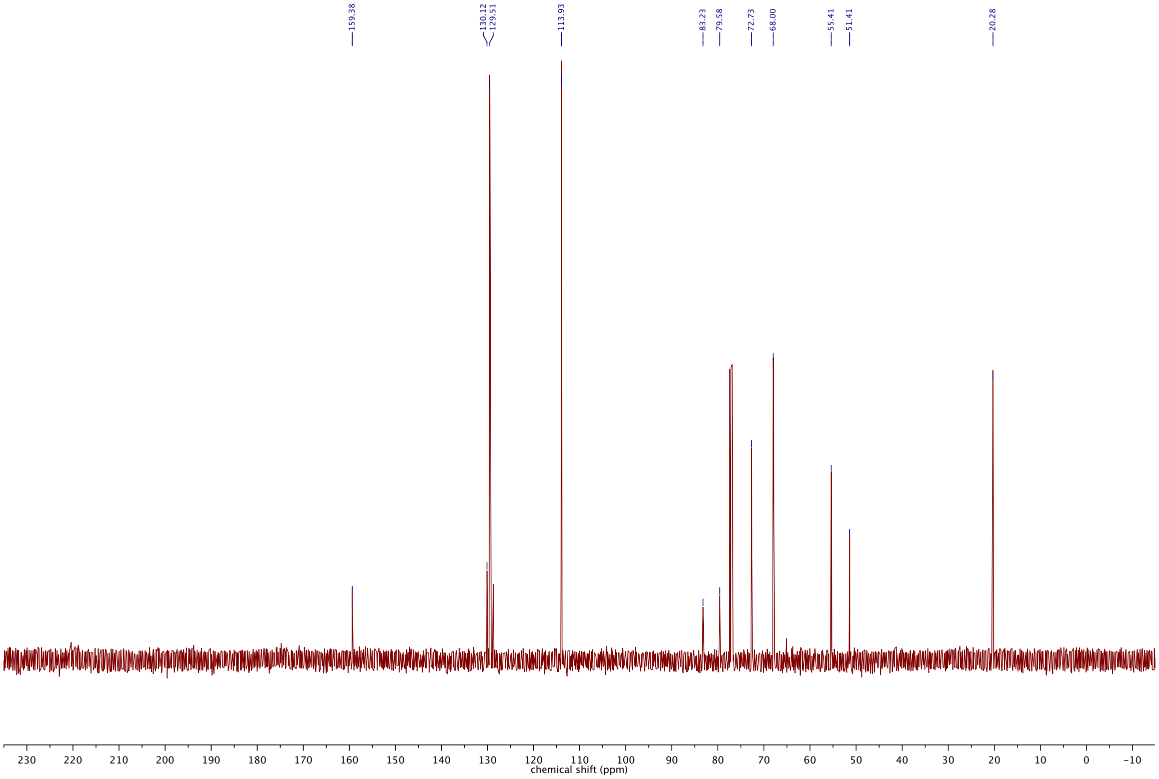

Vinyl Iodide 11:

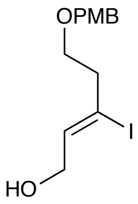

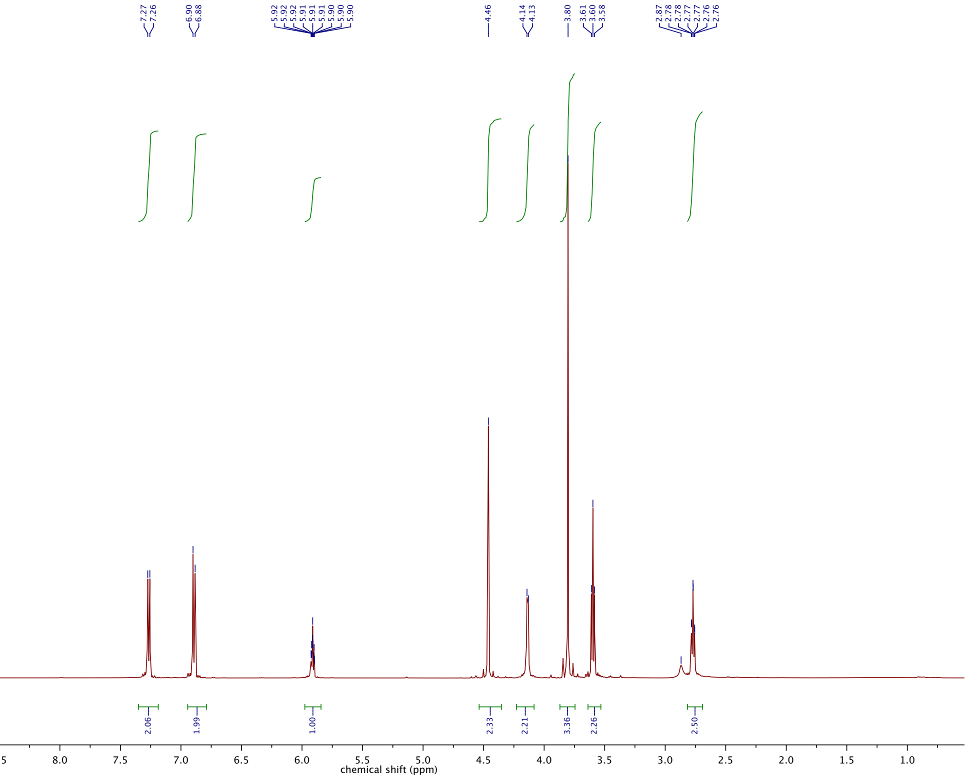

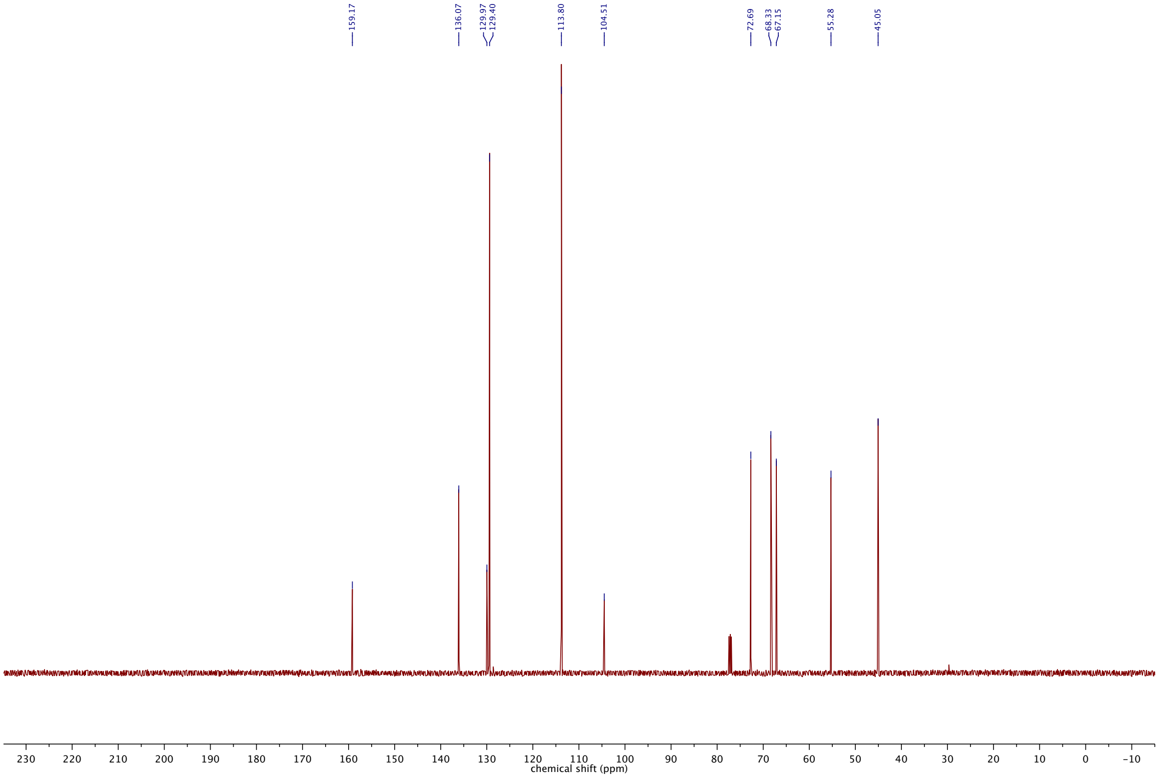

Alcohol 12:

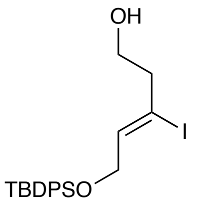

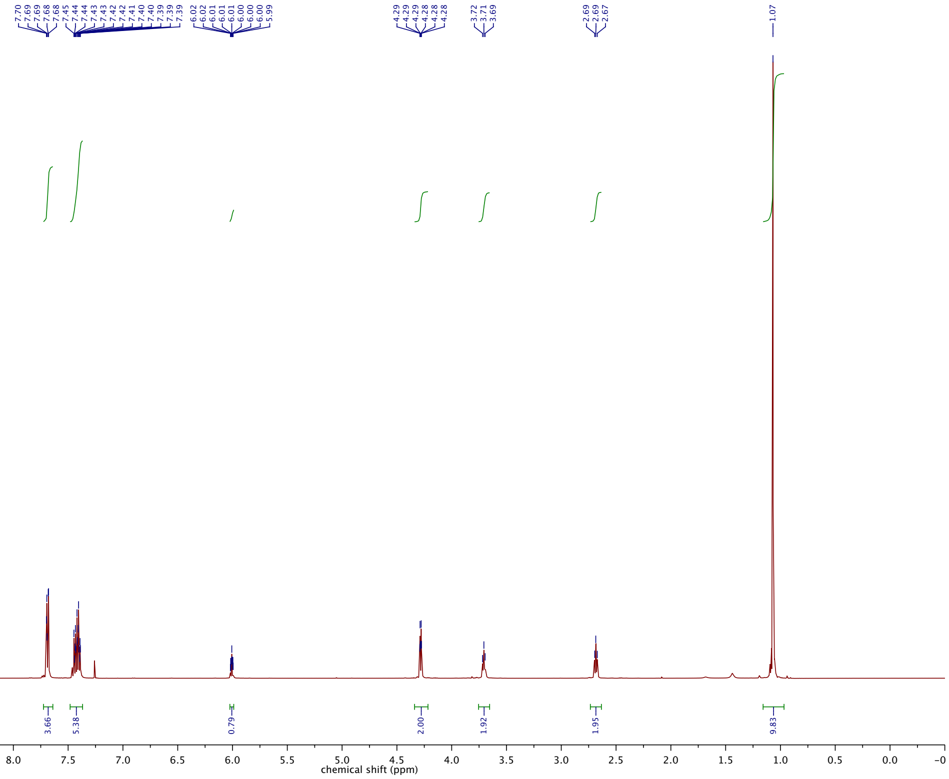

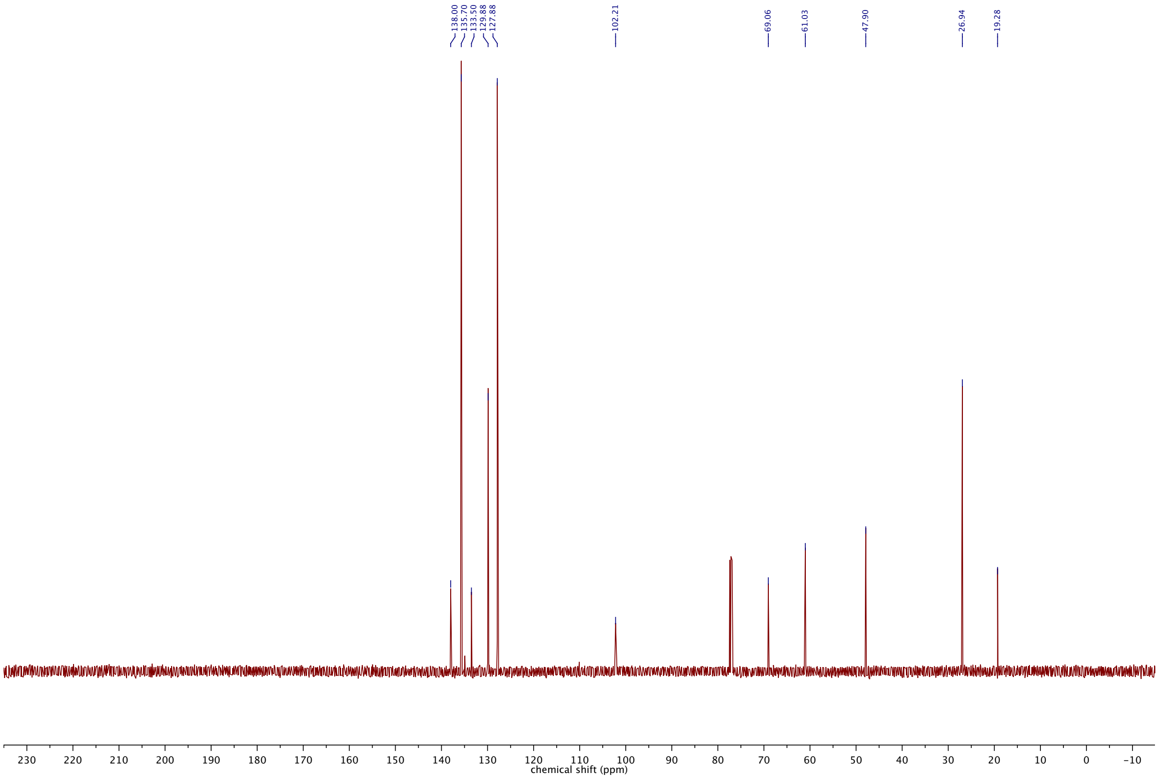

Allylsilane 9:

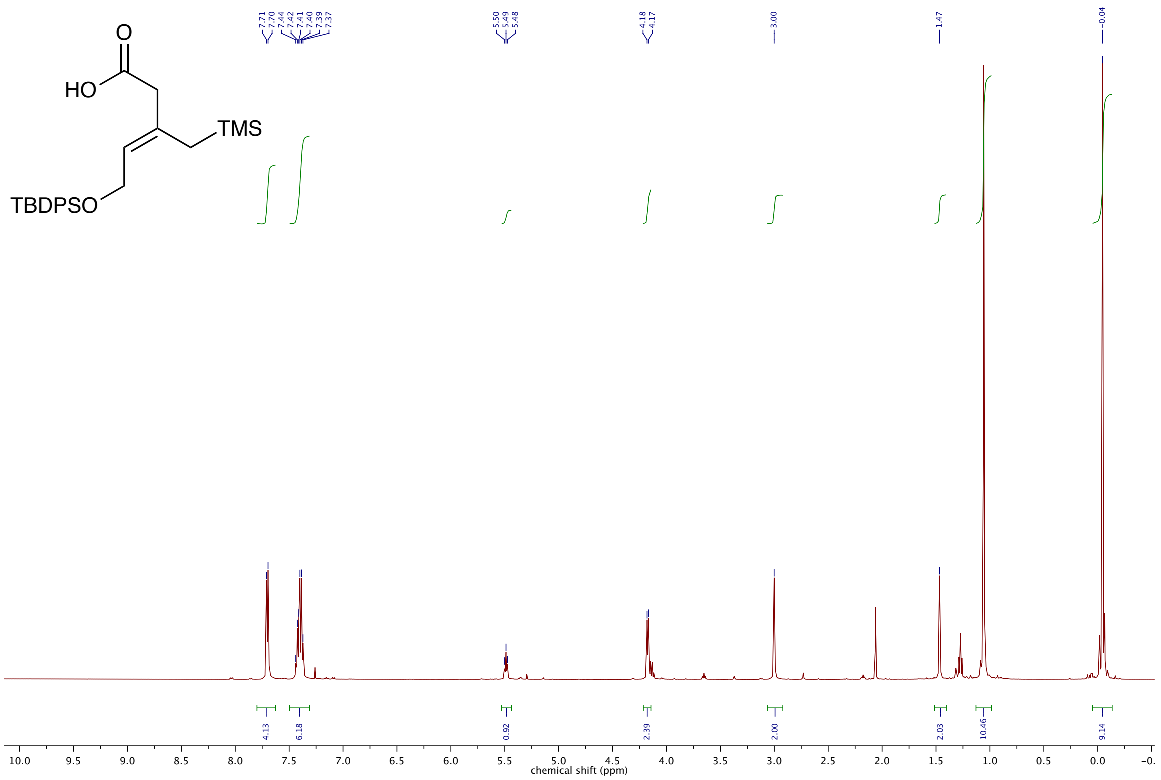

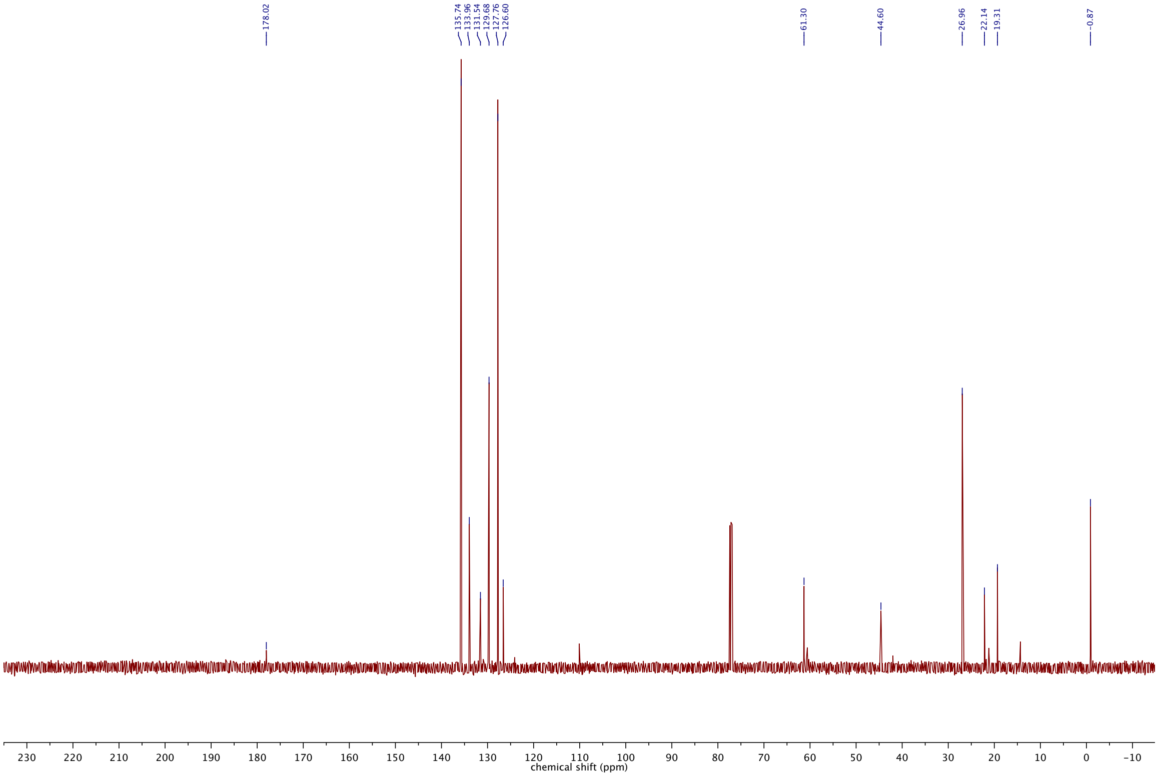

N-Tosylamide 13:

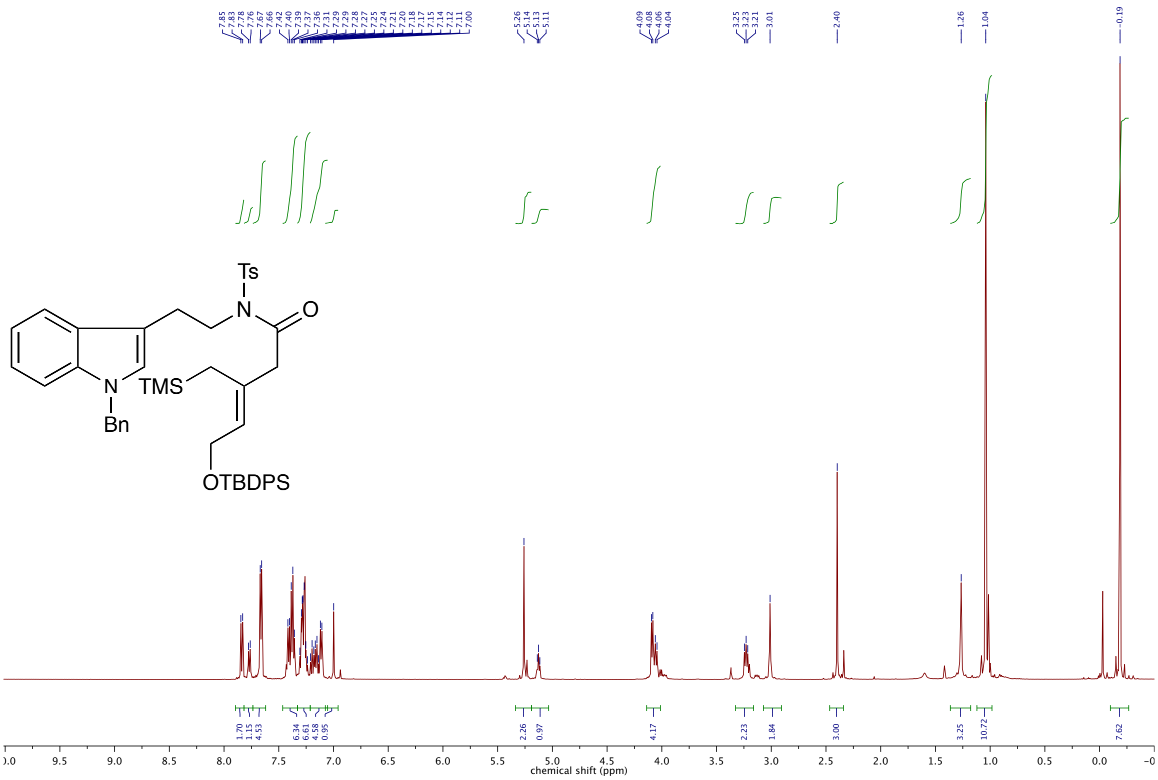

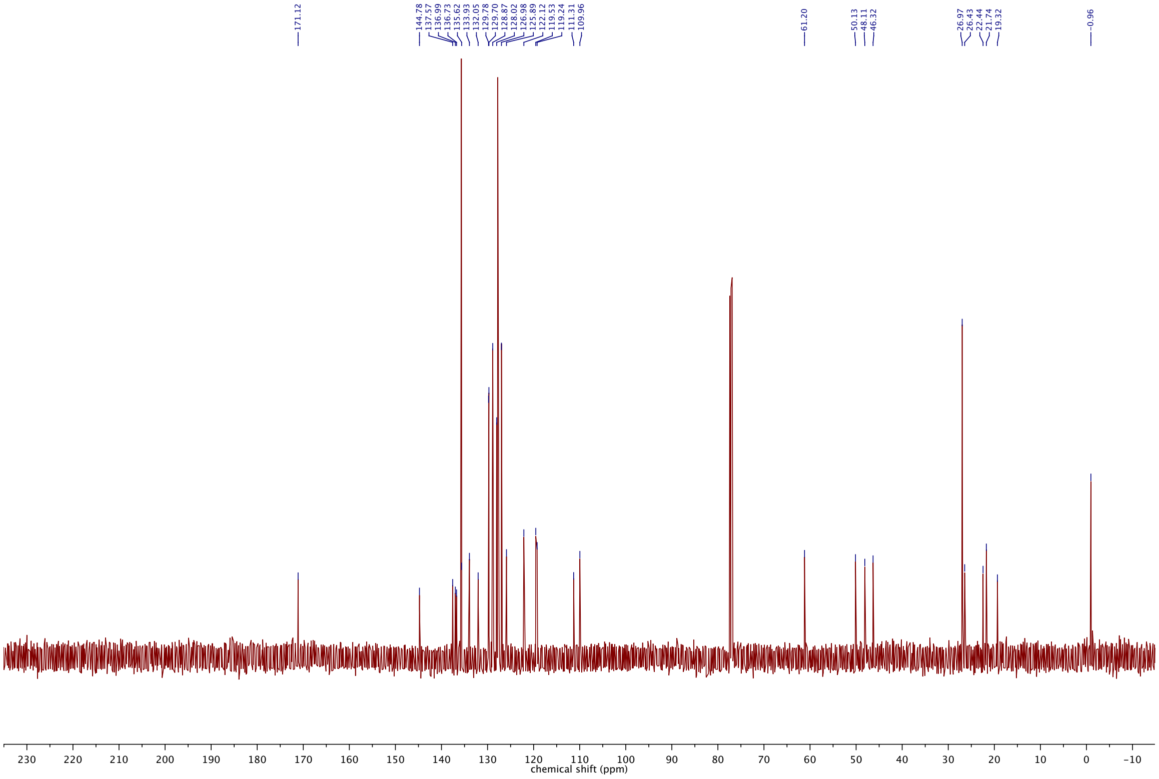

Hemiaminal S-6:

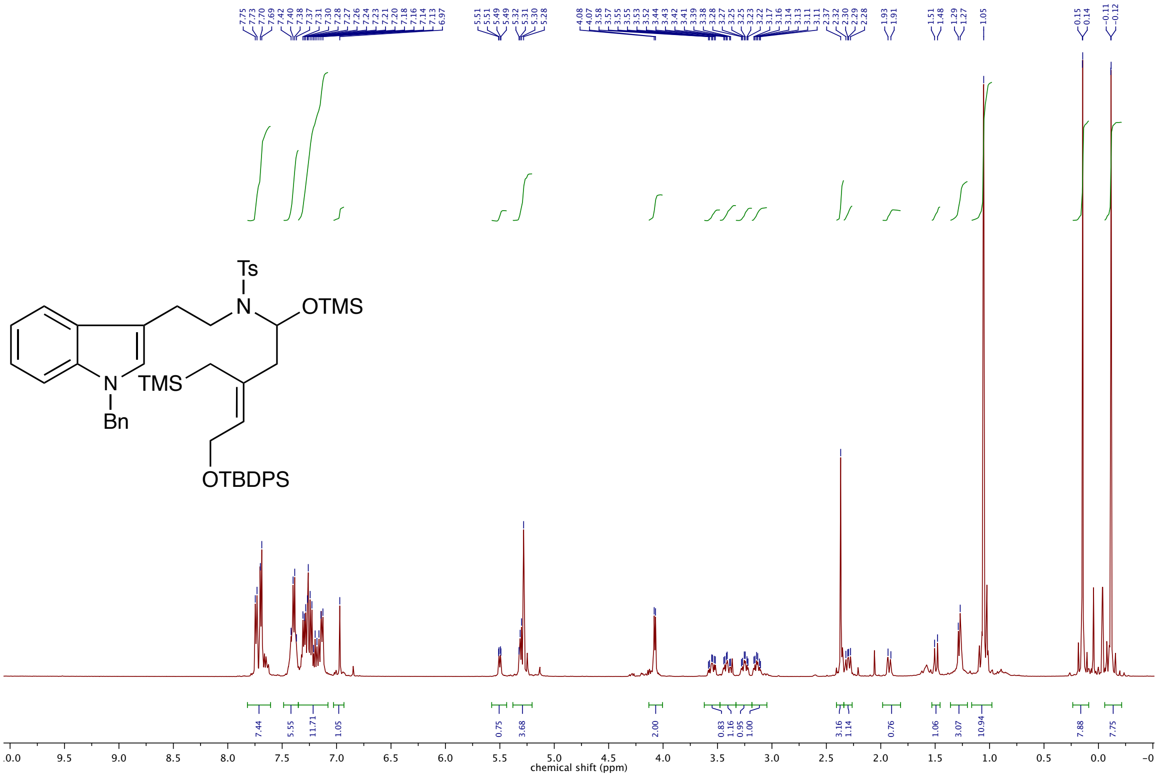

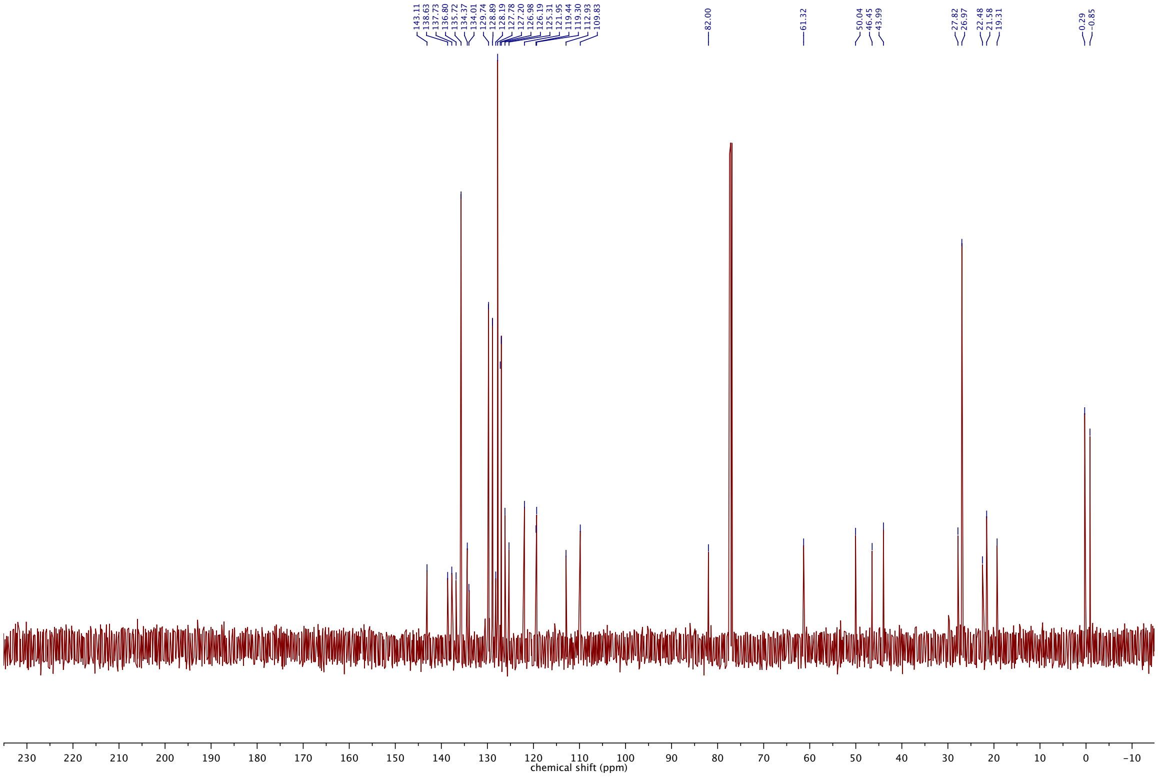

Tetracycle 14:

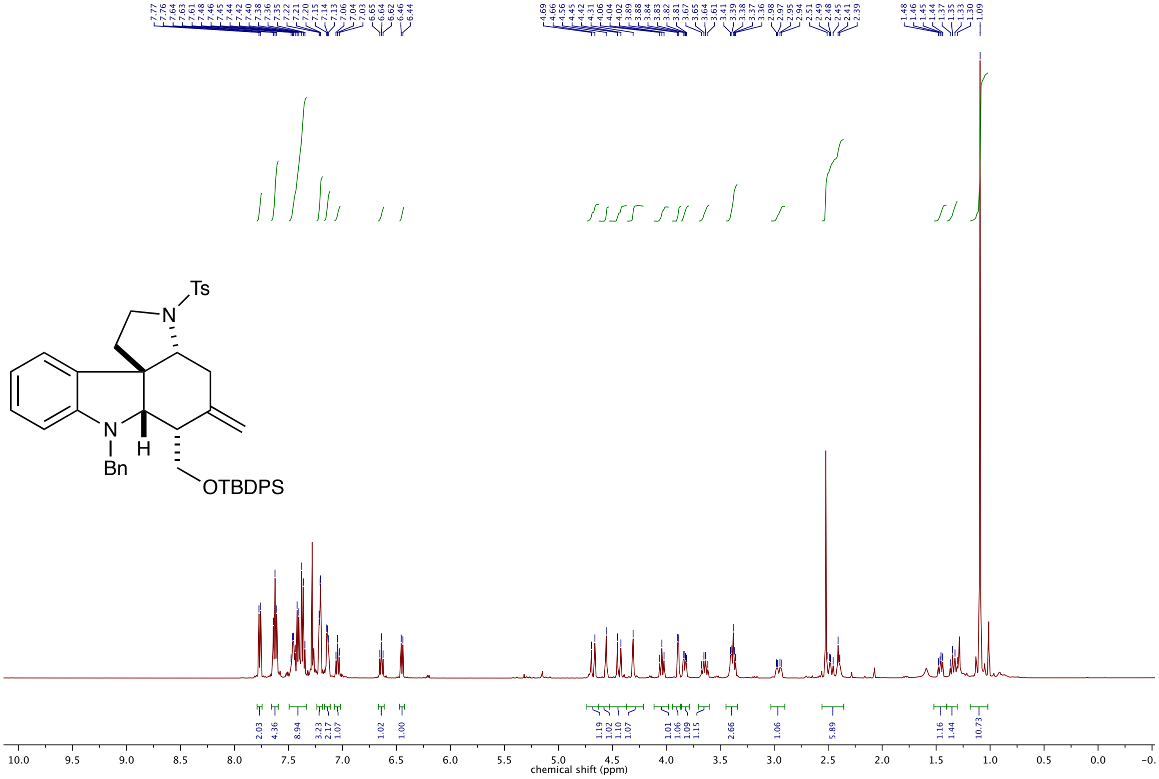

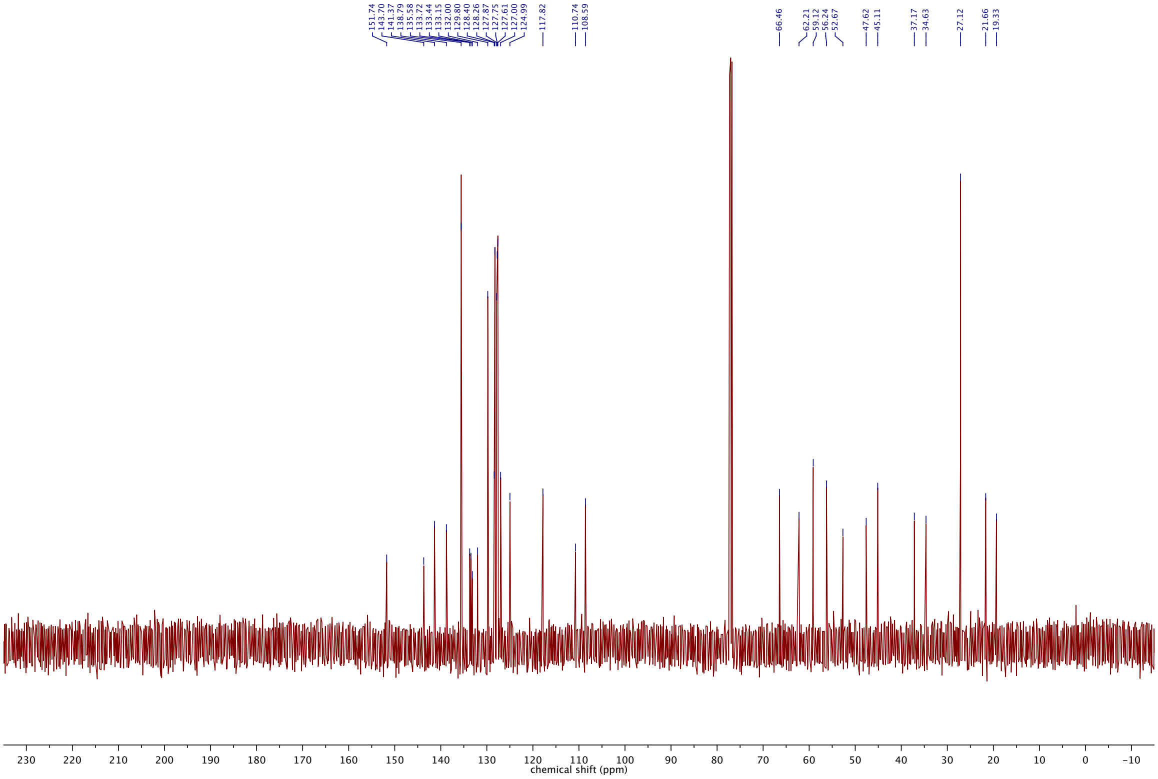

^{1}H-COSY

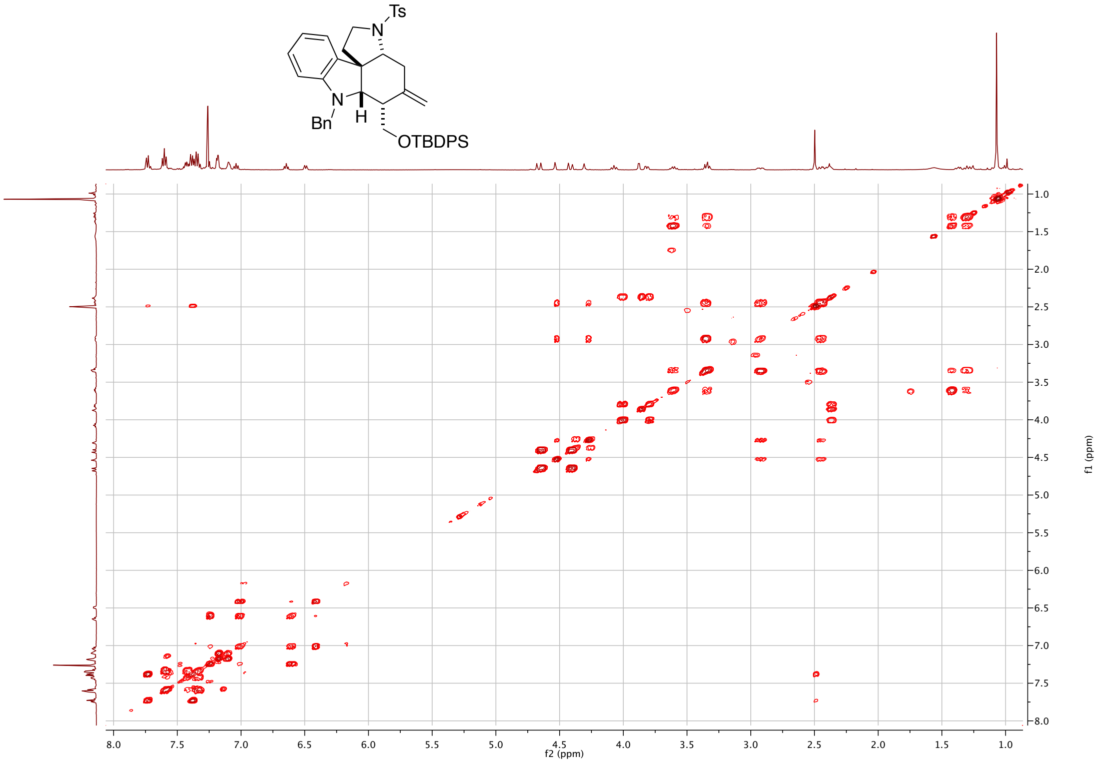

^{1}H-NOESY

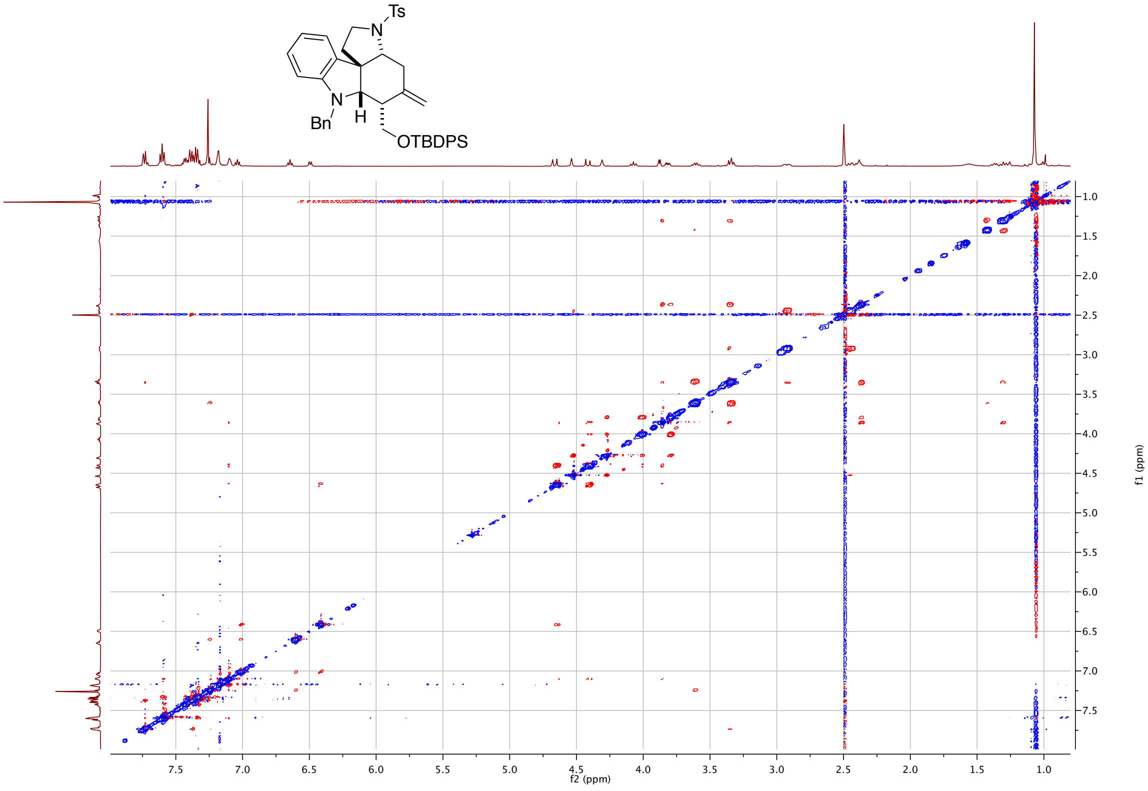

Homopropargylic Alcohol 17:

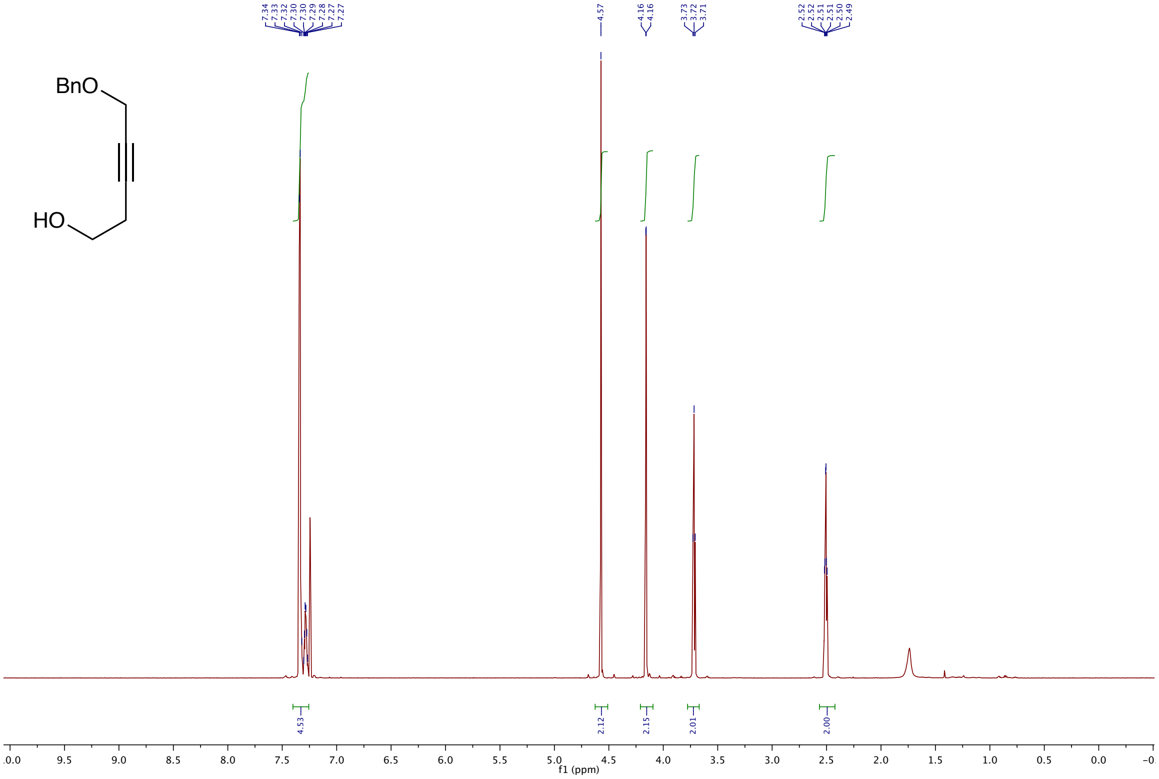

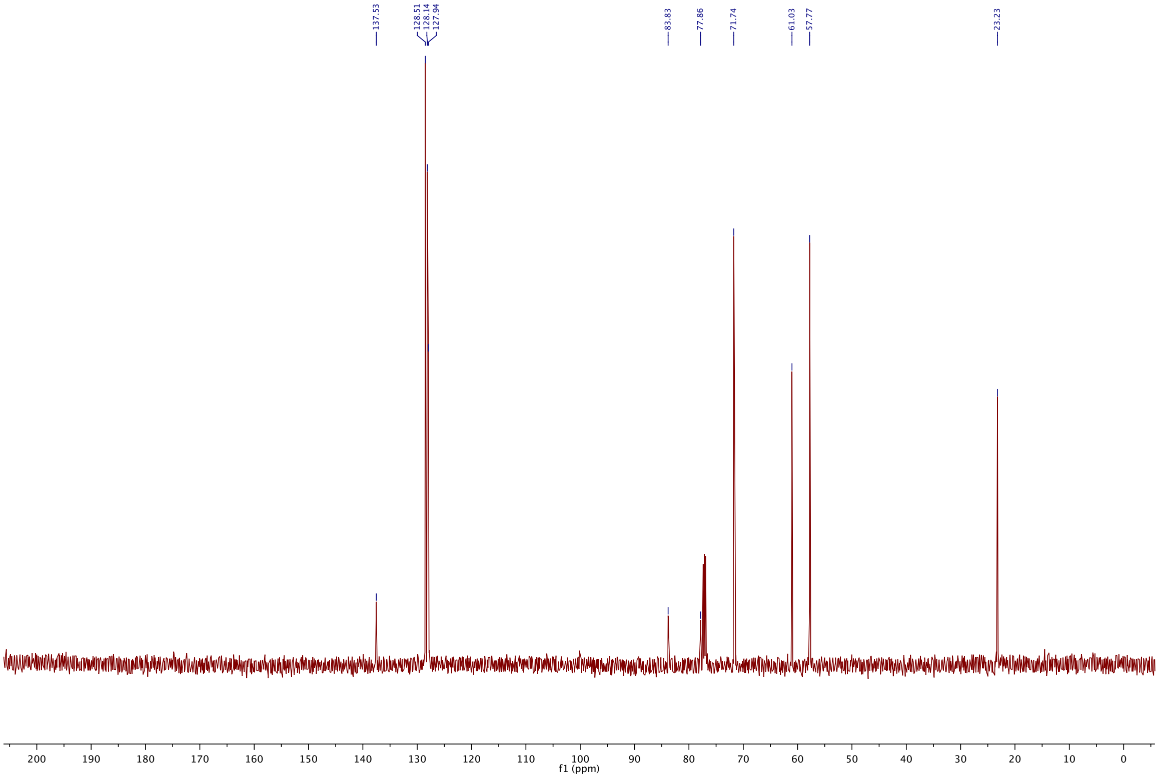

Vinyl Iodide 16:

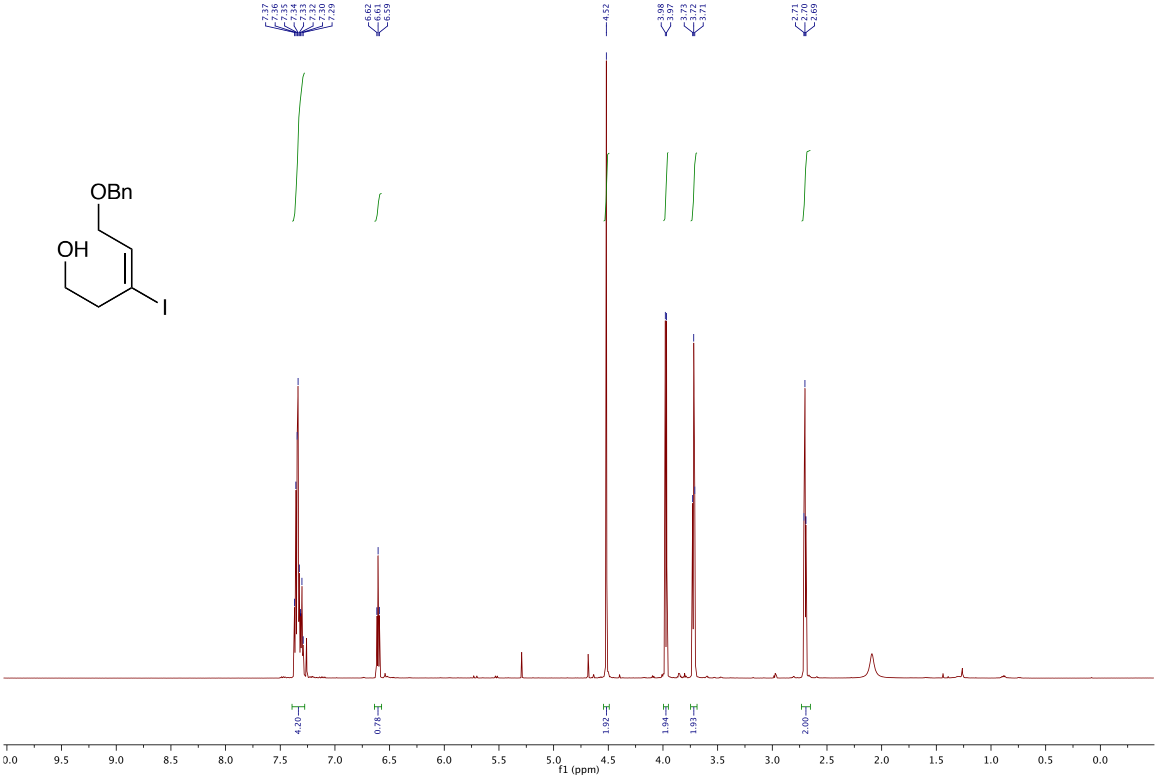

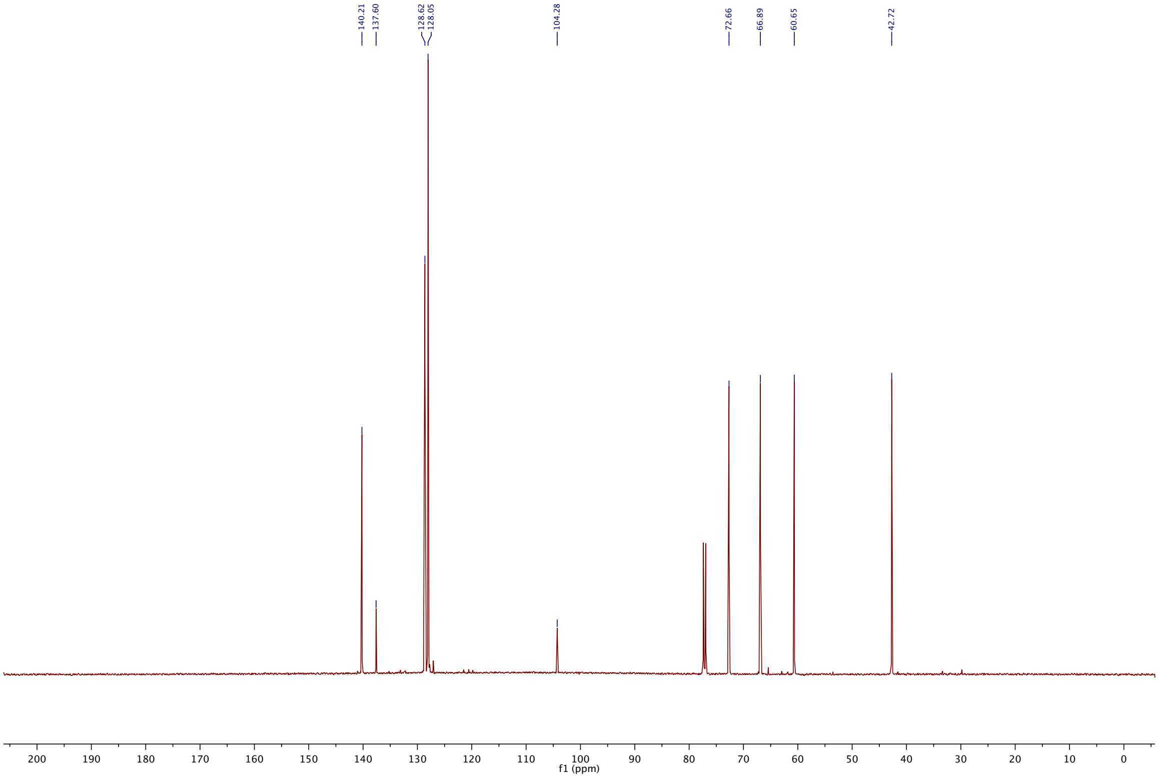

Allylsilane 15:

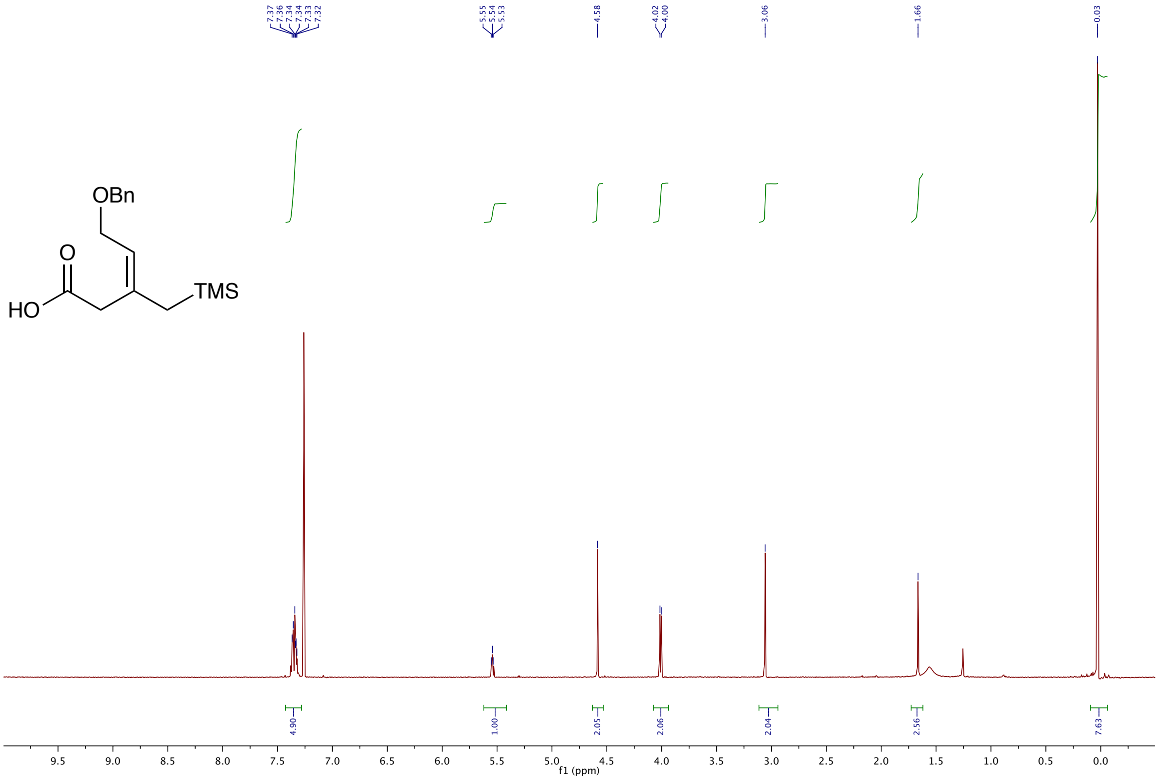

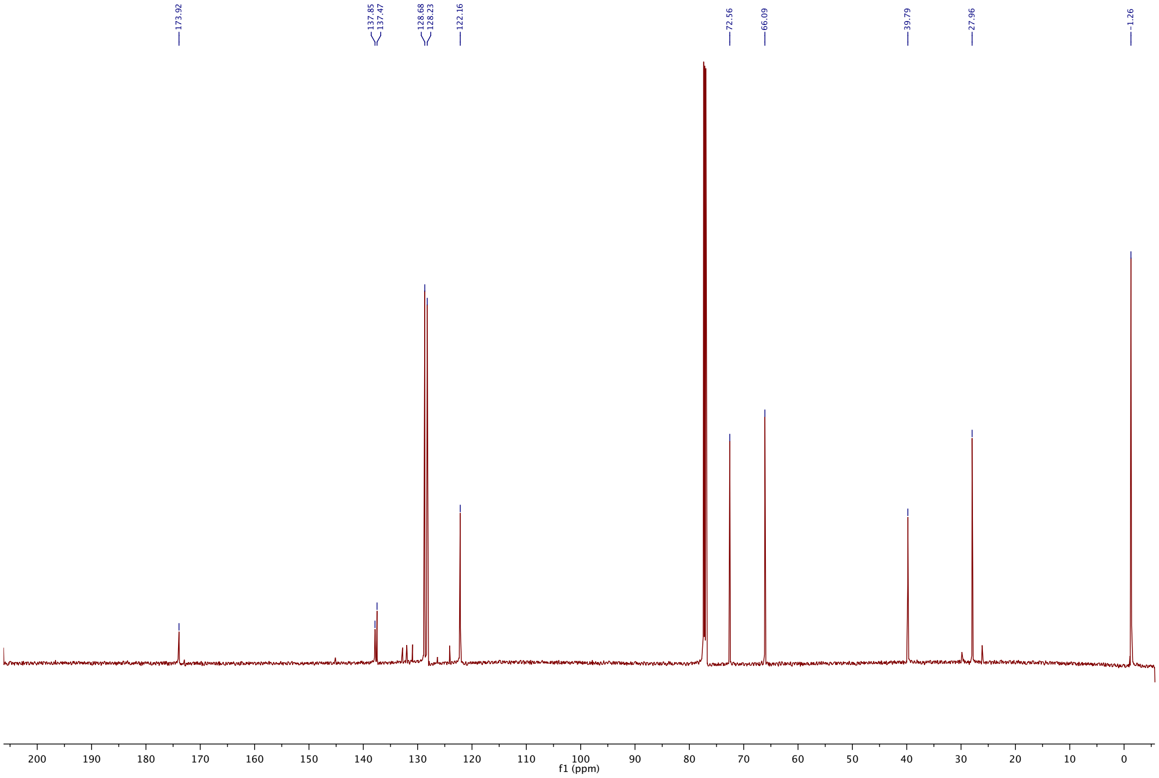

N-Tosylamide 19:

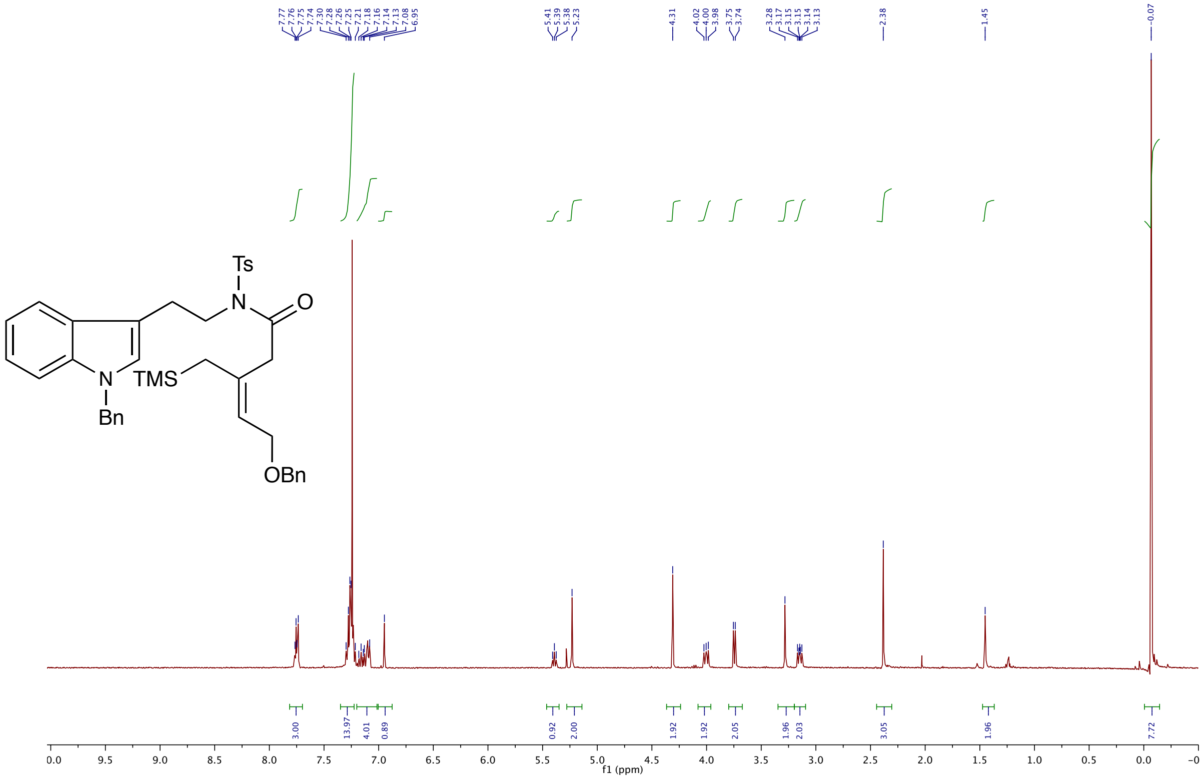

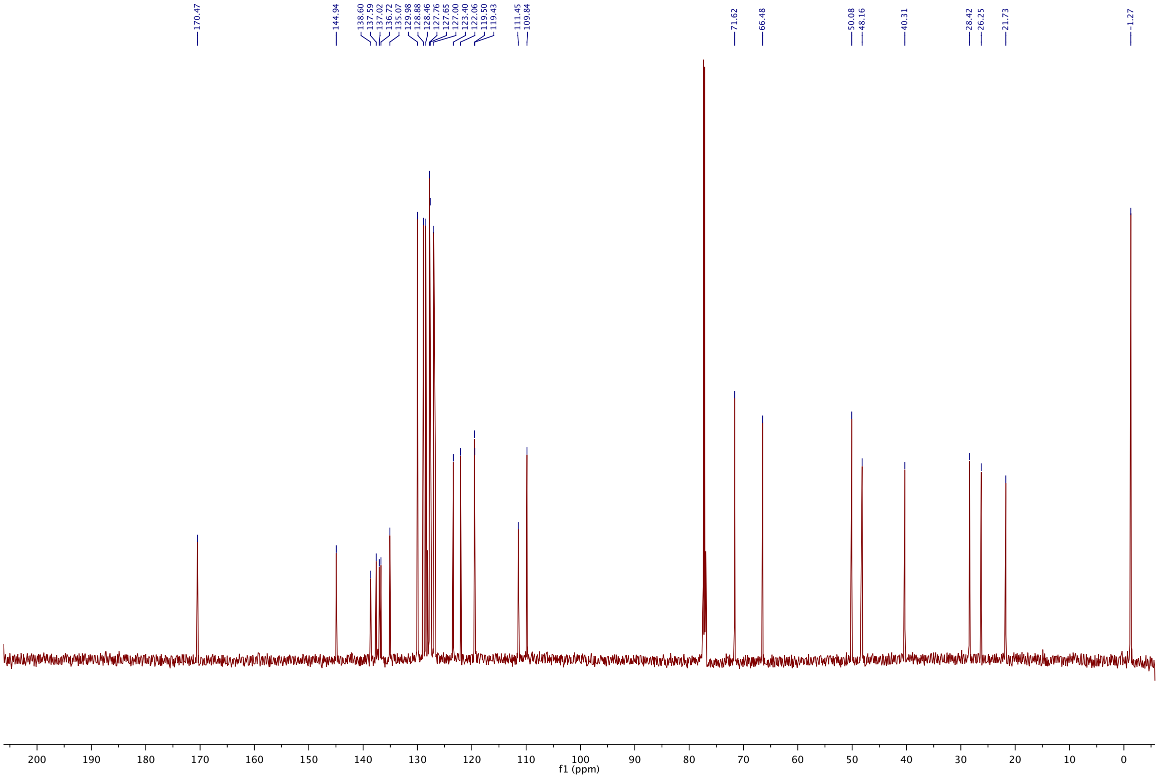

Hemiaminal S-9:

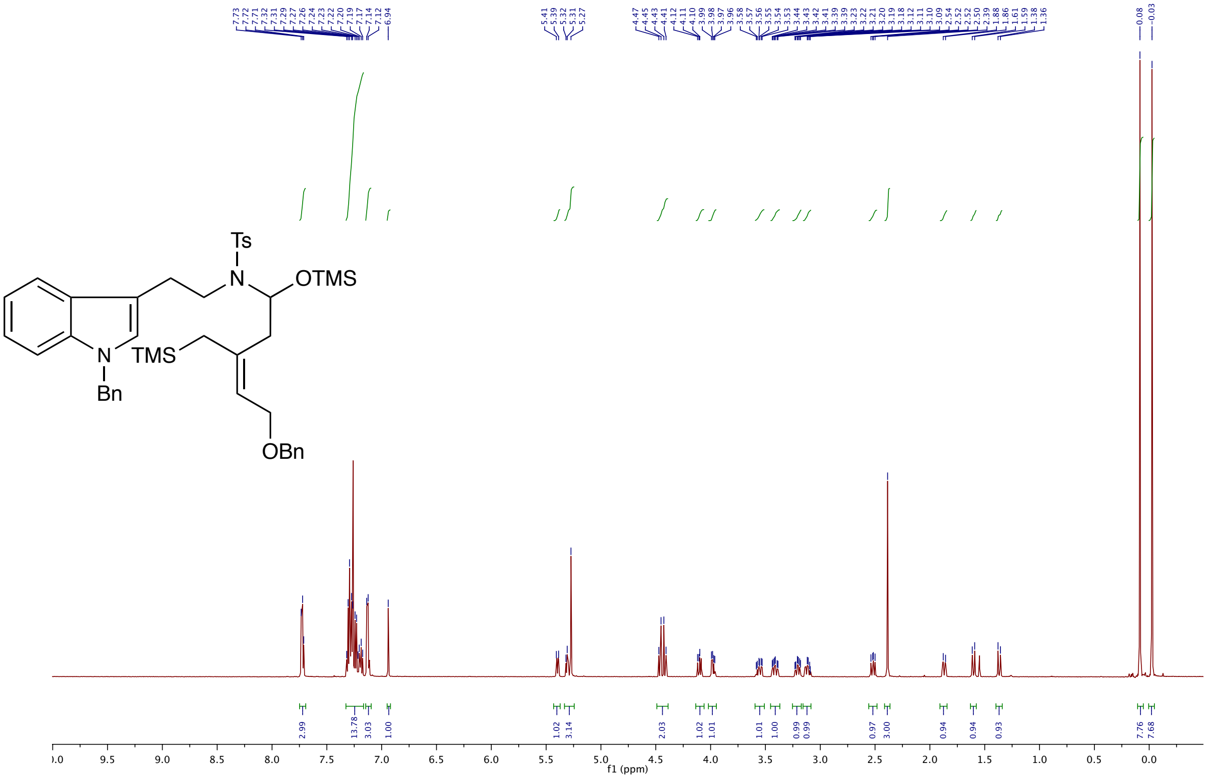

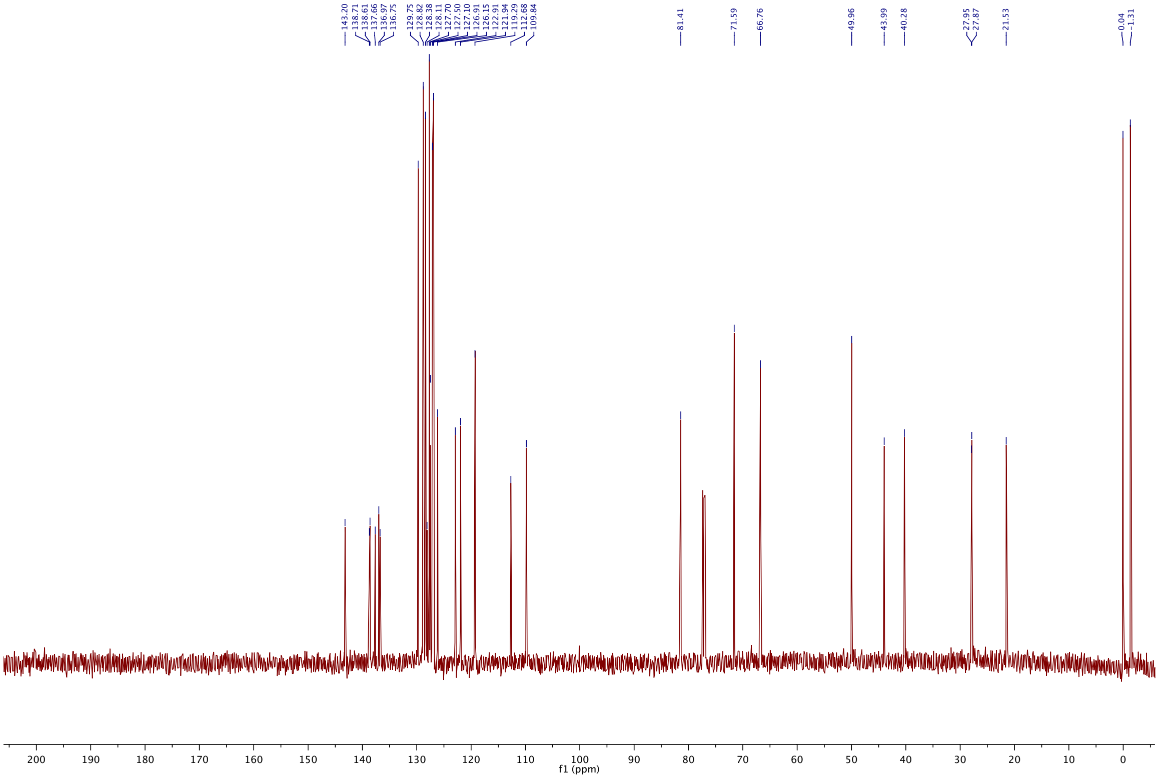

Tetracycle 20:

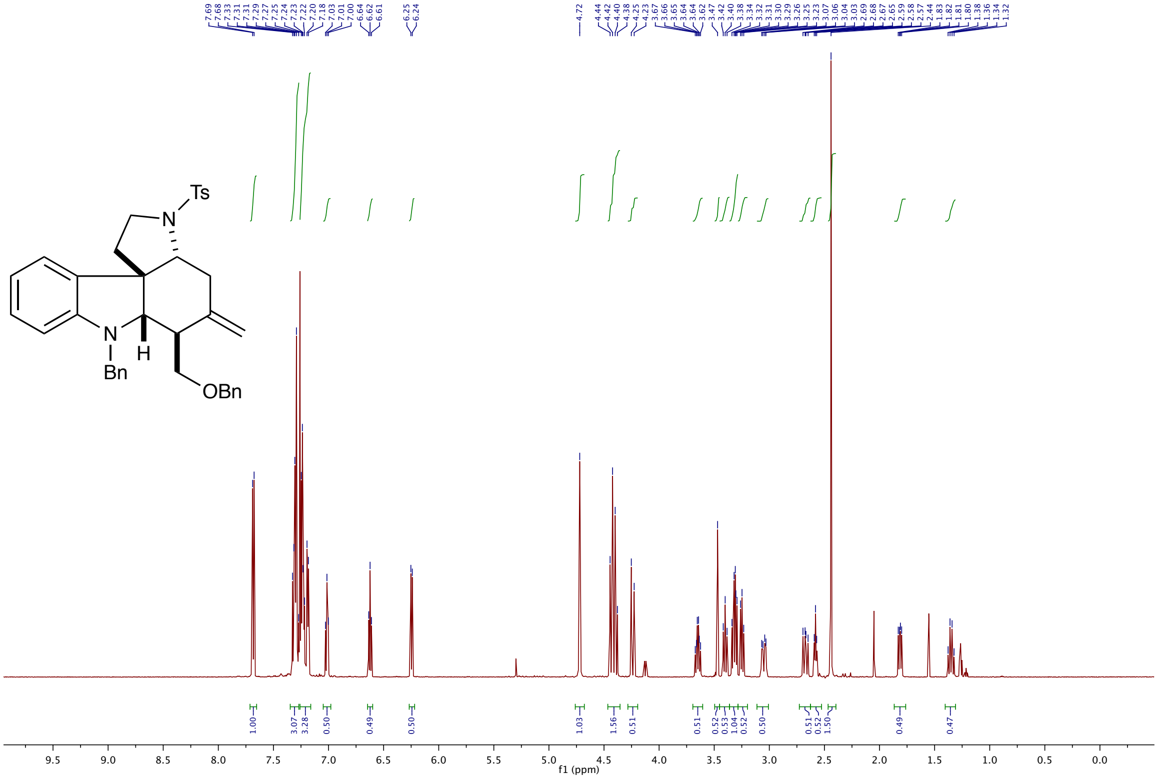

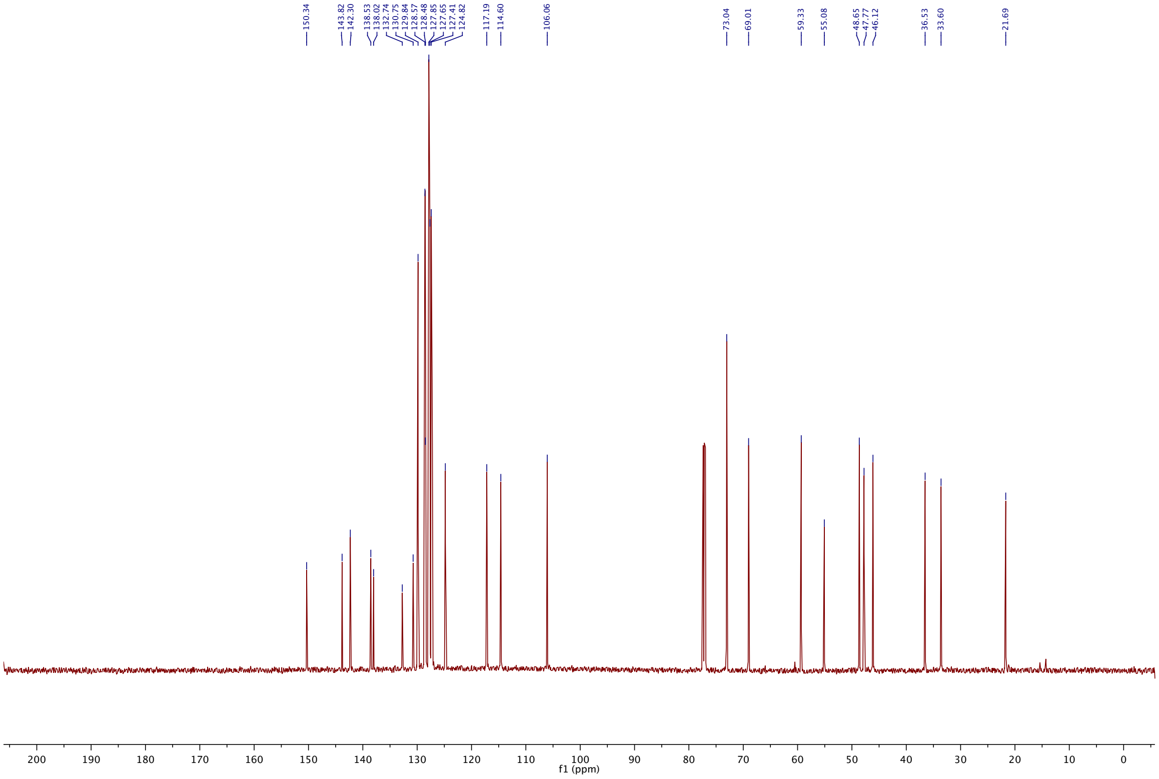

Ketone 21:

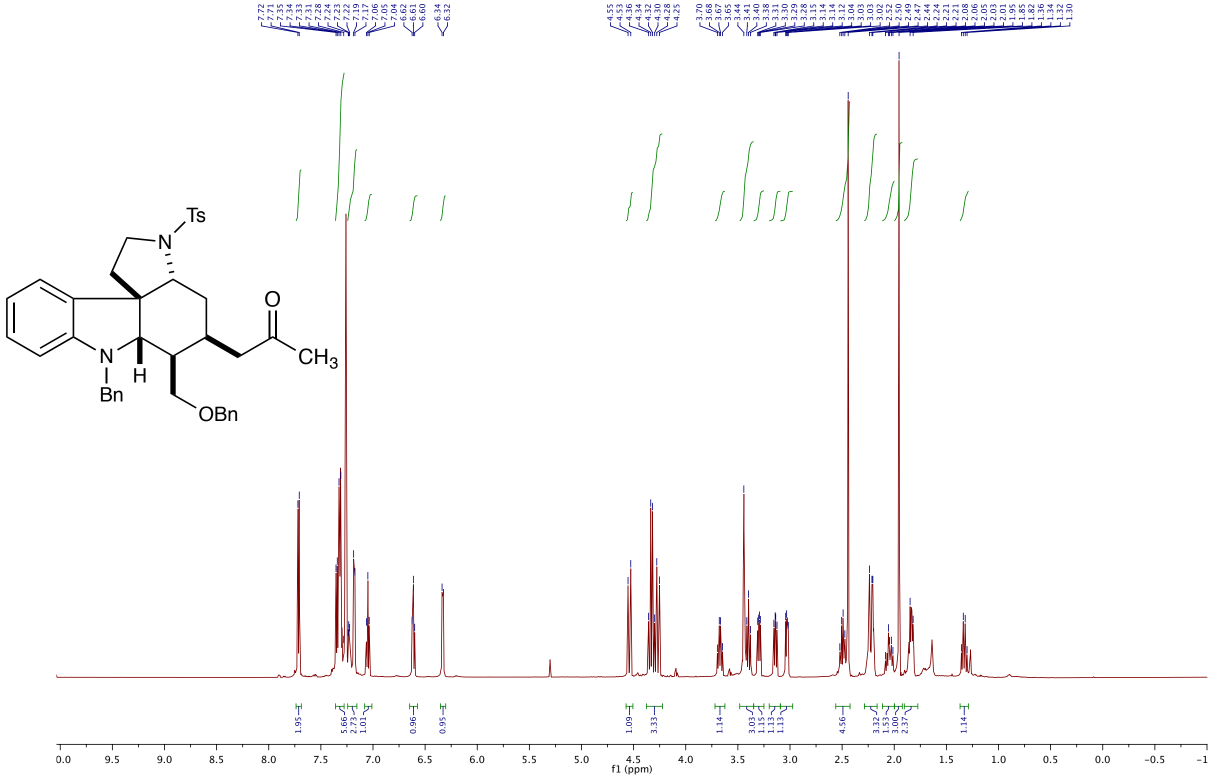

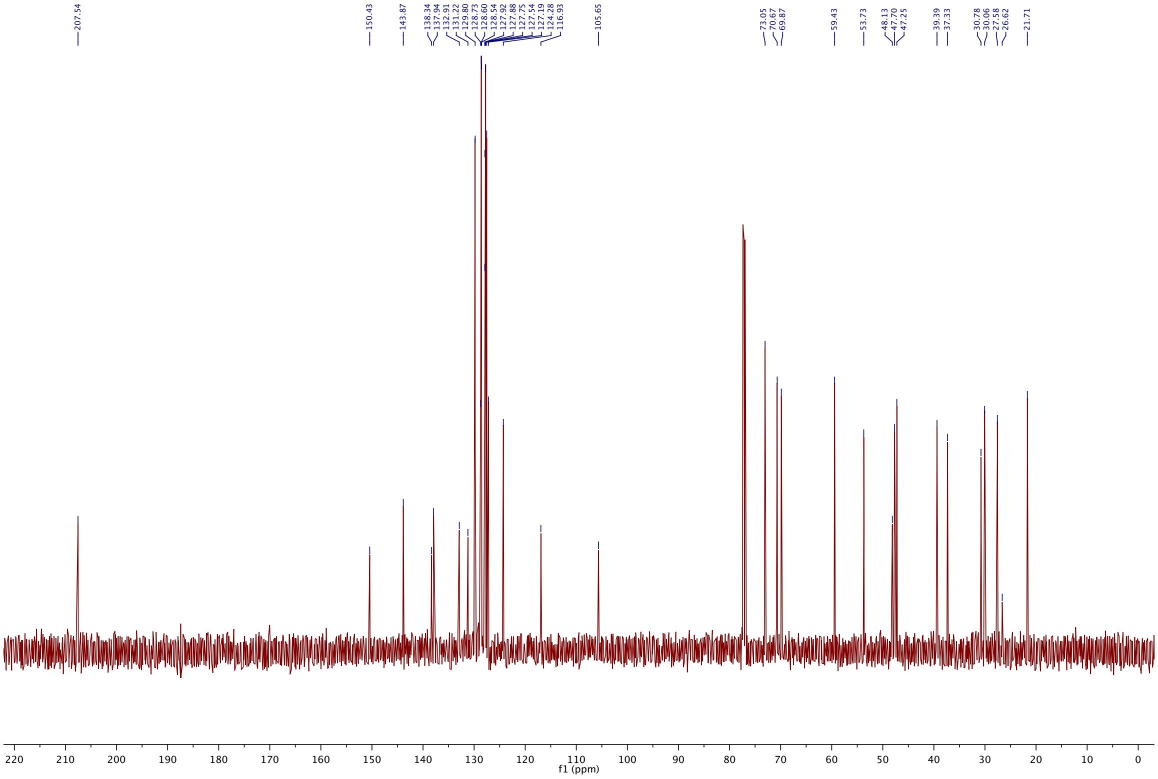

Dihydropyran 22:

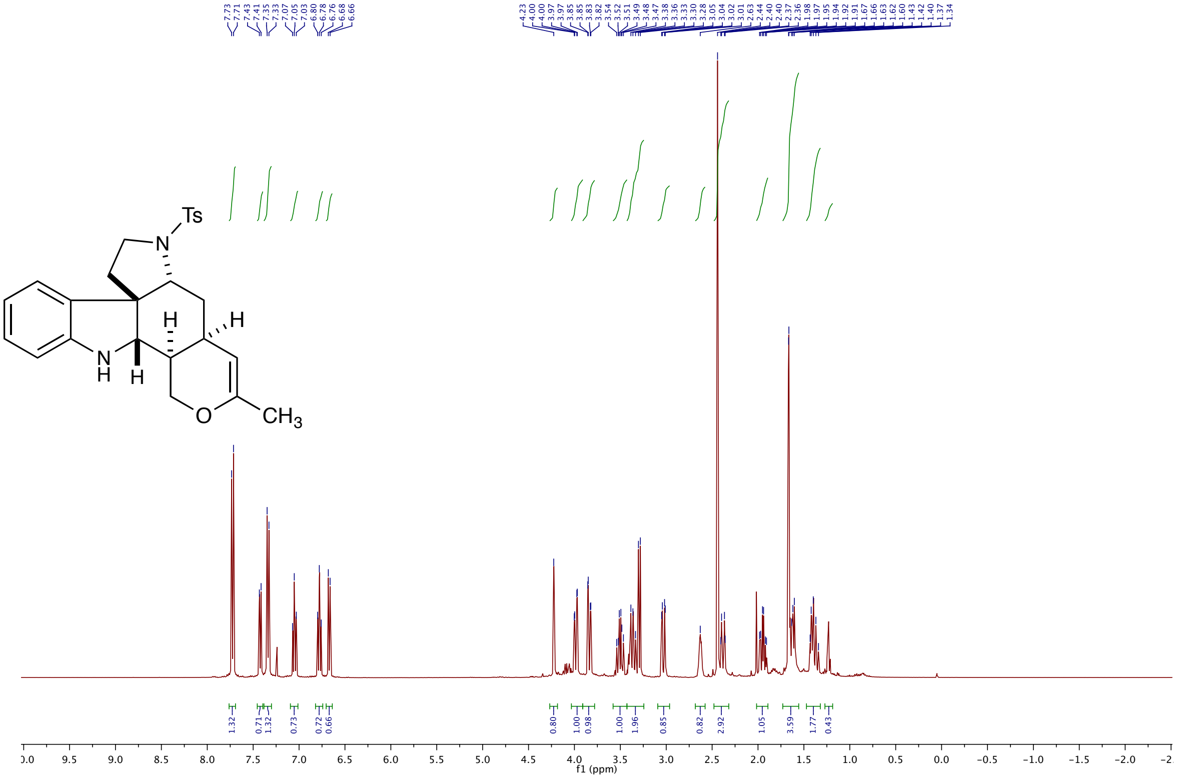

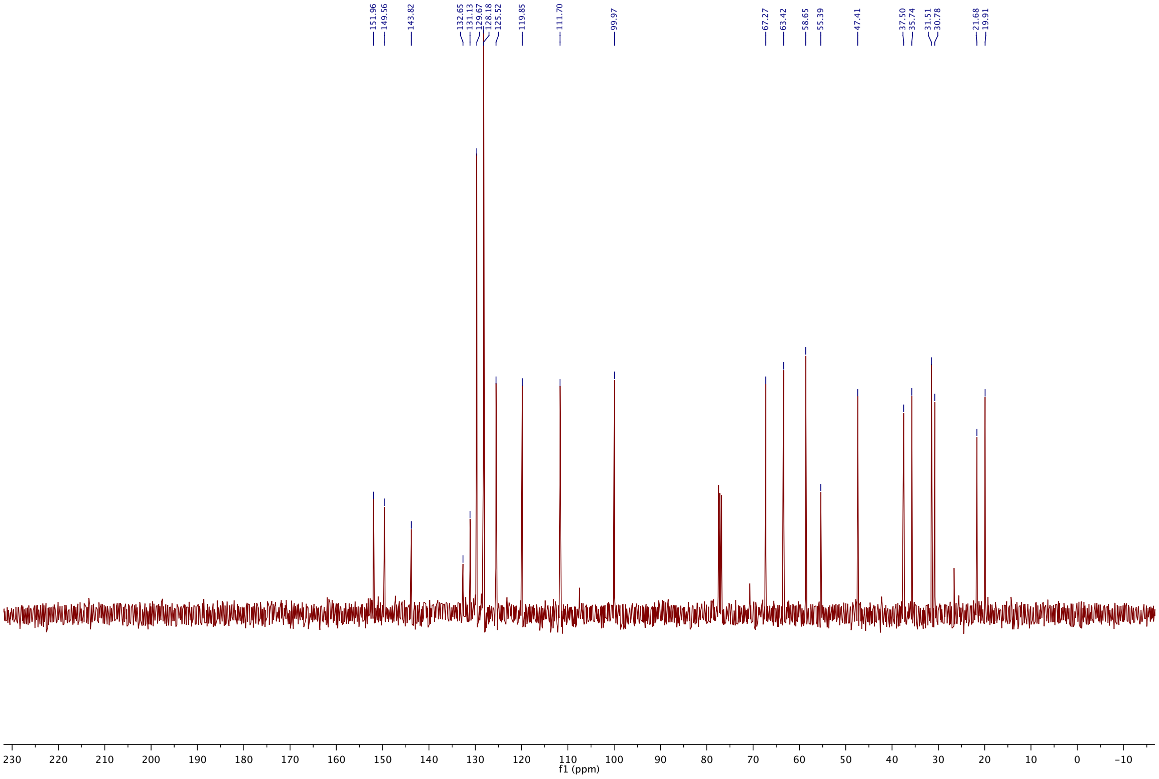

Acetamide 23:

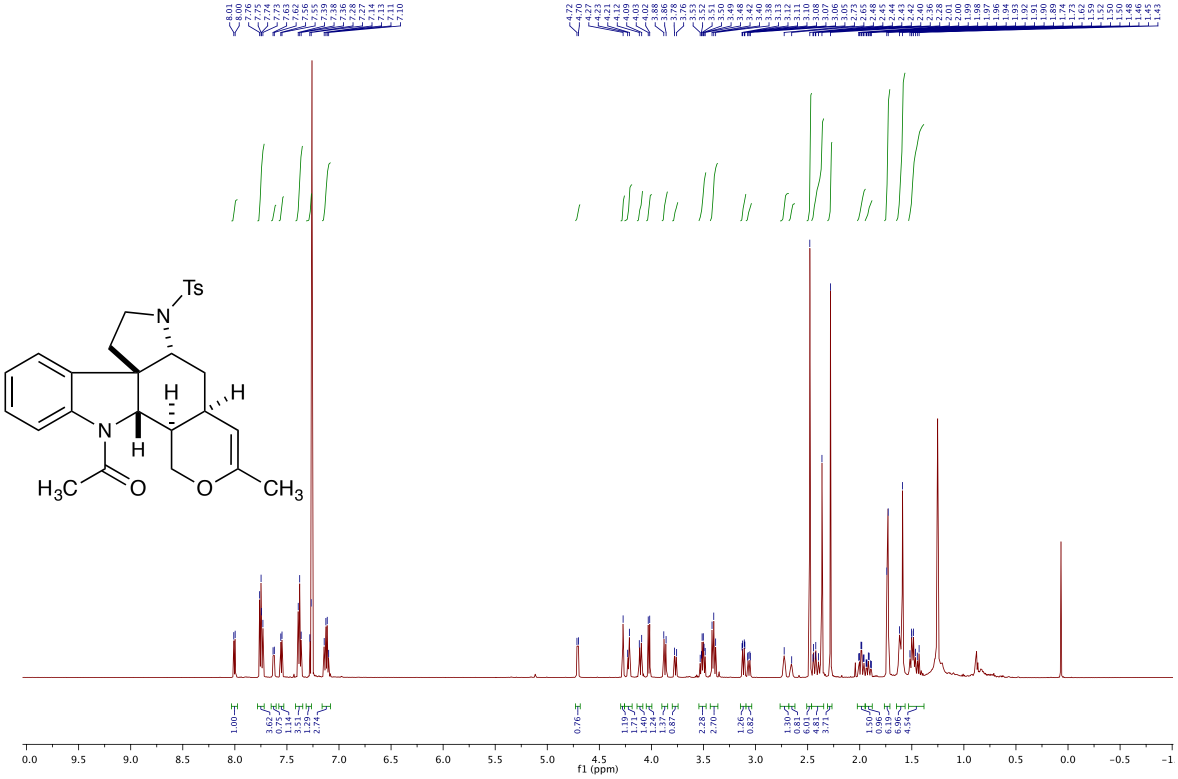

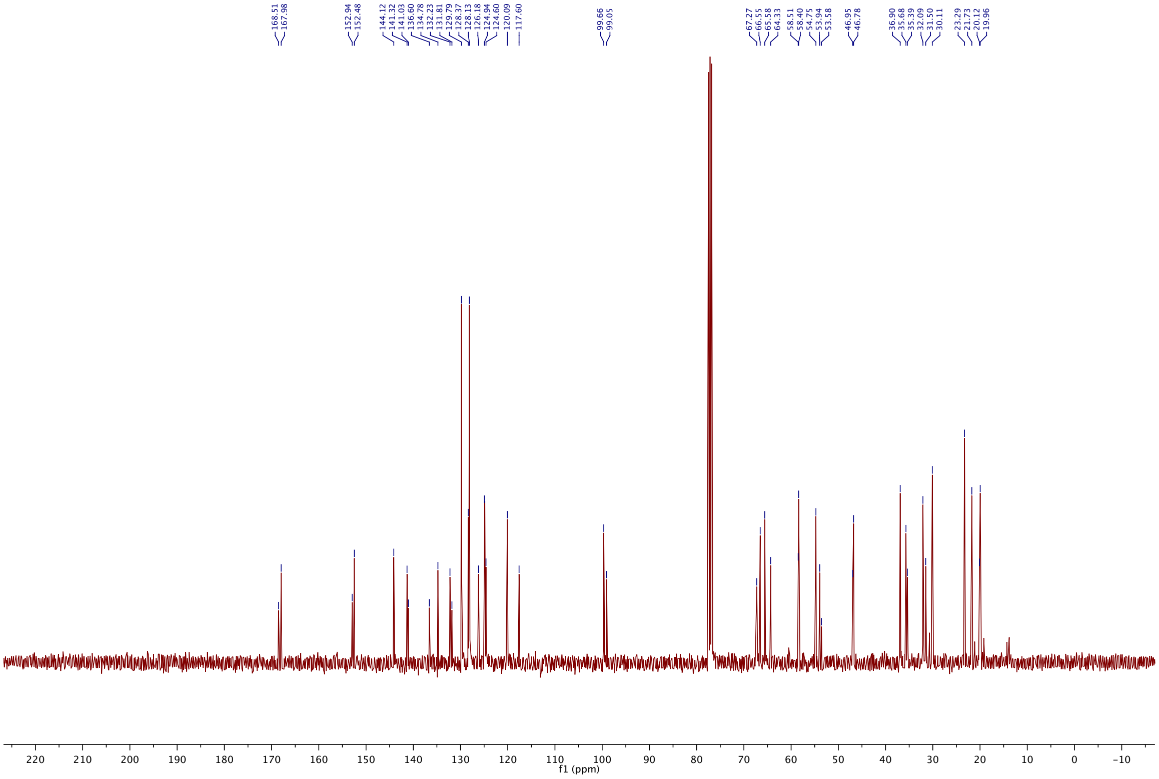

Trifluoromethylketone 24:

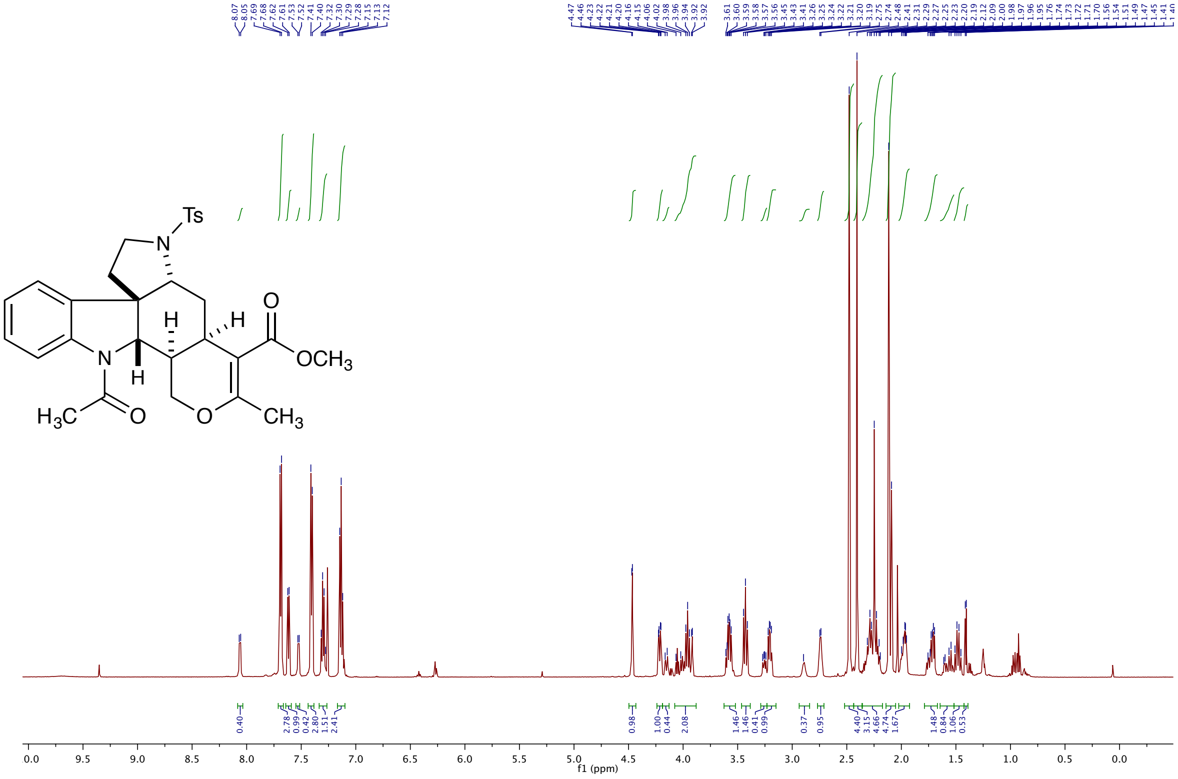

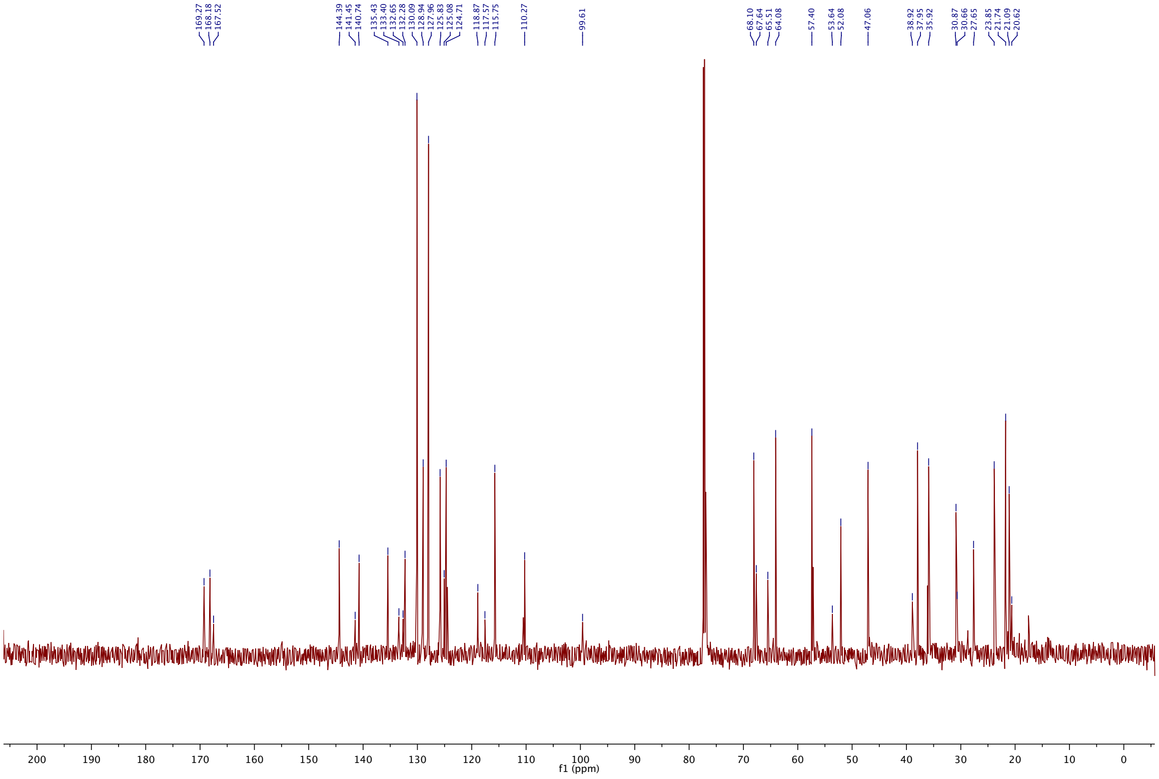

Ester 25:

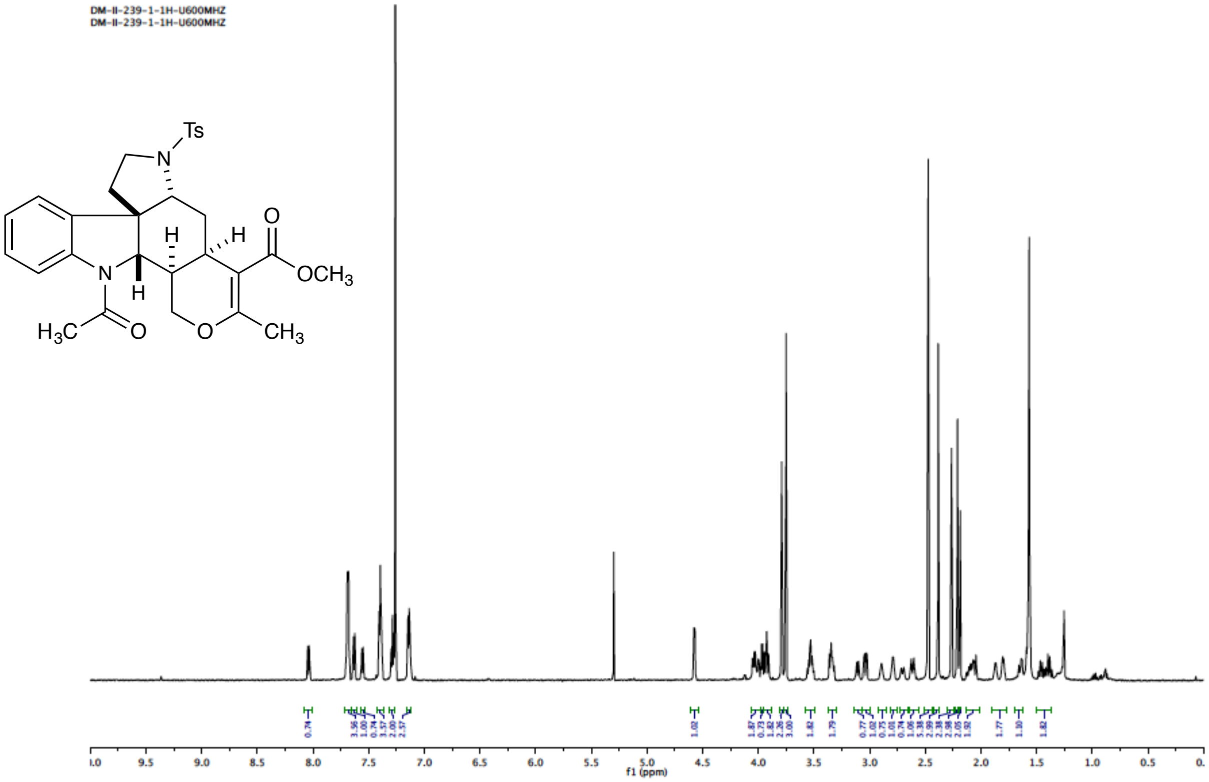
DM-II-239-1-1H-U600MHZ
DM-II-239-1-1H-U600MHZ
Ts
N
H
H
O
OCH3
N
H
H3C
O
O
CH3
f1 (ppm)

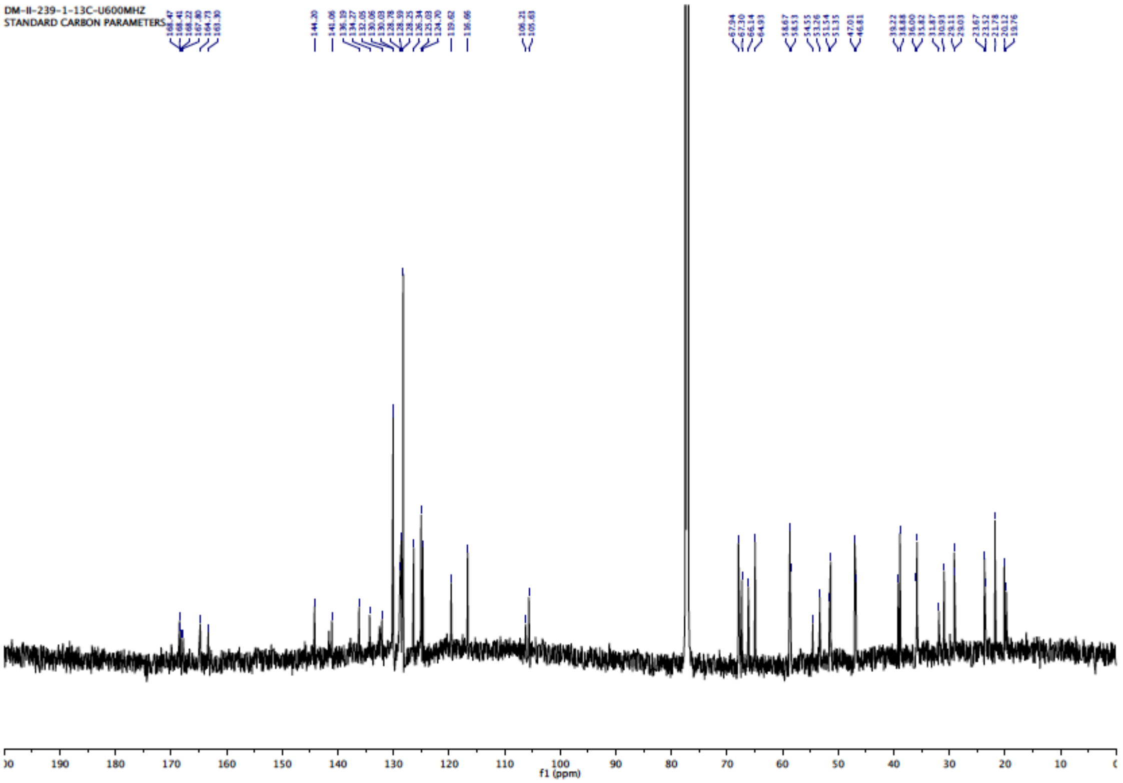
DM-II-239-1-13C-U600MHZ
STANDARD CARBON PARAMETERS
f1 (ppm)

N-tosylepimalagashanine 26:

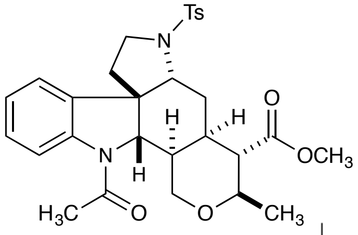

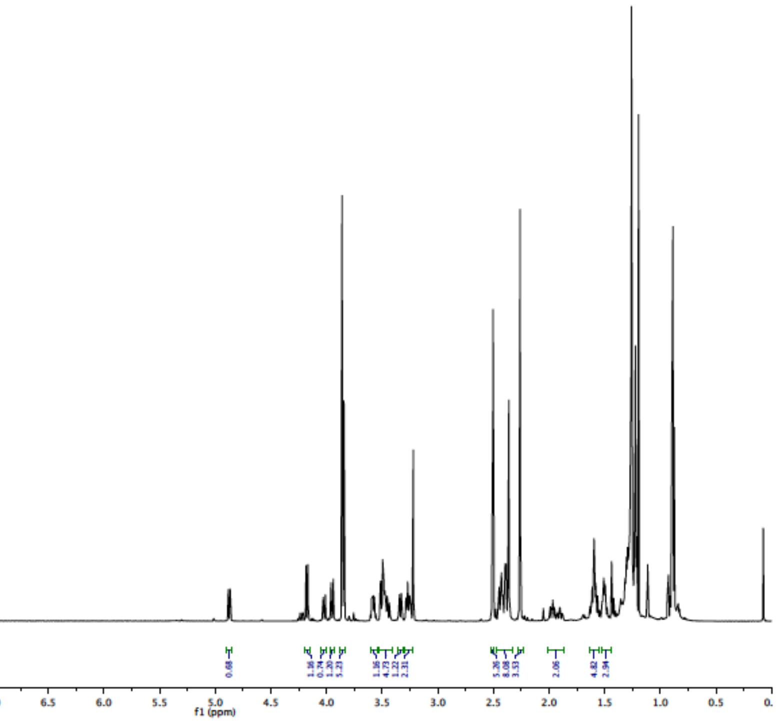

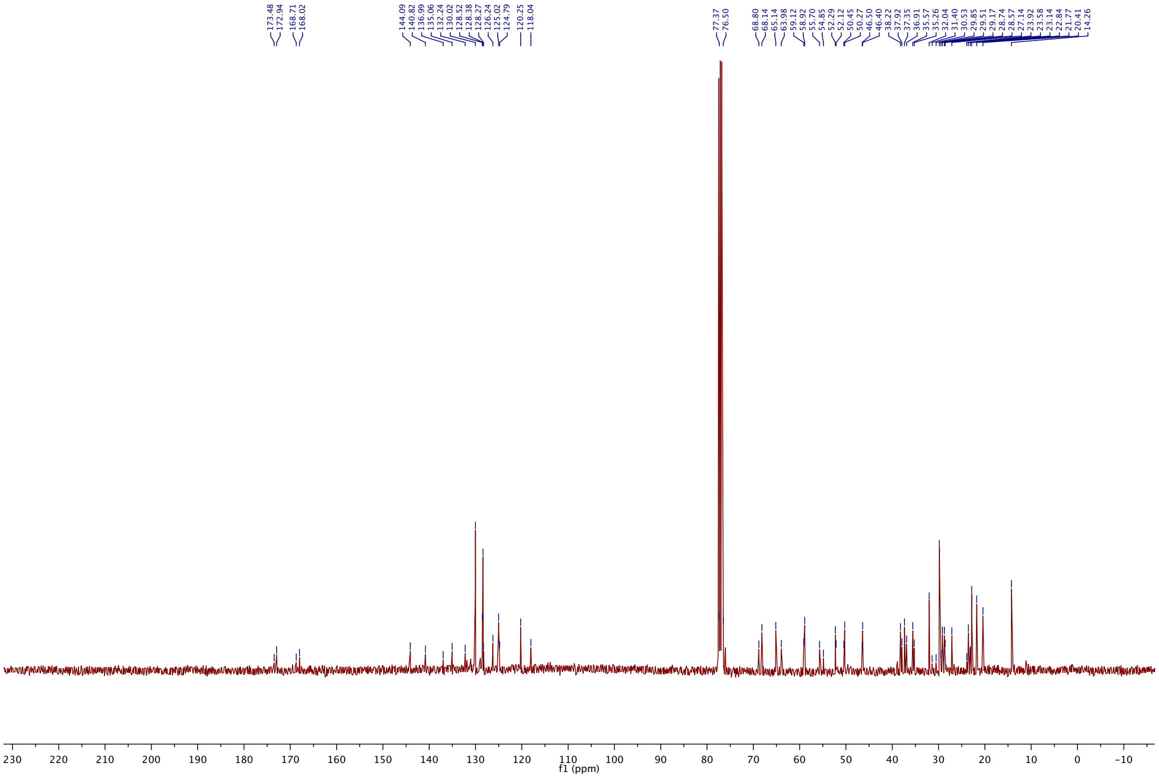

Epimalagashanine 27:

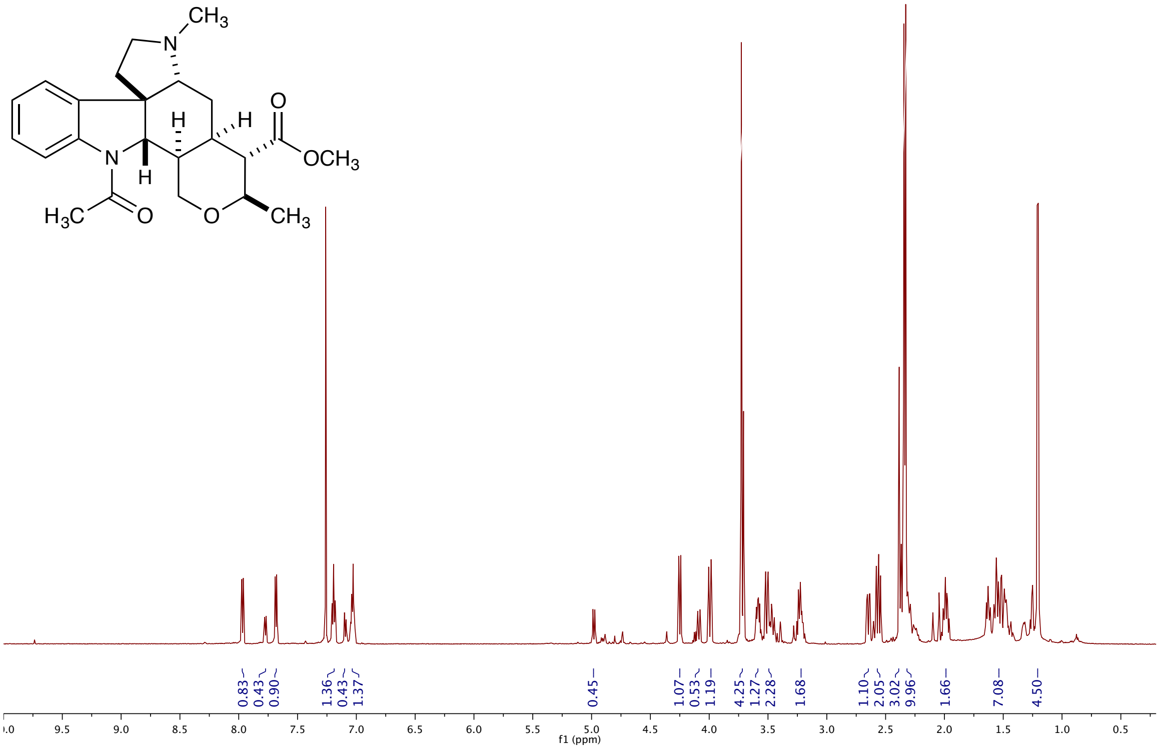

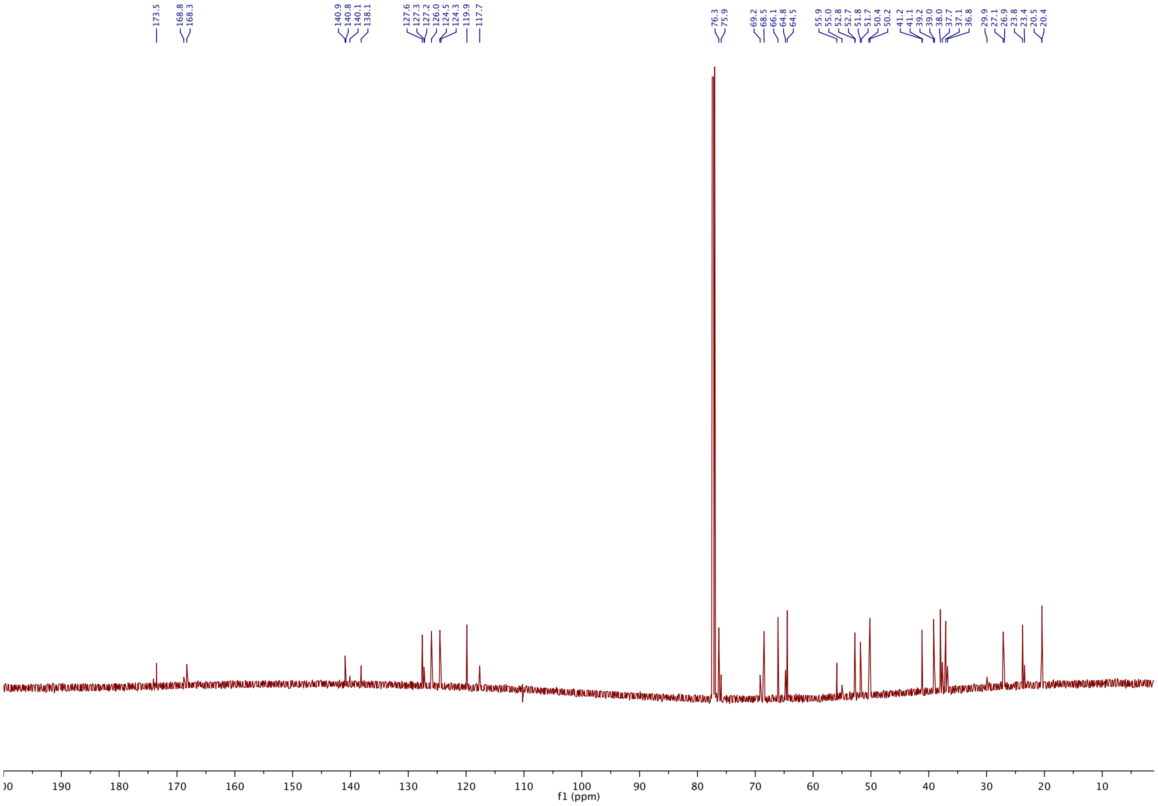

Methylamine 28:

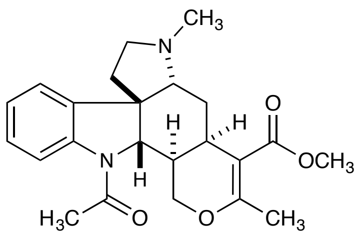

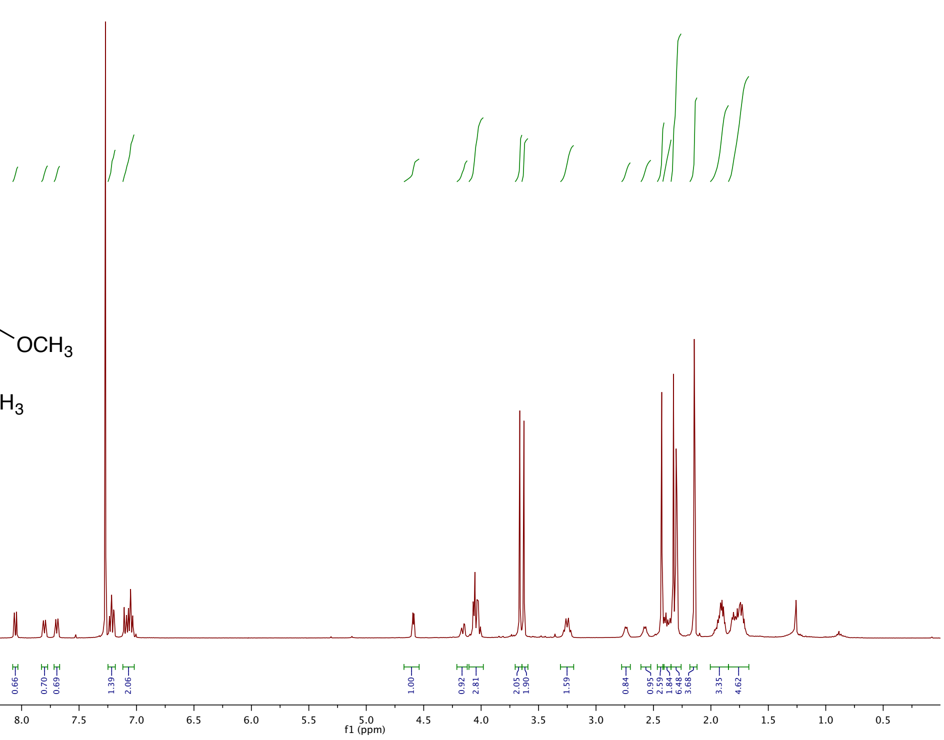

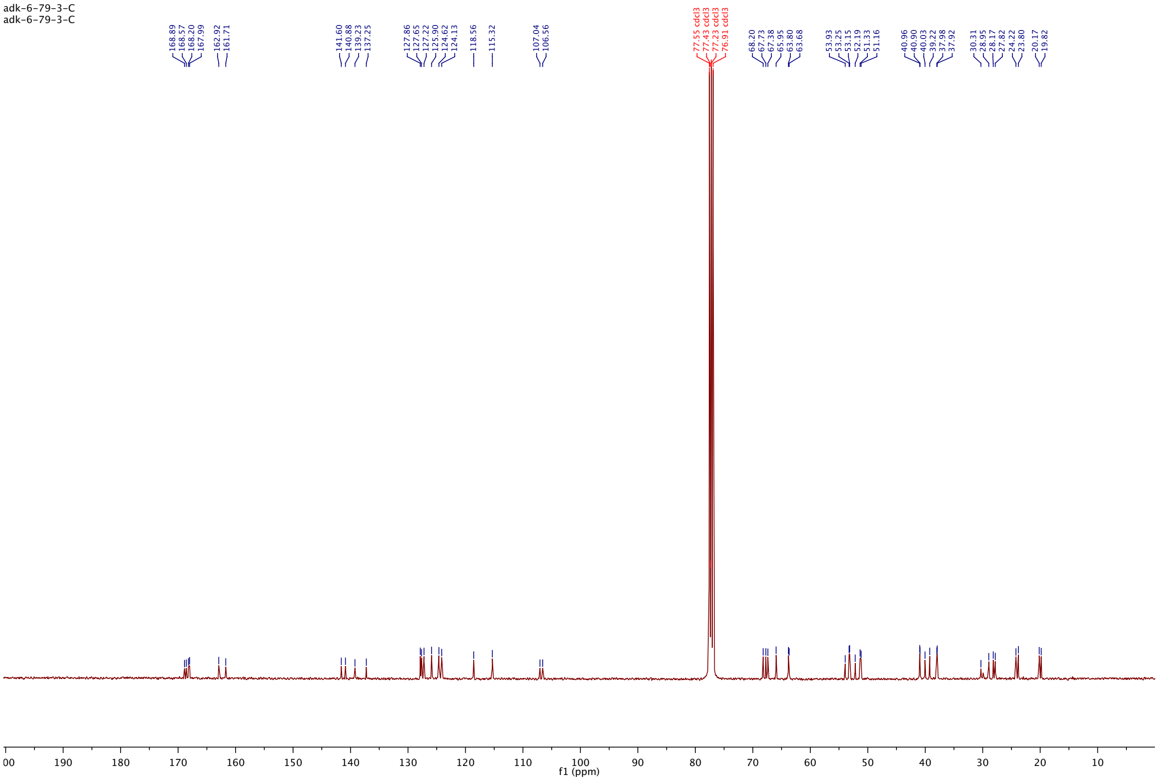

Malagashanine 1:

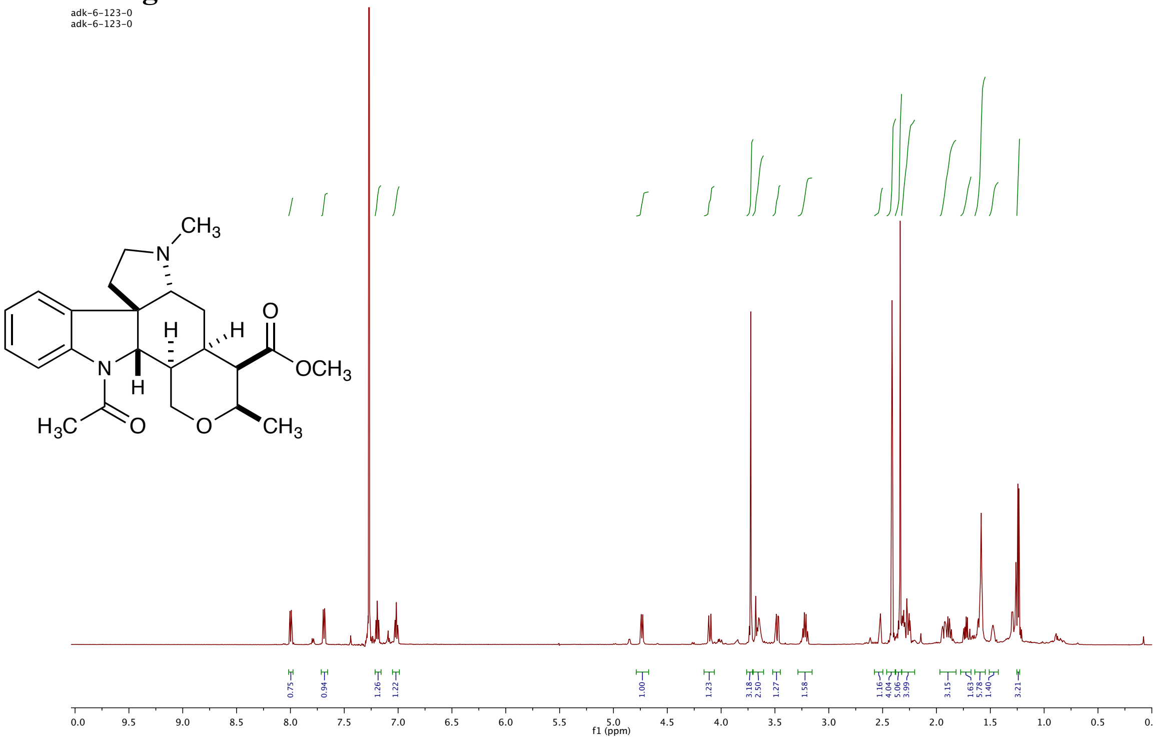

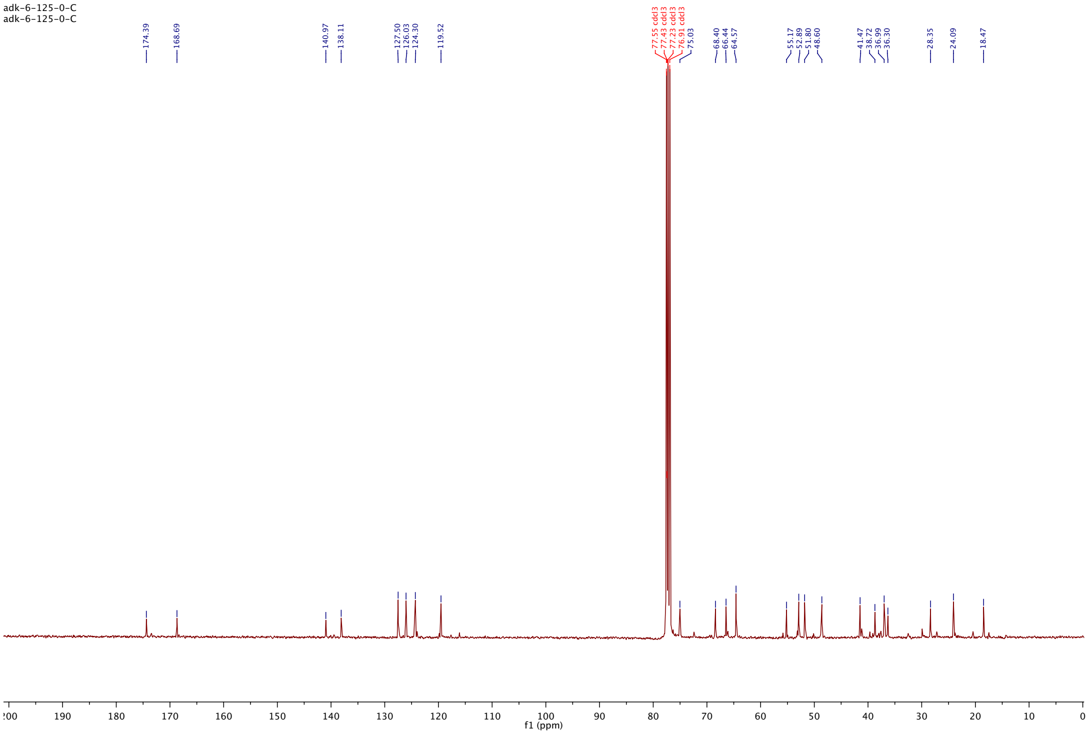

Supplement: Supplementary file 1 [file SC-008-C6SC03578G-s001.pdf]
